# Longitudinal peripheral DNA methylation remodeling following ketamine-assisted psychotherapy in major depressive disorder

**DOI:** 10.1080/15592294.2026.2731659

**Published:** 2026-10-15

**Authors:** Arghavan Nepton, Sonya C. Faber, Zachary Kaminsky, Mario Nucci, Hesam Farahani, Monnica T. Williams, Sergio R. Perez Rosal

**Affiliations:** a Department of Cellular and Molecular Medicine, University of Ottawa, Ottawa, ON, Canada; b School of Psychology, Faculty of Social Sciences, University of Ottawa, Ottawa, ON, Canada; c University of Ottawa Institute of Mental Health Research at the Royal, Ottawa, ON, Canada; d Field Trip Health Canada, Ottawa, ON, Canada; e Psychedelia-Stiftung, Berlin, Germany; f Klinik für Akut- und Notfallmedizin, Universitätsklinikum Ruppin-Brandenburg, Medizinische Hochschule Brandenburg Theodor Fontane, Neuruppin, Germany

**Keywords:** Ketamine-assisted psychotherapy, depression, DNA methylation, synaptic plasticity, epigenetic regulation, neuroimmune modulation

## Abstract

Ketamine produces rapid antidepressant effects, but the molecular processes associated with their persistence remain poorly defined in humans. We examined longitudinal DNA methylation changes in 10 adults with major depressive disorder undergoing ketamine-assisted psychotherapy using two pre-treatment assessments (T1, T2) and one post-treatment assessment (T3). Participants received three sessions of intranasal racemic ketamine within a structured psychotherapy protocol. Buccal DNA methylation was measured using the Illumina MethylationEPIC v2.0 BeadChip. Depressive symptoms improved markedly following treatment, with mean Beck Depression Inventory–II scores declining from the severe to the mild range (*p* < .001). Of 802,595 CpG loci evaluated, 20,770 met the nominal T3-versus-T2 screening criterion. Sequential filtering for methylation-change magnitude and movement relative to pre-treatment fluctuation identified 962 retained CpG loci, including 517 retained gene-annotated loci. A supplementary linear mixed-effects analysis modeled repeated measurements within participants across the 603 preselected gene-annotated magnitude-filtered loci; because these loci were selected using the same contrast and cohort, this analysis was not treated as independent confirmation. Exploratory enrichment analysis of this 603-locus set identified NOTCH1-related Reactome terms driven by NOTCH1, DLL1, and ADAM17. Within the retained signature, multiple core and adjacent Notch-related components formed a coherent signaling module spanning ligand, receptor processing, transcriptional regulation, and pathway cross-talk. These findings identify a structured peripheral methylation signature associated with ketamine-assisted psychotherapy. They do not establish brain-specific pathway activation or causality, but provide a longitudinal basis for investigating regulatory adaptations accompanying clinical improvement.

## Introduction

Ketamine is a rapid-acting antidepressant whose clinical efficacy is defined by a distinct temporal signature: a single sub-anesthetic dose initiates therapeutic effects within hours that persist far beyond the drug’s metabolic clearance. This primary action is driven by N-methyl-D-aspartate (NMDA) receptor antagonism, which triggers a glutamate burst and subsequent activation of alpha-amino-3-hydroxy-5-methyl-4-isoxazolepropionic acid (AMPA) receptors [1,2]. These events engage high-velocity signaling pathways, specifically the dephosphorylation of eukaryotic elongation factor 2 (eEF2) and the rapid translation of brain-derived neurotrophic factor (BDNF), culminating in a transient surge of synaptogenesis [3–5]. However, while this acute biochemical sequence is well-characterized as the requisite trigger for synaptic remodeling, a fundamental gap remains in our understanding of the molecular framework that stabilizes these nascent connections. Current neurobiological models have yet to fully explain how transient molecular events are translated into durable biological adaptations associated with sustained clinical response.

Establishing this enduring state likely requires a transition from transient signaling to stable, experience-dependent structural remodeling. Behavioral evidence suggests that ketamine reopens a transient, 48-hour critical period for social reward learning, providing a window for therapeutic re-adaptation [6]. Yet, because the initial signaling surges are inherently reversible, the persistence of the antidepressant effect implies a secondary regulatory layer capable of anchoring these changes within the neuronal architecture. Neuroepigenetic mechanisms, particularly DNA methylation, are uniquely positioned to serve this integrative role by converting short-lived pharmacological triggers into stable alterations in gene expression [7,8].

Crucially, the epigenetic response to ketamine appears to be highly selective. Identifying the downstream regulatory systems associated with this transition may help us in understanding how a transient intervention may contribute to a durable recovery. Against this background, human epigenetic studies provide a critical opportunity to examine how ketamine’s rapid neurobiological effects may be consolidated at the level of gene regulation. Most existing evidence for ketamine-associated epigenetic remodeling comes from animal models or postmortem brain tissue, which have demonstrated changes in methylation and chromatin accessibility within plasticity and stress-related genes following rapid-acting antidepressant exposure [7,9]. Far fewer studies have examined these processes longitudinally in living humans, particularly using within-subject designs capable of distinguishing treatment-related change from baseline variability. As a result, it remains unclear which epigenetic signatures reliably accompany clinical improvement following ketamine treatment, and whether these signatures map onto the same mechanistic domains identified in preclinical work.

Peripheral tissues offer a pragmatic and increasingly validated window into these processes. Although DNA methylation patterns are tissue-specific, prior work has shown that loci involved in stress physiology, endocrine regulation, and immune signaling often exhibit coordinated methylation changes across central and peripheral tissues, particularly in response to systemic perturbations such as pharmacologic intervention [8]. When combined with a within-person longitudinal design, peripheral sampling can capture biologically meaningful treatment-related change. This approach is especially relevant for ketamine, whose effects extend beyond the central nervous system to include the rapid normalization of neuroendocrine and inflammatory states.

The present study was designed to leverage these methodological advances by examining longitudinal DNA methylation changes in individuals with major depressive disorder (MDD) undergoing ketamine-assisted psychotherapy (KAP). By incorporating two pre-treatment baseline assessments, we aimed to estimate pre-treatment methylation fluctuation and evaluate post-treatment change relative to that baseline variation. We further applied a conservative effect-size and stability-based prioritization framework to identify CpG loci showing biologically meaningful change and organized the resulting loci into functional domains to facilitate biological interpretation.

Accordingly, the primary aim of this study was to characterize the epigenetic remodeling associated with KAP in humans. We hypothesized that treatment would not produce widespread methylation changes at upstream receptor or neurotransmitter genes, but would instead be associated with selective epigenetic remodeling of downstream regulatory systems involved in neuroplasticity, stress adaptation, and long-term cellular regulation. By integrating longitudinal methylation data with clinical outcomes, this study sought to identify treatment-associated molecular signatures that may contribute to durable therapeutic response.

## Methods

Fully detailed Methods can be found in the supplementary material.

### Participants and study design

This study was approved by the University of Ottawa Research Ethics Board UOREB#: H-03–23-8998. All procedures performed in this study involving human participants were conducted in accordance with the ethical standards of the institutional research committee and with the 1964 Helsinki Declaration and its later amendments. Written informed consent was obtained from all participants.

Data were collected from 10 adults diagnosed with MDD via the Mini International Neuropsychiatric Interview (MINI 7.0.2 [10]) and 12 healthy controls. MDD participants underwent a standardized KAP protocol consisting of three intranasal racemic ketamine sessions (180–270 mg) over approximately seven weeks. Controls provided two samples at a two-week interval and were processed alongside the MDD samples for quality-control purposes. Because controls did not undergo KAP and did not provide a third sample corresponding to the MDD post-treatment assessment, they were not included in the treatment-associated T3-versus-T2 methylation analyses.

### DNA methylation assessment and timepoints

Buccal DNA was collected at three timepoints: Baseline 1 (T1), Baseline 2 (T2;2 weeks after T1), and post-treatment (T3). The interval between T1 and T2 was used to quantify pre-treatment methylation fluctuation, while the change from T2 to T3 represented treatment-associated methylation movement. T1 was collected two weeks (14 days) before T2, establishing two pretreatment measurements separated by a fixed baseline interval. T2 was collected immediately before initiation of KAP. Participants then completed three ketamine dosing sessions within the KAP protocol over approximately five weeks, with T3 collected following completion of treatment. Accordingly, the interval from T1 to T3 was approximately seven weeks, comprising a two-week pretreatment baseline interval (T1–T2) followed by approximately five weeks from treatment initiation to the post-treatment assessment (T2–T3). Healthy control participants completed two biological assessments separated by two weeks (14 days) and did not receive ketamine or psychotherapy.

### Epigenetic processing and quality control

Genome-wide methylation was quantified using the Illumina MethylationEPIC v2.0 BeadChip. Raw data were processed using the SeSAMe QCDPB workflow. Probes with >10% missing values, cross-reactive probes, and those on sex chromosomes were excluded.

### Primary probe-level screening

For the primary probe-level analysis, methylation β-values were matched within participants between the immediate pre-treatment assessment (T2; Pool_ID ‘b’) and the post-treatment assessment (T3; Pool_ID ‘c’). At each CpG locus, a paired t-test was used to compare T2 and T3 methylation levels across the 10 participants with complete data. The resulting *p*-values were used as an exploratory screening measure. CpG loci with a nominal *p*-value below .05 were retained for subsequent magnitude and baseline-stability filtering. Because this initial screen was intended for candidate prioritization rather than epigenome-wide inference, the *p*-values were not interpreted as evidence of genome-wide statistical significance.

### Quantitative filtering of CpG loci

To prioritize biologically meaningful remodeling, we applied a multi-stage filter to loci meeting the nominal T3-versus-T2 screening criterion. Methylation differences and stability ratios were calculated from the group-level mean β-values of the 10 MDD participants at each timepoint. First, a magnitude filter was applied, retaining loci with Δmax ≥ .035, where Δmax represented the maximum absolute methylation difference across the T3–T1 and T3–T2 contrasts. This reduced the 20,770 loci to 1,180 magnitude-filtered loci. A stability filter was then applied using the ratio of treatment-associated change to baseline fluctuation, calculated as *R* = |T3 − T2| / |T2 − T1|. Loci with post-treatment methylation change at least three times greater than baseline fluctuation were classified as Baseline Stable Treatment-Responsive loci (BSTR; *R* ≥ 3; *n* = 692). Loci with treatment-associated change between two and three times greater than baseline fluctuation were retained as Dynamic Movers (2 ≤ *R* < 3; *n* = 270), while loci with *R* < 2 were excluded (*n* = 218). Functional annotation and category assignment were conducted only after quantitative filtering and did not influence locus selection (Figure 1). Following quantitative filtering, the 1,180 magnitude-filtered loci were mapped to available gene annotations, of which 603 had an associated gene annotation.

**Figure 1.**
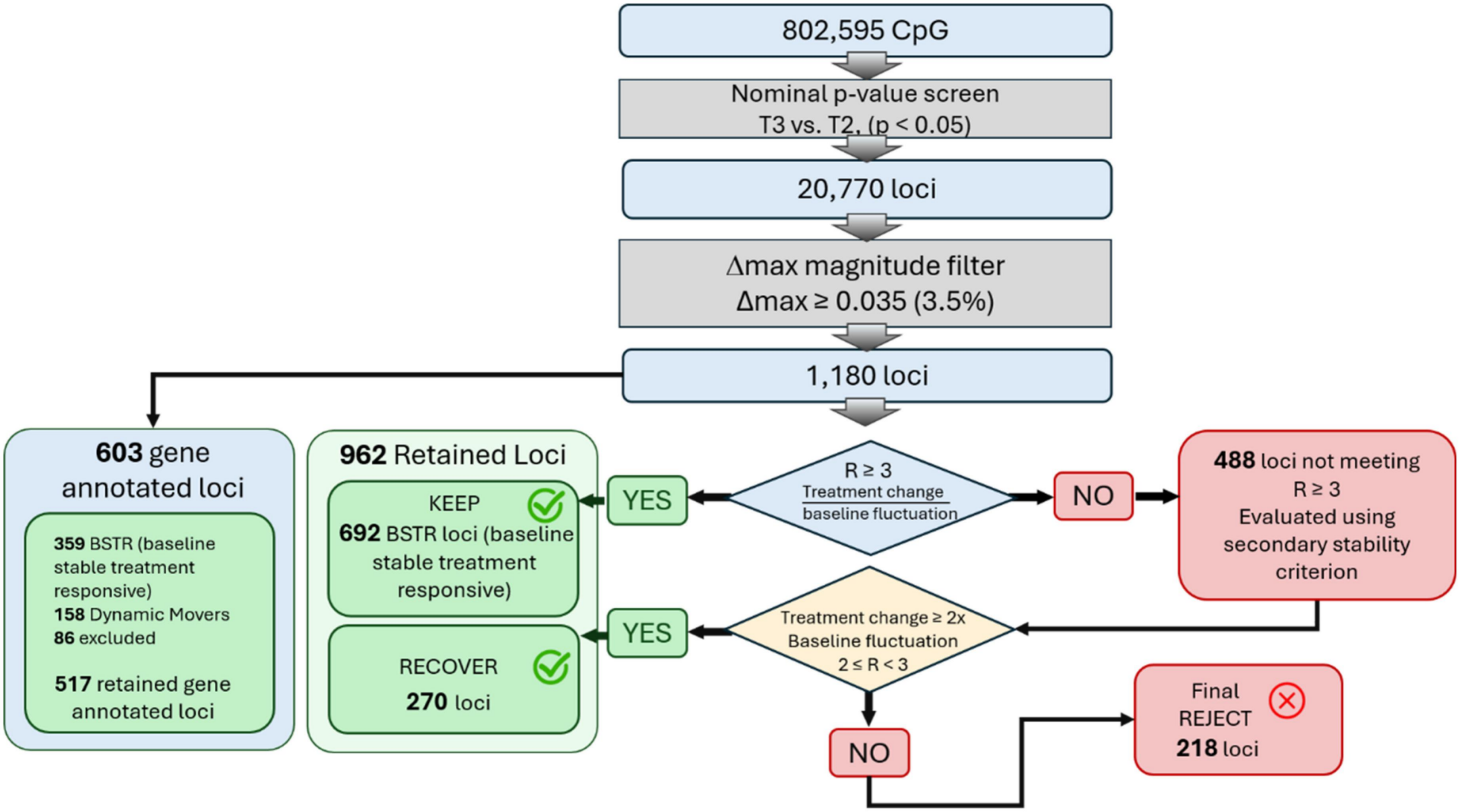
Quantitative filtering framework for treatment-associated CpG loci.

Following preprocessing, 802,595 CpG loci entered the downstream analysis. A nominal T3-versus-T2 *p*-value screen (*p* < .05) retained 20,770 loci. A magnitude filter was then applied using Δmax = max(|T3 − T1|, |T3 − T2|), with loci retained when Δmax ≥ 0.035, yielding 1,180 loci. Treatment-associated change was subsequently evaluated relative to pre-treatment fluctuation using the stability ratio *R* = |T3 − T2| / |T2 − T1|. Loci with *R* ≥ 3 were classified as Baseline Stable Treatment-Responsive loci (BSTR; *n* = 692). The 488 loci not meeting this primary stability criterion were evaluated using a secondary threshold; 270 loci with 2 ≤ *R* < 3 were retained as Dynamic Movers, while 218 loci with *R* < 2 were excluded. The final retained set comprised 962 CpG loci. Of the 1,180 magnitude-filtered loci, 603 had available gene annotations, including 359 BSTR loci, 158 Dynamic Movers, and 86 excluded loci, yielding 517 retained gene-annotated loci.

### Linear mixed-effects sensitivity analysis

To evaluate whether the identified methylation changes were also evident at the participant level, a supplementary linear mixed-effects analysis was conducted across the 603 gene-annotated loci entering stability classification. For each CpG locus, methylation β-values from T1, T2, and T3 were modeled with timepoint as a fixed effect and participant as a random intercept. The T3-versus-T2 contrast was used to evaluate treatment-associated methylation change.

### Gene annotation and functional enrichment

Exploratory functional enrichment analysis was performed using g: Profiler on the unique gene symbols mapped from all 603 gene-annotated magnitude-filtered loci. The full 603-locus set was analyzed rather than a manually selected subset to minimize pathway-selection bias. Because 86 of these loci did not meet the final stability criterion, the enrichment analysis was interpreted as characterizing the broader magnitude-filtered methylation set rather than the final retained signature.

### Psychological measures

Depressive symptoms were assessed at T_1_, T_2_, and T_3_ using the Beck Depression Inventory–II (BDI-II [11]). Secondary measures included the Childhood Trauma Questionnaire (CTQ [12]) and the NEO Five-Factor Inventory–3 (NEO-FFI-3)[13]) to assess early-life stress and personality traits, respectively.

## Results

### Baseline stability

Across all participants, the two pre-treatment assessments (T_1_ and T_2_) demonstrated high stability in depressive symptoms and personality domains, indicating no spontaneous shifts prior to KAP as per clinical measures of psychopathology. Baseline equivalence provided a clinical reference against which post-treatment changes could be evaluated.

### Depression symptoms

A repeated-measures ANOVA revealed a robust reduction in BDI-II scores following KAP. Because Mauchly’s test indicated that sphericity was violated, Greenhouse–Geisser-corrected results are reported, F(1.17, 10.50) = 55.67, *p* < .001, with a large effect size (η^2^ = .861). Pairwise contrasts showed no significant differences between baselines (*p* = .753), but significant improvements from baseline to post-treatment (both *p* < .001). Scores shifted from severe to mild ranges, consistent with literature describing ketamine’s rapid antidepressant effects mediated by glutamatergic modulation and synaptic potentiation [5] (Figure 2).

**Figure 2.**
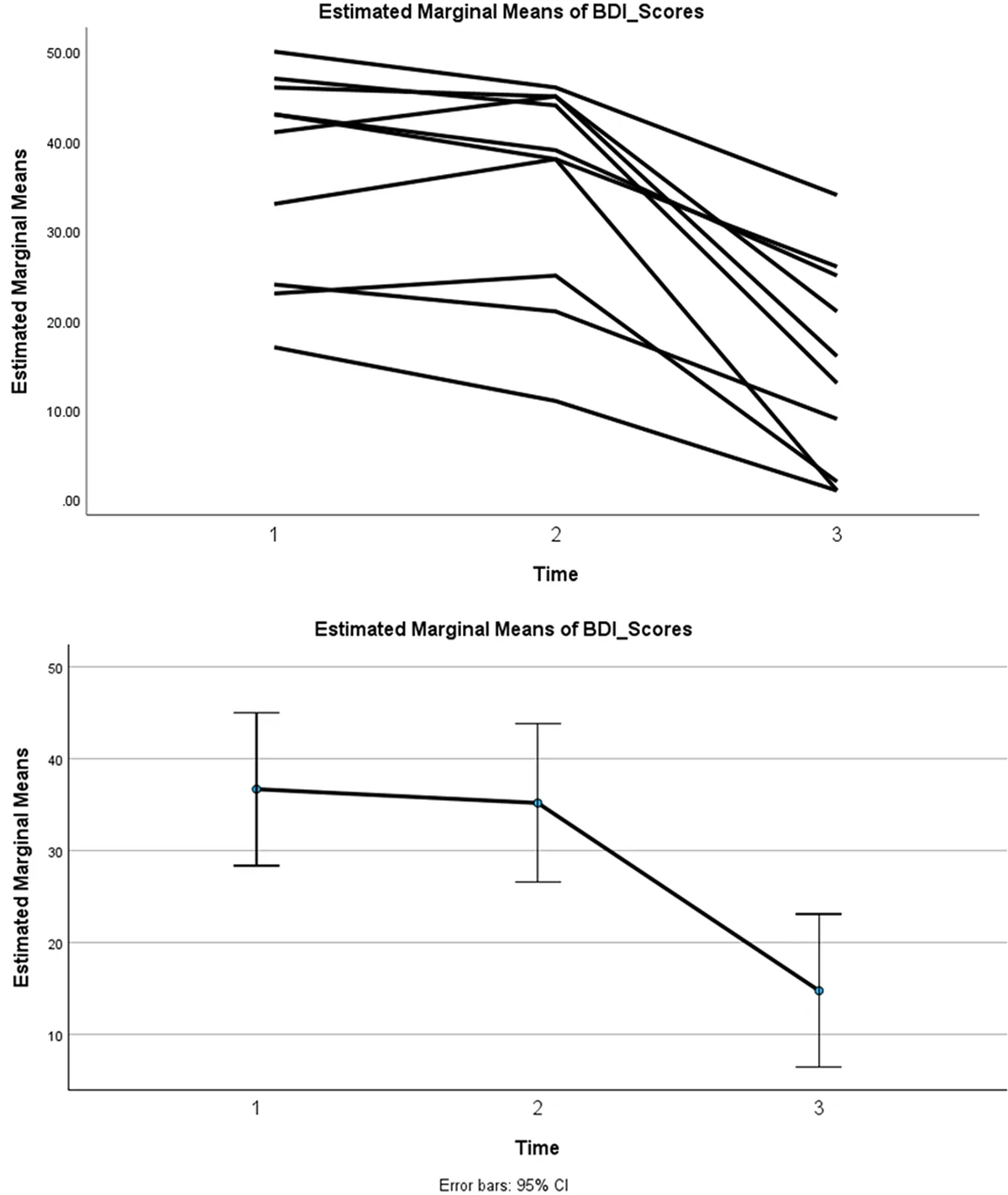
Changes in depressive symptoms following ketamine-assisted psychotherapy (KAP).

Individual participant trajectories. Spaghetti plot depicting each participant’s BDI-II scores across Time 1, Time 2, and Time 3. The figure illustrates high stability between the two baseline assessments and consistent within-person improvement following KAP, with all participants showing a downward trajectory from baseline to post-treatment. Mean depressive symptoms across time. Group mean BDI-II scores at the two pre-treatment baselines (Time 1 and Time 2) and post-treatment (Time 3). Baseline scores were stable, showing no significant difference between Time 1 and Time 2 (*p* = .753). Following KAP, depressive symptoms decreased sharply from the severe range (*M* = 36.7 at 1; 35.2 at 2) to the mild range post-treatment (*M* = 14.8), reflecting a large and statistically significant reduction (both 1→3 and 2→3 *p* < .001; repeated-measures ANOVA: F (1.17, 10.50) = 55.67, *p* < .001, η2 = .861). Error bars represent 95% confidence intervals.

### Genome-wide DNA methylation

#### Overview of epigenome-wide changes

Following preprocessing, 802,595 CpG loci were included in the downstream R analysis. Of these, 20,770 met the nominal screening criterion of *p* < .05 for the MDD T3-versus-T2 comparison. The genome-wide distribution of T3-versus-T2 methylation change was centered near zero, with a smaller tail of loci showing larger positive or negative changes. The T2-versus-T1 comparison provided a descriptive measure of methylation fluctuation during the pre-treatment interval, while comparison with the T3-versus-T2 distribution showed greater movement at a subset of loci following treatment (Figure 3).

**Figure 3.**
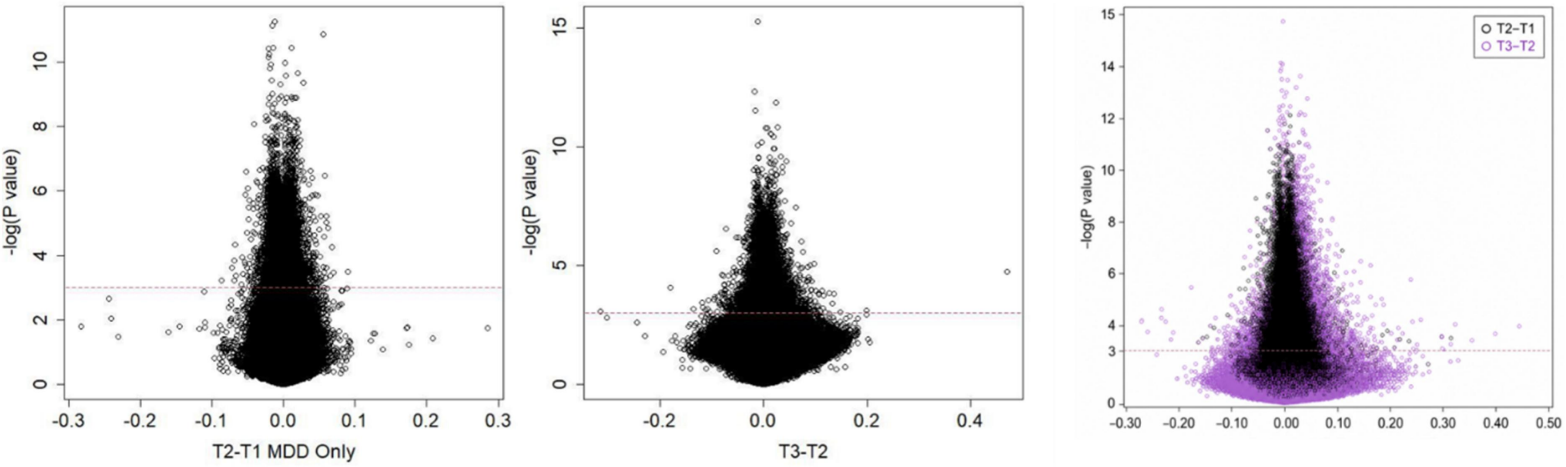
Genome-wide CpG methylation changes during the pre-treatment and treatment intervals. each point represents a CpG locus included in the downstream analysis. The x-axis shows signed methylation change (Δβ), and the y-axis shows statistical significance as −log10(p). left: T2-versus-T1 comparison, representing methylation fluctuation across the two pre-treatment assessments. middle: T3-versus-T2 comparison, representing treatment-associated methylation change from the immediate pre-treatment assessment to post-treatment. Of the 802,595 loci evaluated, 20,770 met the nominal screening criterion of *p* < .05 in the T3-versus-T2 comparison and formed the initial pool for subsequent magnitude and stability filtering. right: descriptive overlay of the T2-versus-T1 distribution in black and the T3-versus-T2 distribution in purple, plotted using the same axis limits to facilitate direct visual comparison. The overlay illustrates greater post-treatment movement at a subset of CpG loci.

### Identification of treatment-associated methylation signals

The analytical framework was designed to identify CpG loci exhibiting treatment-associated methylation changes that exceeded normal baseline fluctuation. Following preprocessing, loci demonstrating nominal evidence of differential methylation between the immediate pre-treatment assessment (T2) and post-treatment assessment (T3; *p* < .05) were retained for further evaluation, yielding an initial set of 20,770 loci. To filter out micro-changes and prioritize biologically substantial effects, an absolute magnitude filter was applied. For each locus, Δmax was defined as the maximum absolute methylation difference across the T3–T1 and T3–T2 contrasts. Loci were retained when this value reached at least 3.5% points (Δmax × 100 ≥ 3.5), reducing the set to 1,180 loci.

To distinguish treatment-associated movement from spontaneous baseline variation, a stability ratio (R) was calculated by comparing post-treatment methylation change with pre-treatment fluctuation:

*R* = |Δ treatment| / |Δ baseline|

or

*R* = |T3 MDD − T2 MDD| / |T2 MDD − T1 MDD|

The numerator represents the change from the immediate pre-treatment baseline (T2) to the post-treatment assessment (T3), while the denominator represents spontaneous drift between the two pre-treatment assessments (T1 and T2). Higher *R* values therefore indicated loci at which post-treatment methylation movement substantially exceeded baseline variability.

Loci were classified using a two-stage quantitative framework based on this ratio. Loci with treatment-associated movement at least three times greater than baseline fluctuation (*R* ≥ 3) were classified as Baseline Stable Treatment-Responsive (BSTR) loci, accounting for 692 loci. The remaining loci were evaluated using a secondary Dynamic Mover framework. Loci were retained as Dynamic Movers when post-treatment change remained at least twice, but less than three times, the magnitude of baseline fluctuation (2 ≤ *R* < 3), accounting for a further 270 loci. This secondary category was intended to preserve loci demonstrating substantial treatment-associated movement despite greater pre-treatment variability. Loci with *R* < 2 were excluded from subsequent analyses (*n* = 218). Application of this two-stage stability framework to the 1,180 magnitude-filtered loci yielded a final quantitative set of 962 retained loci, comprising 692 BSTR loci and 270 Dynamic Movers.

CpG loci lacking gene annotations were excluded from gene-based biological interpretation. Among the 1,180 loci entering stability classification, 603 had available gene annotations. These included 359 BSTR loci, 158 Dynamic Movers, and 86 loci with *R* < 2. The final gene-annotated treatment-associated signature therefore comprised 517 retained CpG loci. All 603 gene-annotated loci evaluated during stability classification, together with their quantitative values and filtering outcomes, are detailed in Supplementary Table 1.

The fixed magnitude threshold of 3.5% points was applied before calculation and interpretation of the stability ratio. This criterion was intended to distinguish biologically meaningful treatment-associated signals from changes more plausibly attributable to technical or stochastic measurement variation. Technical-replicate studies of the Illumina Infinium MethylationEPIC platform demonstrate high reproducibility of β-value measurements, while also identifying variability in probe-level performance [14]. A conservative threshold of 3.5% points was therefore selected to prioritize methylation changes of sufficient magnitude for subsequent stability analysis. At the same time, the threshold was set below the differences greater than 10% sometimes observed in association with strong environmental exposures, thereby avoiding the exclusion of the more modest effect sizes, frequently in the range of approximately 1–7%, that characterize many intervention- and disease-associated methylation signals in array-based studies. The absolute-magnitude filter also constrains a known instability of the ratio R, which can become spuriously large when both its numerator and denominator are very small. Requiring Δmax ≥ .035 ensured that the stability ratio was evaluated only for loci demonstrating methylation movement of plausible biological magnitude, preventing sub-noise fluctuations from satisfying the retention criteria.

### Linear mixed-effects sensitivity analysis

Linear mixed-effects models were fitted for all 603 gene-annotated loci across the 10 MDD participants with complete methylation measurements at T1, T2, and T3. All 603 loci showed a nominally significant T3-versus-T2 contrast at *p* < .05. Because the loci were preselected using the T3-versus-T2 contrast in the same cohort, these models were not independent significance tests. They were used only to examine whether the direction and magnitude of the selected changes remained evident when within-participant dependence was modeled explicitly. Full model results are provided in Supplementary Table 2.

### Functional enrichment analysis

Exploratory functional enrichment analysis was performed using g: Profiler on the unique gene symbols mapped from all 603 gene-annotated magnitude-filtered loci. Analysis of the complete set, rather than a manually selected subset, was used to minimize pathway-selection bias. Two significant, closely related Reactome terms concerning NOTCH1 signaling in the context of the NOTCH1 t(7;9) translocation mutant were identified, both driven by overlap with NOTCH1, DLL1, and ADAM17. Significant enrichment was also observed for the broad Gene Ontology biological-process term multicellular organismal process and several transcription-factor motif terms. Because 86 of the 603 loci did not meet the final stability criterion, these findings characterize the broader magnitude-filtered methylation set rather than the final retained 517-locus signature.

### Identification of a Notch-associated signaling module

Following the exploratory enrichment analysis, a Notch-associated signaling module was examined in greater detail because the retained gene-annotated set included multiple components of canonical Notch signaling spanning extracellular signaling, receptor processing, intracellular signal integration, and transcriptional regulation (Figure 4A).

**Figure 4.**
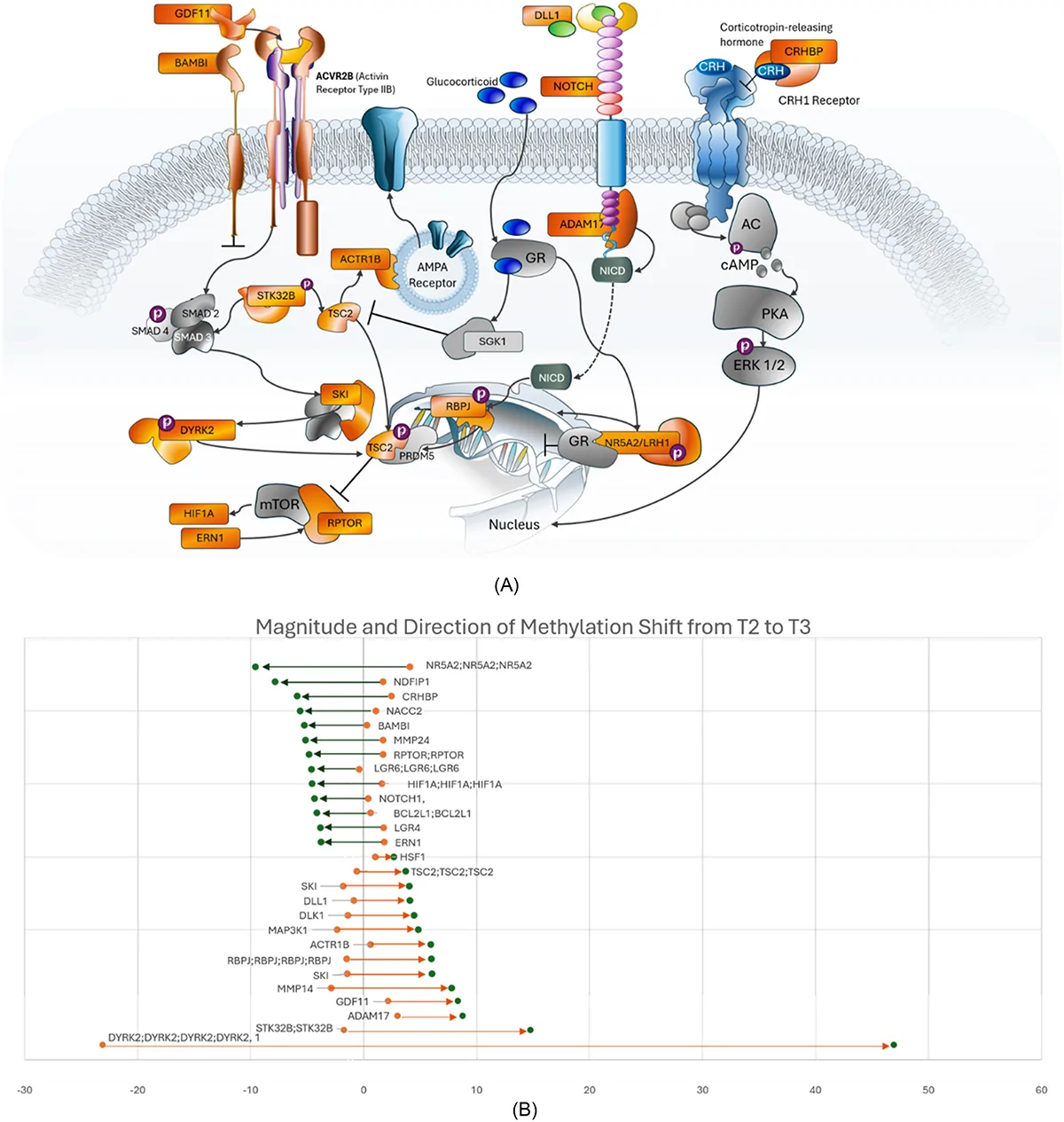
Mechanistic organization and longitudinal methylation trajectories of representative Notch-associated signaling cluster genes. (A) Literature-based mechanistic model illustrating known biological relationships among selected genes represented in the retained methylation signature and additional pathway components included for context. For each gene included in the model, established upstream and downstream signaling relationships were manually checked against the published scientific literature. Orange labels indicate genes represented by retained treatment-associated CpG loci. Grey labels and other pathway elements are included for biological context and were not represented in the final retained signature. The figure presents a selected mechanistic subset rather than all retained genes. This mechanistic model was developed after quantitative filtering and was not used for gene selection or prioritization. (B) Trajectory plot showing longitudinal methylation change at representative CpG loci mapped to genes in the Notch-associated module. Each row on the y-axis represents one CpG locus, labelled by its associated gene. The x-axis shows methylation change relative to T1, which was set to zero for every locus, thereby displaying relative longitudinal movement rather than absolute β-values. Orange points indicate the T2 position relative to T1, and green points indicate the T3 position relative to T1. Arrows show the direction and magnitude of change from T2 to T3. Values on the x-axis are expressed as percentage-point changes in methylation.

During the preparation of this work the authors used a generative artificial-intelligence assistant (a large language model) to support language editing, numerical auditing of reported values against the primary call matrices, and verification of genomic annotations against public reference resources. The tool was not used to generate primary research data or results. All AI-assisted text and analyses were reviewed and verified by the authors, who take full responsibility for the content of the article.

Exploratory Reactome enrichment analysis of the unique gene symbols mapped from the 603 gene-annotated loci identified two significant, closely related NOTCH1-associated terms concerning constitutive and nonconstitutive signaling by the NOTCH1 t(7;9) translocation mutant. Both terms had an adjusted *p*-value of .0249 and were driven by overlap with NOTCH1, DLL1, and ADAM17. These database annotations were interpreted as supporting representation of NOTCH1-related signaling machinery within the set, rather than as evidence of the translocation or oncogenic NOTCH1 activation in participants.

To assess representation of canonical Notch signaling machinery within the broader treatment-associated methylation signature, genes were classified as either core Notch components or adjacent Notch regulators. Core Notch components included the NOTCH1 receptor, the DLL1 ligand, and the transcriptional effector RBPJ. Adjacent regulators included ADAM17, BAMBI, and SKI, which are involved in receptor processing and signaling cross-talk with related regulatory pathways.

At the extracellular and membrane interface, the Notch-associated signaling cluster included NOTCH1, DLL1, BAMBI, and ADAM17. These genes were represented by retained CpG loci demonstrating treatment-associated methylation changes that exceeded baseline variability and occupied central positions within the identified signaling network (Table 1).

**Table 1.** Core Notch and adjacent Notch signalling components.

Gene	R-Ratio	Δmax (%)	Pathway Role
BAMBI	16.71	5.27	TGF-β Pseudoreceptor
NOTCH1	10.42	4.35	Core Notch Receptor
DLL1	4.61	4.1	Core Notch Ligand
RBPJ	4.01	5.99	Notch Core Mediator
ADAM17	2.88	11.88	Notch processing and activation
SKI	2.18	4.02	TGF-β Inhibitor

The module also contained intracellular signaling regulators, including DYRK2, TSC2, STK32B, ACTR1B, BCL2L1, HSF1, and ERN1. Several of these loci met Baseline Stable Treatment-Responsive criteria, while others were retained through the Dynamic Mover framework because treatment-associated methylation changes substantially exceeded baseline fluctuation despite greater pre-treatment variability. DYRK2 exhibited the largest treatment-associated methylation shift among loci within the module and was retained as a Dynamic Mover because its post-treatment change remained more than twice the magnitude of its baseline fluctuation.

Within the nucleus, the Notch-associated signaling cluster included RBPJ, SKI, NR5A2, and CRHBP. These genes are associated with transcriptional regulation, endocrine signaling, and cellular stress responses.

Together, these findings identified a Notch-associated signaling module comprising core pathway components and adjacent regulatory genes. Figure 4(A) illustrates the known biological relationships among these genes, while Figure 4(B) summarizes the magnitude and direction of treatment-associated methylation changes across representative loci within the module.

### Longitudinal methylation dynamics within the Notch module

Longitudinal analysis demonstrated that loci within the Notch-associated module exhibited treatment-associated methylation changes that exceeded baseline fluctuation. ACTR1B and retained loci mapped to SKI met Baseline Stable Treatment-Responsive criteria, indicating that their post-treatment movement was at least three times greater than pre-treatment fluctuation. DYRK2 exhibited the largest treatment-associated methylation change within the module but was retained as a Dynamic Mover because its post-treatment change was slightly more than twice the magnitude of its baseline fluctuation.

Figure 4(B) presents Δβ values comparing post-treatment methylation levels (T3) with the immediate pre-treatment assessment (T2). Methylation changes were bidirectional across the module, indicating that treatment-associated remodeling involved changes across multiple regulatory loci rather than uniform increases or decreases in methylation. Although effect sizes and baseline stability varied across loci, all loci shown met the magnitude and stability criteria required for retention in the final longitudinal methylation signature.

## Discussion

This study examined longitudinal DNA methylation changes associated with ketamine-assisted psychotherapy (KAP) in individuals with major depressive disorder using a within-subject design incorporating two pre-treatment baselines. Participants demonstrated marked reductions in depressive symptoms following treatment, while the dual-baseline design allowed post-treatment methylation change to be evaluated relative to pre-treatment group-level fluctuation. Within this framework, the observed post-treatment methylation changes exceeded the group-level fluctuation observed across the two pre-treatment assessments and are consistent with treatment-associated biological remodeling and align with emerging models proposing that ketamine creates a transient window of enhanced plasticity that may subsequently be stabilized through downstream regulatory processes [6].

A central finding of this study was the identification of 962 retained CpG loci following quantitative filtering for magnitude and baseline stability, of which 517 had available gene annotations and were included in biological interpretation. Rather than reflecting diffuse methylation drift, these gene-annotated loci were distributed across multiple biological domains, including transcriptional regulation, signaling integration, stress adaptation, structural remodeling, immune regulation, synaptic organization, and electrophysiological function. Together, these findings suggest that ketamine-associated epigenetic changes are organized and selective, consistent with emerging evidence that rapid-acting antidepressants engage downstream regulatory systems rather than producing widespread epigenomic disruption [7,8].

Exploratory enrichment analysis of the broader 603-locus set identified NOTCH1-related signaling terms, while the retained signature contained multiple interacting Notch components spanning ligand, receptor processing, transcriptional regulation, and pathway cross-talk. The Notch-associated module was therefore selected for focused, hypothesis-generating interpretation. The remaining retained loci were not assigned to formal functional categories in the present analysis.

Within the retained methylation signature, the Notch-associated signaling module was selected for detailed examination because it contained multiple components of canonical Notch signaling spanning receptor, ligand, transcriptional mediator, and regulatory interface elements within a single coordinated module. Exploratory g:Profiler analysis of the unique gene symbols mapped from all 603 gene-annotated magnitude-filtered loci identified two closely related Reactome terms concerning NOTCH1 signaling in the context of the NOTCH1 t(7;9) translocation mutant, both driven by overlap with NOTCH1, DLL1, and ADAM17. Because the enrichment input included 86 loci that did not meet the final stability criterion, these findings support representation of NOTCH1-related signaling machinery within the broader magnitude-filtered methylation set rather than enrichment of the final retained 517-locus signature. The enrichment remained exploratory and did not establish pathway activation, the presence of the translocation, or causality. Core Notch pathway members included NOTCH1, DLL1, and RBPJ, while adjacent regulatory components included ADAM17, BAMBI, and SKI. The coordinated representation of receptor, ligand, transcriptional mediator, and pathway regulators within the same treatment-associated methylation signature is notable given previous evidence linking Notch signaling to neurogenesis, synaptic plasticity, stress responsivity, and antidepressant response [15–17].

DYRK2 is a dual-specificity kinase involved in several regulatory processes relevant to cellular adaptation. Experimental studies have shown that DYRK2 can regulate NOTCH1 intracellular-domain stability through phosphorylation-dependent proteasomal degradation and can influence Hedgehog signaling, neuronal cytoskeletal organization, and process outgrowth [18,19]. Although these findings do not establish a role for DYRK2 in ketamine response or depression, they support its placement as an adjacent regulatory component within the exploratory Notch-associated module.

Alterations in Notch-related pathways have also been reported in both preclinical and human studies of mood disorders, supporting the biological plausibility of this observation [15–17]. Several genes within the Notch-associated module demonstrated particularly strong treatment-associated methylation movement relative to baseline fluctuation. DYRK2 exhibited one of the largest methylation shifts within the retained signature and was retained as a Dynamic Mover because its treatment-associated change was slightly more than twice the magnitude of its baseline fluctuation. Additional genes, including TSC2, ACTR1B, BCL2L1, HSF1, and ERN1, linked the module to intracellular signaling, stress adaptation, and cellular maintenance processes. Together, these observations suggest that ketamine-associated methylation remodeling may extend beyond individual signaling pathways to encompass broader regulatory systems involved in cellular adaptation. The prominence of DYRK2 within the retained signature is notable given prior evidence implicating this kinase in stress-responsive regulatory networks and broader cellular homeostatic processes [20].

The prominence of stress-related and endocrine-associated genes within the retained signature is also noteworthy. Genes involved in HPA-axis regulation, metabolic control, and cellular stress responses exhibited treatment-associated methylation changes, aligning with contemporary models proposing that successful antidepressant response involves coordinated normalization of stress-related biological systems alongside improvements in neuroplasticity [21,22]. Similar patterns have been reported in emerging studies of psychedelic-assisted therapies, raising the possibility that epigenetic remodeling of stress-responsive pathways may represent a broader feature of successful psychiatric intervention [21]. These findings can be situated within contemporary models of ketamine action. Current theories emphasize rapid glutamatergic signaling, synaptic potentiation, and transient enhancement of neuroplasticity following ketamine administration [2,23]. However, the biological processes supporting the persistence of these effects remain incompletely understood. Although the present findings do not establish a mechanism of antidepressant response, they do suggest that treatment-associated methylation remodeling may represent one component of the longer-term biological adaptations accompanying clinical improvement. Importantly, the identification of treatment-associated DNA methylation changes in peripheral tissues is consistent with an emerging literature supporting peripheral epigenetic signatures as candidate biomarkers in psychiatric interventions. However, peripheral methylation profiles should not be interpreted as direct proxies of brain-specific epigenetic states. Cross-tissue studies have demonstrated that DNA methylation correspondence between buccal and brain tissues is highly locus-dependent, with only a subset of CpG sites showing significant concordance across tissues [24]. Therefore, the loci identified in the present study should be interpreted as peripheral signatures associated with treatment-related biological adaptation rather than direct evidence of neuronal epigenetic remodeling or regulation of ketamine-responsive brain pathways.

A recent systematic review and meta-analysis examining non-pharmacological interventions across common mental disorders demonstrated that therapeutic interventions can be associated with measurable DNA methylation alterations in genes involved in stress regulation, immune processes, and neurobiological pathways, supporting the concept that peripheral epigenetic profiles may capture biologically relevant adaptations accompanying clinical improvement [25]. A recent longitudinal study examined OXT, SLC6A4, and NR3C1 methylation during depression treatment and found small changes over time in OXT and SLC6A4. These changes were not significantly associated with improvement in depressive symptoms and appeared to be influenced by cell-type composition, highlighting the need to account for potential confounders when interpreting peripheral methylation changes [26]. Together, these studies support the potential value of repeated peripheral methylation assessment for identifying systemic biological signatures associated with clinical response.

## Limitations

Several limitations should be acknowledged. First, the sample size was small (*n* = 10), limiting statistical power and generalizability. Nevertheless, the within-subject longitudinal design and use of two pre-treatment baselines allowed post-treatment methylation movement to be evaluated relative to pre-treatment fluctuation. The initial *p* < .05 threshold was used as a nominal exploratory screen rather than as evidence of epigenome-wide statistical significance, and the identified loci therefore require replication in larger independent cohorts. Second, DNA methylation was measured in buccal tissue rather than brain tissue. Tissue specificity remains an important limitation because methylation patterns are influenced by cellular composition and tissue-specific regulatory mechanisms [27]. Therefore, the identified loci should not be interpreted as direct measurements of neuronal epigenetic remodeling. However, accumulating evidence supports the utility of peripheral methylation profiles as accessible biomarkers of psychiatric treatment-associated biological changes. Recent systematic evidence indicates that therapeutic interventions can induce measurable peripheral DNA methylation alterations across stress-related, inflammatory, and neurobiological pathways [25]. These findings are consistent with recent longitudinal work showing that peripheral methylation changes during depression treatment may be small, unrelated to symptom improvement, and influenced by cell-type composition [26]. In addition, methylome-wide investigations of treatment response following electroconvulsive therapy have identified candidate peripheral methylation signatures associated with antidepressant outcomes, further supporting the feasibility of peripheral epigenetic biomarkers in treatment research [28]. Accordingly, the present findings should be interpreted as peripheral signatures of systemic regulatory adaptation associated with KAP rather than direct evidence of brain-specific molecular mechanisms. Third, DNA methylation changes cannot be assumed to reflect altered gene expression, protein abundance, or pathway activity. The observed methylation patterns therefore represent regulatory signatures associated with treatment rather than direct evidence of biological activation or inhibition. Finally, only a single post-treatment timepoint was available. As a result, it remains unclear whether the observed methylation changes persist, normalize, or continue to evolve over longer follow-up periods. Future studies incorporating larger samples, extended longitudinal follow-up, RNA expression data, and additional omics layers will be necessary to clarify the biological significance and temporal stability of these findings.

The present work focused on the Notch-associated signaling module because it provided the clearest mechanistic framework emerging from the longitudinal methylation signature. However, the retained set of 962 CpG loci, including 517 gene-annotated loci, spans numerous additional genes distributed across multiple biological domains. Future network-based analyses will investigate whether these genes form additional coordinated regulatory architectures that contribute to ketamine-associated epigenetic remodeling. Such analyses may reveal complementary signaling systems that interact with, or operate independently of, the Notch-centered module identified here.

## Conclusion

Ketamine-assisted psychotherapy was associated with significant clinical improvement and a coordinated pattern of longitudinal DNA methylation remodeling. Application of the quantitative filtering framework identified 962 retained CpG loci, including 517 retained gene-annotated loci involved in transcriptional regulation, signaling integration, stress adaptation, and neuroplasticity-related processes. Exploratory enrichment analysis of the broader set of 603 gene-annotated magnitude-filtered loci identified representation of NOTCH1-related signaling machinery. Within the retained signature, a Notch-associated module comprising NOTCH1, DLL1, RBPJ, ADAM17, BAMBI, SKI, and related regulatory genes emerged as a prominent biological feature. While causal inferences cannot be drawn from methylation data alone, these findings provide a foundation for future investigation into the regulatory systems associated with clinical response following ketamine-assisted psychotherapy.

## Supplementary Material

Supplementary.docx

Supplementary table 2.docx
