# Supplementary material for: Longitudinal peripheral DNA methylation remodeling following ketamine-assisted psychotherapy in major depressive disorder
Source: Epigenetics. 2026 Oct 15;21(1):2731659. doi: 10.1080/15592294.2026.2731659 (PMC13679325; doi:10.1080/15592294.2026.2731659)
Supplement: Supplementary file 1 [file KEPI_A_2731659_SM0051.docx]

**Table S1**: *Linear Mixed Model of Ketamine Effect on DNA Methylation*

**probe_id contrast estimate SE df t.ratio p.value**

cg25583367T1 - T2 0.013 0.008 22.222 1.713 0.223

cg25583367T1 - T3 -0.023 0.008 22.222 -2.907 0.021

cg25583367T2 - T3 -0.036 0.008 22.222 -4.620 0.000

cg17387838T1 - T2 0.016 0.010 22.222 1.629 0.255

cg17387838T1 - T3 -0.021 0.010 22.222 -2.148 0.103

cg17387838T2 - T3 -0.037 0.010 22.222 -3.778 0.003

cg16952841T1 - T2 -0.020 0.011 22.222 -1.784 0.198

cg16952841T1 - T3 -0.051 0.011 22.222 -4.611 0.000

cg16952841T2 - T3 -0.031 0.011 22.222 -2.827 0.025

cg01245880T1 - T2 0.029 0.012 22.222 2.401 0.063

cg01245880T1 - T3 -0.018 0.012 22.222 -1.500 0.310

cg01245880T2 - T3 -0.047 0.012 22.222 -3.902 0.002

cg19888870T1 - T2 0.022 0.010 22.222 2.081 0.117

cg19888870T1 - T3 -0.015 0.010 22.222 -1.462 0.328

cg19888870T2 - T3 -0.037 0.010 22.222 -3.543 0.005

cg12548176T1 - T2 0.001 0.013 22.222 0.068 0.997

cg12548176T1 - T3 -0.037 0.013 22.222 -2.898 0.022

cg12548176T2 - T3 -0.037 0.013 22.222 -2.966 0.019

cg08120141T1 - T2 -0.019 0.010 21.263 -1.836 0.182

cg08120141T1 - T3 0.016 0.011 21.988 1.532 0.296

cg08120141T2 - T3 0.035 0.011 21.988 3.309 0.009

cg12196692T1 - T2 0.013 0.010 22.222 1.273 0.424

cg12196692T1 - T3 -0.022 0.010 22.222 -2.144 0.104

cg12196692T2 - T3 -0.036 0.010 22.222 -3.418 0.007

cg20604286T1 - T2 -0.026 0.014 22.222 -1.938 0.152

cg20604286T1 - T3 0.008 0.014 22.222 0.586 0.829

cg20604286T2 - T3 0.034 0.014 22.222 2.524 0.049

cg24613789T1 - T2 -0.036 0.008 20.481 -4.643 0.000

cg24613789T1 - T3 0.001 0.008 20.806 0.072 0.997

cg24613789T2 - T3 0.037 0.008 20.481 4.718 0.000

cg02627657T1 - T2 -0.018 0.008 22.222 -2.180 0.097

cg02627657T1 - T3 0.015 0.008 22.222 1.866 0.172

cg02627657T2 - T3 0.033 0.008 22.222 4.045 0.001

cg22619018T1 - T2 0.009 0.012 22.222 0.713 0.758

cg22619018T1 - T3 0.040 0.012 22.222 3.265 0.009

cg22619018T2 - T3 0.031 0.012 22.222 2.551 0.046

cg18022964T1 - T2 0.048 0.010 22.222 4.927 0.000

cg18022964T1 - T3 0.012 0.010 22.222 1.248 0.438

cg18022964T2 - T3 -0.036 0.010 22.222 -3.679 0.004

cg22004227T1 - T2 0.000 0.009 22.222 0.050 0.999

cg22004227T1 - T3 -0.034 0.009 22.222 -3.900 0.002

cg22004227T2 - T3 -0.035 0.009 22.222 -3.950 0.002

cg01720220T1 - T2 0.007 0.008 22.222 0.893 0.650

cg01720220T1 - T3 -0.029 0.008 22.222 -3.437 0.006

cg01720220T2 - T3 -0.036 0.008 22.222 -4.330 0.001

cg11813694T1 - T2 0.017 0.007 22.222 2.547 0.046

cg11813694T1 - T3 -0.015 0.007 22.222 -2.237 0.087

cg11813694T2 - T3 -0.032 0.007 22.222 -4.784 0.000

cg12938338T1 - T2 -0.027 0.017 22.222 -1.604 0.265

cg12938338T1 - T3 0.015 0.017 22.222 0.884 0.656

cg12938338T2 - T3 0.042 0.017 22.222 2.488 0.052

cg05141815T1 - T2 0.003 0.016 22.222 0.207 0.977

cg05141815T1 - T3 -0.040 0.016 22.222 -2.527 0.048

cg05141815T2 - T3 -0.044 0.016 22.222 -2.735 0.031

cg25798605T1 - T2 -0.009 0.016 22.222 -0.550 0.848

cg25798605T1 - T3 -0.056 0.016 22.222 -3.524 0.005

cg25798605T2 - T3 -0.047 0.016 22.222 -2.974 0.018

cg01954686T1 - T2 -0.003 0.012 22.222 -0.238 0.969

cg01954686T1 - T3 -0.035 0.012 22.222 -3.027 0.016

cg01954686T2 - T3 -0.032 0.012 22.222 -2.789 0.028

cg14862455T1 - T2 0.006 0.018 22.222 0.354 0.934

cg14862455T1 - T3 -0.047 0.018 22.222 -2.636 0.038

cg14862455T2 - T3 -0.053 0.018 22.222 -2.989 0.018

cg18233649T1 - T2 0.035 0.013 22.222 2.722 0.032

cg18233649T1 - T3 -0.016 0.013 22.222 -1.234 0.447

cg18233649T2 - T3 -0.051 0.013 22.222 -3.956 0.002

cg09169375T1 - T2 0.015 0.006 22.222 2.255 0.084

cg09169375T1 - T3 -0.022 0.006 22.222 -3.481 0.006

cg09169375T2 - T3 -0.037 0.006 22.222 -5.735 0.000

cg11627808T1 - T2 0.000 0.011 22.222 0.037 0.999

cg11627808T1 - T3 -0.032 0.011 22.222 -2.794 0.027

cg11627808T2 - T3 -0.032 0.011 22.222 -2.831 0.025

cg24643429T1 - T2 0.010 0.007 22.222 1.419 0.349

cg24643429T1 - T3 -0.025 0.007 22.222 -3.690 0.003

cg24643429T2 - T3 -0.035 0.007 22.222 -5.109 0.000

cg04167585T1 - T2 0.013 0.009 22.222 1.473 0.322

cg04167585T1 - T3 -0.022 0.009 22.222 -2.614 0.040

cg04167585T2 - T3 -0.035 0.009 22.222 -4.087 0.001

cg22289662T1 - T2 0.021 0.014 22.222 1.555 0.286

cg22289662T1 - T3 -0.026 0.014 22.222 -1.933 0.153

cg22289662T2 - T3 -0.047 0.014 22.222 -3.487 0.006

cg00277753T1 - T2 0.006 0.012 22.222 0.458 0.891

cg00277753T1 - T3 -0.031 0.012 22.222 -2.526 0.048

cg00277753T2 - T3 -0.037 0.012 22.222 -2.984 0.018

cg00618078T1 - T2 -0.029 0.012 22.222 -2.333 0.072

cg00618078T1 - T3 0.007 0.012 22.222 0.549 0.848

cg00618078T2 - T3 0.035 0.012 22.222 2.883 0.022

cg05011266T1 - T2 0.024 0.010 22.222 2.504 0.051

cg05011266T1 - T3 -0.019 0.010 22.222 -1.976 0.142

cg05011266T2 - T3 -0.043 0.010 22.222 -4.481 0.001

cg21508623T1 - T2 0.034 0.024 22.222 1.387 0.365

cg21508623T1 - T3 -0.041 0.024 22.222 -1.666 0.240

cg21508623T2 - T3 -0.075 0.024 22.222 -3.053 0.015

cg01069707T1 - T2 -0.018 0.020 22.222 -0.919 0.634

cg01069707T1 - T3 -0.080 0.020 22.222 -4.033 0.002

cg01069707T2 - T3 -0.062 0.020 22.222 -3.115 0.013

cg26267333T1 - T2 0.013 0.009 20.613 1.436 0.341

cg26267333T1 - T3 -0.023 0.009 21.087 -2.384 0.066

cg26267333T2 - T3 -0.036 0.009 20.613 -3.912 0.002

cg10341513T1 - T2 0.035 0.009 22.222 4.110 0.001

cg10341513T1 - T3 -0.009 0.009 22.222 -1.090 0.530

cg10341513T2 - T3 -0.044 0.009 22.222 -5.199 0.000

cg00944846T1 - T2 0.014 0.009 22.222 1.680 0.235

cg00944846T1 - T3 -0.027 0.009 22.222 -3.145 0.012

cg00944846T2 - T3 -0.041 0.009 22.222 -4.824 0.000

cg09740598T1 - T2 0.014 0.009 22.222 1.555 0.285

cg09740598T1 - T3 -0.025 0.009 22.222 -2.862 0.024

cg09740598T2 - T3 -0.039 0.009 22.222 -4.417 0.001

cg21716443T1 - T2 0.022 0.014 22.222 1.558 0.284

cg21716443T1 - T3 -0.025 0.014 22.222 -1.761 0.206

cg21716443T2 - T3 -0.048 0.014 22.222 -3.320 0.008

cg06150437T1 - T2 0.013 0.009 22.222 1.520 0.301

cg06150437T1 - T3 -0.025 0.009 22.222 -2.895 0.022

cg06150437T2 - T3 -0.039 0.009 22.222 -4.414 0.001

cg04543014T1 - T2 -0.031 0.011 22.222 -2.862 0.024

cg04543014T1 - T3 0.006 0.011 22.222 0.558 0.843

cg04543014T2 - T3 0.037 0.011 22.222 3.421 0.007

cg00463449T1 - T2 0.007 0.011 22.222 0.613 0.815

cg00463449T1 - T3 -0.029 0.011 22.222 -2.723 0.032

cg00463449T2 - T3 -0.036 0.011 22.222 -3.336 0.008

cg18588298T1 - T2 0.023 0.009 22.222 2.415 0.061

cg18588298T1 - T3 -0.011 0.009 22.222 -1.202 0.464

cg18588298T2 - T3 -0.034 0.009 22.222 -3.618 0.004

cg07501539T1 - T2 -0.009 0.014 22.222 -0.613 0.814

cg07501539T1 - T3 -0.038 0.014 22.222 -2.637 0.038

cg07501539T2 - T3 -0.029 0.014 22.222 -2.024 0.130

cg27181306T1 - T2 -0.006 0.013 22.222 -0.448 0.896

cg27181306T1 - T3 -0.048 0.013 22.222 -3.723 0.003

cg27181306T2 - T3 -0.042 0.013 22.222 -3.276 0.009

cg05697870T1 - T2 -0.019 0.024 22.222 -0.779 0.720

cg05697870T1 - T3 0.042 0.024 22.222 1.756 0.208

cg05697870T2 - T3 0.061 0.024 22.222 2.535 0.048

cg22621695T1 - T2 0.230 0.129 22.222 1.787 0.197

cg22621695T1 - T3 -0.249 0.129 22.222 -1.933 0.153

cg22621695T2 - T3 -0.480 0.129 22.222 -3.720 0.003

cg18855621T1 - T2 0.023 0.010 22.222 2.172 0.098

cg18855621T1 - T3 -0.010 0.010 22.222 -0.925 0.631

cg18855621T2 - T3 -0.032 0.010 22.222 -3.097 0.014

cg12884495T1 - T2 -0.003 0.011 22.222 -0.309 0.949

cg12884495T1 - T3 0.026 0.011 22.222 2.385 0.065

cg12884495T2 - T3 0.029 0.011 22.222 2.694 0.034

cg13688262T1 - T2 0.001 0.010 22.222 0.141 0.989

cg13688262T1 - T3 -0.033 0.010 22.222 -3.436 0.006

cg13688262T2 - T3 -0.034 0.010 22.222 -3.577 0.005

cg07689123T1 - T2 0.016 0.010 22.222 1.594 0.269

cg07689123T1 - T3 -0.022 0.010 22.222 -2.113 0.110

cg07689123T2 - T3 -0.038 0.010 22.222 -3.707 0.003

cg22391424T1 - T2 0.026 0.013 22.222 1.996 0.137

cg22391424T1 - T3 -0.011 0.013 22.222 -0.885 0.655

cg22391424T2 - T3 -0.037 0.013 22.222 -2.881 0.023

cg04525189T1 - T2 -0.025 0.015 22.222 -1.654 0.245

cg04525189T1 - T3 0.029 0.015 22.222 1.956 0.147

cg04525189T2 - T3 0.054 0.015 22.222 3.611 0.004

cg20379239T1 - T2 -0.005 0.013 22.222 -0.349 0.935

cg20379239T1 - T3 -0.045 0.013 22.222 -3.467 0.006

cg20379239T2 - T3 -0.040 0.013 22.222 -3.119 0.013

cg09777386T1 - T2 -0.005 0.020 22.222 -0.237 0.970

cg09777386T1 - T3 -0.061 0.020 22.222 -3.071 0.015

cg09777386T2 - T3 -0.057 0.020 22.222 -2.834 0.025

cg01378812T1 - T2 0.009 0.014 22.222 0.618 0.812

cg01378812T1 - T3 -0.037 0.014 22.222 -2.566 0.044

cg01378812T2 - T3 -0.046 0.014 22.222 -3.185 0.011

cg10736914T1 - T2 -0.004 0.010 22.222 -0.409 0.912

cg10736914T1 - T3 -0.040 0.010 22.222 -4.168 0.001

cg10736914T2 - T3 -0.036 0.010 22.222 -3.759 0.003

cg11224904T1 - T2 0.006 0.019 22.222 0.294 0.953

cg11224904T1 - T3 0.080 0.019 22.222 4.205 0.001

cg11224904T2 - T3 0.075 0.019 22.222 3.910 0.002

cg26981651T1 - T2 -0.011 0.008 22.222 -1.315 0.402

cg26981651T1 - T3 -0.032 0.008 22.222 -3.791 0.003

cg26981651T2 - T3 -0.021 0.008 22.222 -2.476 0.054

cg04438162T1 - T2 0.023 0.013 22.222 1.774 0.201

cg04438162T1 - T3 -0.015 0.013 22.222 -1.161 0.488

cg04438162T2 - T3 -0.039 0.013 22.222 -2.934 0.020

cg22588813T1 - T2 -0.007 0.014 22.222 -0.480 0.881

cg22588813T1 - T3 -0.054 0.014 22.222 -3.860 0.002

cg22588813T2 - T3 -0.048 0.014 22.222 -3.380 0.007

cg06524509T1 - T2 -0.007 0.021 22.222 -0.319 0.946

cg06524509T1 - T3 -0.072 0.021 22.222 -3.498 0.005

cg06524509T2 - T3 -0.066 0.021 22.222 -3.180 0.011

cg08523384T1 - T2 -0.017 0.013 22.222 -1.348 0.384

cg08523384T1 - T3 0.034 0.013 22.222 2.685 0.035

cg08523384T2 - T3 0.051 0.013 22.222 4.033 0.002

cg00997647T1 - T2 0.029 0.015 22.222 1.982 0.140

cg00997647T1 - T3 -0.019 0.015 22.222 -1.255 0.434

cg00997647T2 - T3 -0.048 0.015 22.222 -3.237 0.010

cg18216386T1 - T2 0.012 0.013 22.222 0.921 0.633

cg18216386T1 - T3 -0.042 0.013 22.222 -3.118 0.013

cg18216386T2 - T3 -0.054 0.013 22.222 -4.039 0.002

cg22359668T1 - T2 0.010 0.013 22.222 0.784 0.716

cg22359668T1 - T3 -0.036 0.013 22.222 -2.822 0.026

cg22359668T2 - T3 -0.046 0.013 22.222 -3.606 0.004

cg00040426T1 - T2 0.002 0.010 22.222 0.213 0.975

cg00040426T1 - T3 -0.033 0.010 22.222 -3.343 0.008

cg00040426T2 - T3 -0.035 0.010 22.222 -3.557 0.005

cg03213310T1 - T2 -0.029 0.015 22.222 -2.004 0.135

cg03213310T1 - T3 0.006 0.015 22.222 0.448 0.896

cg03213310T2 - T3 0.036 0.015 22.222 2.452 0.056

cg09689461T1 - T2 -0.002 0.021 22.222 -0.114 0.993

cg09689461T1 - T3 -0.059 0.021 22.222 -2.836 0.025

cg09689461T2 - T3 -0.056 0.021 22.222 -2.721 0.032

cg07078329T1 - T2 -0.031 0.029 22.222 -1.069 0.542

cg07078329T1 - T3 -0.119 0.029 22.222 -4.160 0.001

cg07078329T2 - T3 -0.089 0.029 22.222 -3.090 0.014

cg16328851T1 - T2 0.011 0.009 22.222 1.147 0.496

cg16328851T1 - T3 -0.019 0.009 22.222 -2.028 0.129

cg16328851T2 - T3 -0.030 0.009 22.222 -3.175 0.012

cg25967669T1 - T2 0.006 0.009 22.222 0.621 0.810

cg25967669T1 - T3 -0.032 0.009 22.222 -3.462 0.006

cg25967669T2 - T3 -0.038 0.009 22.222 -4.083 0.001

cg07340708T1 - T2 0.018 0.015 22.222 1.189 0.472

cg07340708T1 - T3 -0.033 0.015 22.222 -2.189 0.095

cg07340708T2 - T3 -0.051 0.015 22.222 -3.378 0.007

cg24131671T1 - T2 0.007 0.024 22.222 0.313 0.948

cg24131671T1 - T3 -0.078 0.024 22.222 -3.295 0.009

cg24131671T2 - T3 -0.085 0.024 22.222 -3.608 0.004

cg01808508T1 - T2 0.023 0.022 21.221 1.059 0.549

cg01808508T1 - T3 -0.062 0.023 21.566 -2.703 0.034

cg01808508T2 - T3 -0.086 0.023 21.566 -3.723 0.003

cg13290331T1 - T2 -0.003 0.013 22.222 -0.205 0.977

cg13290331T1 - T3 -0.028 0.013 22.222 -2.106 0.112

cg13290331T2 - T3 -0.025 0.013 22.222 -1.901 0.162

cg10772322T1 - T2 0.026 0.017 22.222 1.585 0.273

cg10772322T1 - T3 -0.038 0.017 22.222 -2.293 0.078

cg10772322T2 - T3 -0.065 0.017 22.222 -3.877 0.002

cg18778097T1 - T2 0.012 0.010 22.222 1.190 0.471

cg18778097T1 - T3 -0.028 0.010 22.222 -2.850 0.024

cg18778097T2 - T3 -0.039 0.010 22.222 -4.040 0.002

cg16995750T1 - T2 0.005 0.006 22.222 0.815 0.698

cg16995750T1 - T3 -0.028 0.006 22.222 -4.401 0.001

cg16995750T2 - T3 -0.034 0.006 22.222 -5.216 0.000

cg18379295T1 - T2 -0.014 0.016 22.222 -0.847 0.678

cg18379295T1 - T3 0.047 0.016 22.222 2.971 0.018

cg18379295T2 - T3 0.061 0.016 22.222 3.818 0.003

cg26249135T1 - T2 0.038 0.013 22.222 2.815 0.026

cg26249135T1 - T3 -0.016 0.013 22.222 -1.163 0.487

cg26249135T2 - T3 -0.053 0.013 22.222 -3.977 0.002

cg17376299T1 - T2 0.014 0.014 22.222 1.017 0.574

cg17376299T1 - T3 -0.031 0.014 22.222 -2.202 0.093

cg17376299T2 - T3 -0.045 0.014 22.222 -3.218 0.011

cg02071454T1 - T2 -0.002 0.018 22.222 -0.120 0.992

cg02071454T1 - T3 -0.040 0.018 22.222 -2.165 0.100

cg02071454T2 - T3 -0.038 0.018 22.222 -2.046 0.125

cg05343512T1 - T2 0.020 0.014 22.222 1.436 0.340

cg05343512T1 - T3 -0.027 0.014 22.222 -1.970 0.143

cg05343512T2 - T3 -0.046 0.014 22.222 -3.406 0.007

cg01023504T1 - T2 0.006 0.010 22.222 0.617 0.812

cg01023504T1 - T3 -0.029 0.010 22.222 -2.928 0.020

cg01023504T2 - T3 -0.035 0.010 22.222 -3.546 0.005

cg23993367T1 - T2 0.019 0.016 22.222 1.176 0.479

cg23993367T1 - T3 -0.029 0.016 22.222 -1.769 0.203

cg23993367T2 - T3 -0.048 0.016 22.222 -2.945 0.020

cg09076022T1 - T2 0.011 0.015 22.222 0.771 0.725

cg09076022T1 - T3 -0.043 0.015 22.222 -2.908 0.021

cg09076022T2 - T3 -0.055 0.015 22.222 -3.679 0.004

cg16544010T1 - T2 0.014 0.017 22.222 0.830 0.689

cg16544010T1 - T3 -0.029 0.017 22.222 -1.739 0.213

cg16544010T2 - T3 -0.043 0.017 22.222 -2.568 0.044

cg12405432T1 - T2 -0.013 0.013 22.222 -1.012 0.577

cg12405432T1 - T3 0.022 0.013 22.222 1.744 0.211

cg12405432T2 - T3 0.034 0.013 22.222 2.756 0.030

cg13320683T1 - T2 -0.010 0.010 22.222 -0.977 0.599

cg13320683T1 - T3 0.030 0.010 22.222 3.015 0.017

cg13320683T2 - T3 0.040 0.010 22.222 3.992 0.002

cg18758031T1 - T2 0.015 0.017 22.222 0.900 0.646

cg18758031T1 - T3 -0.049 0.017 22.222 -2.978 0.018

cg18758031T2 - T3 -0.064 0.017 22.222 -3.878 0.002

cg04330057T1 - T2 0.029 0.012 22.222 2.327 0.073

cg04330057T1 - T3 -0.007 0.012 22.222 -0.600 0.822

cg04330057T2 - T3 -0.036 0.012 22.222 -2.927 0.020

cg25792718T1 - T2 0.029 0.012 22.222 2.351 0.069

cg25792718T1 - T3 -0.016 0.012 22.222 -1.296 0.412

cg25792718T2 - T3 -0.045 0.012 22.222 -3.648 0.004

cg23902821T1 - T2 -0.001 0.022 22.222 -0.066 0.998

cg23902821T1 - T3 0.059 0.022 22.222 2.683 0.035

cg23902821T2 - T3 0.060 0.022 22.222 2.749 0.030

cg04317195T1 - T2 0.014 0.011 22.222 1.285 0.418

cg04317195T1 - T3 -0.021 0.011 22.222 -1.853 0.176

cg04317195T2 - T3 -0.035 0.011 22.222 -3.138 0.013

cg00297702T1 - T2 0.027 0.015 22.222 1.787 0.197

cg00297702T1 - T3 -0.013 0.015 22.222 -0.828 0.690

cg00297702T2 - T3 -0.040 0.015 22.222 -2.615 0.040

cg16487861T1 - T2 0.011 0.010 22.222 1.139 0.501

cg16487861T1 - T3 -0.026 0.010 22.222 -2.777 0.028

cg16487861T2 - T3 -0.037 0.010 22.222 -3.916 0.002

cg00723017T1 - T2 0.003 0.013 22.222 0.259 0.964

cg00723017T1 - T3 -0.033 0.013 22.222 -2.497 0.051

cg00723017T2 - T3 -0.037 0.013 22.222 -2.757 0.030

cg23482132T1 - T2 -0.013 0.013 22.222 -1.015 0.575

cg23482132T1 - T3 0.026 0.013 22.222 1.967 0.144

cg23482132T2 - T3 0.040 0.013 22.222 2.982 0.018

cg05256228T1 - T2 0.021 0.011 22.222 1.814 0.188

cg05256228T1 - T3 -0.025 0.011 22.222 -2.191 0.095

cg05256228T2 - T3 -0.046 0.011 22.222 -4.005 0.002

cg05197360T1 - T2 0.015 0.012 22.222 1.236 0.445

cg05197360T1 - T3 -0.021 0.012 22.222 -1.668 0.239

cg05197360T2 - T3 -0.036 0.012 22.222 -2.904 0.021

cg11191979T1 - T2 0.008 0.013 20.786 0.603 0.820

cg11191979T1 - T3 -0.046 0.013 20.271 -3.631 0.004

cg11191979T2 - T3 -0.054 0.013 20.786 -4.250 0.001

cg06193232T1 - T2 -0.018 0.014 22.222 -1.277 0.422

cg06193232T1 - T3 0.019 0.014 22.222 1.311 0.404

cg06193232T2 - T3 0.037 0.014 22.222 2.588 0.043

cg09147065T1 - T2 0.027 0.029 22.222 0.921 0.633

cg09147065T1 - T3 -0.033 0.029 22.222 -1.149 0.495

cg09147065T2 - T3 -0.060 0.029 22.222 -2.070 0.119

cg00987494T1 - T2 0.015 0.013 22.222 1.177 0.479

cg00987494T1 - T3 -0.028 0.013 22.222 -2.202 0.093

cg00987494T2 - T3 -0.042 0.013 22.222 -3.380 0.007

cg19940563T1 - T2 0.018 0.013 22.222 1.398 0.359

cg19940563T1 - T3 -0.035 0.013 22.222 -2.681 0.035

cg19940563T2 - T3 -0.053 0.013 22.222 -4.080 0.001

cg22575656T1 - T2 0.010 0.011 22.222 0.892 0.651

cg22575656T1 - T3 -0.028 0.011 22.222 -2.570 0.044

cg22575656T2 - T3 -0.037 0.011 22.222 -3.462 0.006

cg04460931T1 - T2 -0.003 0.011 22.222 -0.247 0.967

cg04460931T1 - T3 -0.032 0.011 22.222 -3.028 0.016

cg04460931T2 - T3 -0.030 0.011 22.222 -2.781 0.028

cg26962439T1 - T2 -0.014 0.014 22.222 -1.019 0.573

cg26962439T1 - T3 0.031 0.014 22.222 2.245 0.085

cg26962439T2 - T3 0.045 0.014 22.222 3.264 0.009

cg25725533T1 - T2 -0.049 0.052 22.222 -0.938 0.622

cg25725533T1 - T3 0.123 0.052 22.222 2.343 0.070

cg25725533T2 - T3 0.172 0.052 22.222 3.281 0.009

cg08684893T1 - T2 0.029 0.029 21.721 0.993 0.589

cg08684893T1 - T3 -0.042 0.029 21.721 -1.453 0.333

cg08684893T2 - T3 -0.070 0.028 21.225 -2.536 0.048

cg04694205T1 - T2 0.007 0.008 22.222 0.900 0.646

cg04694205T1 - T3 -0.030 0.008 22.222 -3.724 0.003

cg04694205T2 - T3 -0.037 0.008 22.222 -4.624 0.000

cg02630352T1 - T2 0.006 0.017 22.222 0.348 0.936

cg02630352T1 - T3 -0.048 0.017 22.222 -2.908 0.021

cg02630352T2 - T3 -0.054 0.017 22.222 -3.256 0.010

cg05494436T1 - T2 0.015 0.012 22.222 1.208 0.461

cg05494436T1 - T3 -0.028 0.012 22.222 -2.341 0.071

cg05494436T2 - T3 -0.043 0.012 22.222 -3.549 0.005

cg15793218T1 - T2 0.033 0.014 22.222 2.323 0.073

cg15793218T1 - T3 0.002 0.014 22.222 0.118 0.992

cg15793218T2 - T3 -0.032 0.014 22.222 -2.205 0.092

cg25639809T1 - T2 0.006 0.011 22.222 0.541 0.852

cg25639809T1 - T3 -0.032 0.011 22.222 -3.069 0.015

cg25639809T2 - T3 -0.038 0.011 22.222 -3.610 0.004

cg01683215T1 - T2 0.034 0.021 22.222 1.605 0.264

cg01683215T1 - T3 -0.040 0.021 22.222 -1.921 0.156

cg01683215T2 - T3 -0.074 0.021 22.222 -3.526 0.005

cg16611852T1 - T2 -0.003 0.014 22.222 -0.183 0.982

cg16611852T1 - T3 0.036 0.014 22.222 2.489 0.052

cg16611852T2 - T3 0.038 0.014 22.222 2.673 0.036

cg17219201T1 - T2 0.009 0.025 22.222 0.349 0.935

cg17219201T1 - T3 -0.051 0.025 22.222 -2.057 0.122

cg17219201T2 - T3 -0.060 0.025 22.222 -2.406 0.062

cg15767067T1 - T2 -0.022 0.011 22.222 -1.949 0.149

cg15767067T1 - T3 0.013 0.011 22.222 1.154 0.492

cg15767067T2 - T3 0.035 0.011 22.222 3.104 0.014

cg25157850T1 - T2 0.026 0.016 22.222 1.668 0.239

cg25157850T1 - T3 -0.031 0.016 22.222 -1.976 0.142

cg25157850T2 - T3 -0.057 0.016 22.222 -3.644 0.004

cg00606392T1 - T2 0.017 0.010 22.222 1.745 0.211

cg00606392T1 - T3 -0.021 0.010 22.222 -2.122 0.108

cg00606392T2 - T3 -0.038 0.010 22.222 -3.866 0.002

cg18615431T1 - T2 -0.015 0.016 22.222 -0.935 0.624

cg18615431T1 - T3 0.032 0.016 22.222 2.035 0.127

cg18615431T2 - T3 0.046 0.016 22.222 2.970 0.019

cg16167452T1 - T2 -0.008 0.014 22.222 -0.593 0.825

cg16167452T1 - T3 0.039 0.014 22.222 2.863 0.023

cg16167452T2 - T3 0.047 0.014 22.222 3.456 0.006

cg02246193T1 - T2 -0.051 0.024 22.222 -2.132 0.106

cg02246193T1 - T3 0.014 0.024 22.222 0.583 0.831

cg02246193T2 - T3 0.065 0.024 22.222 2.715 0.032

cg12449049T1 - T2 -0.014 0.018 22.222 -0.760 0.731

cg12449049T1 - T3 0.035 0.018 22.222 1.961 0.145

cg12449049T2 - T3 0.049 0.018 22.222 2.721 0.032

cg06814146T1 - T2 -0.016 0.017 22.222 -0.986 0.593

cg06814146T1 - T3 0.016 0.017 22.222 0.999 0.585

cg06814146T2 - T3 0.033 0.017 22.222 1.985 0.139

cg03820499T1 - T2 0.020 0.018 22.222 1.146 0.497

cg03820499T1 - T3 -0.039 0.018 22.222 -2.158 0.101

cg03820499T2 - T3 -0.059 0.018 22.222 -3.304 0.009

cg07590058T1 - T2 0.028 0.017 22.222 1.670 0.239

cg07590058T1 - T3 -0.019 0.017 22.222 -1.163 0.487

cg07590058T2 - T3 -0.047 0.017 22.222 -2.833 0.025

cg14530169T1 - T2 0.008 0.019 22.222 0.406 0.913

cg14530169T1 - T3 -0.041 0.019 22.222 -2.141 0.104

cg14530169T2 - T3 -0.049 0.019 22.222 -2.547 0.046

cg13732201T1 - T2 0.011 0.015 22.222 0.722 0.753

cg13732201T1 - T3 -0.034 0.015 22.222 -2.325 0.073

cg13732201T2 - T3 -0.044 0.015 22.222 -3.047 0.016

cg01984991T1 - T2 0.013 0.014 22.222 0.895 0.649

cg01984991T1 - T3 -0.042 0.014 22.222 -2.940 0.020

cg01984991T2 - T3 -0.055 0.014 22.222 -3.835 0.002

cg08376992T1 - T2 -0.010 0.013 22.222 -0.722 0.753

cg08376992T1 - T3 0.026 0.013 22.222 1.907 0.160

cg08376992T2 - T3 0.035 0.013 22.222 2.628 0.039

cg24349665T1 - T2 0.005 0.020 22.222 0.265 0.962

cg24349665T1 - T3 -0.067 0.020 22.222 -3.393 0.007

cg24349665T2 - T3 -0.072 0.020 22.222 -3.658 0.004

cg06052323T1 - T2 0.003 0.020 22.222 0.168 0.985

cg06052323T1 - T3 -0.039 0.020 22.222 -1.937 0.152

cg06052323T2 - T3 -0.042 0.020 22.222 -2.106 0.112

cg20083950T1 - T2 0.017 0.010 22.222 1.728 0.217

cg20083950T1 - T3 -0.021 0.010 22.222 -2.193 0.094

cg20083950T2 - T3 -0.038 0.010 22.222 -3.921 0.002

cg10372493T1 - T2 -0.001 0.016 22.222 -0.070 0.997

cg10372493T1 - T3 -0.049 0.016 22.222 -3.107 0.014

cg10372493T2 - T3 -0.048 0.016 22.222 -3.037 0.016

cg16342993T1 - T2 0.029 0.016 22.054 1.869 0.171

cg16342993T1 - T3 -0.004 0.015 21.280 -0.242 0.968

cg16342993T2 - T3 -0.033 0.016 22.054 -2.103 0.112

cg19347582T1 - T2 0.038 0.025 22.222 1.486 0.317

cg19347582T1 - T3 -0.022 0.025 22.222 -0.853 0.675

cg19347582T2 - T3 -0.060 0.025 22.222 -2.339 0.071

cg19268388T1 - T2 0.014 0.009 22.222 1.500 0.310

cg19268388T1 - T3 -0.025 0.009 22.222 -2.737 0.031

cg19268388T2 - T3 -0.039 0.009 22.222 -4.237 0.001

cg03029232T1 - T2 -0.021 0.016 22.222 -1.334 0.392

cg03029232T1 - T3 0.017 0.016 22.222 1.071 0.541

cg03029232T2 - T3 0.037 0.016 22.222 2.405 0.062

cg03979754T1 - T2 -0.021 0.010 22.222 -2.045 0.125

cg03979754T1 - T3 0.020 0.010 22.222 1.926 0.155

cg03979754T2 - T3 0.041 0.010 22.222 3.971 0.002

cg24180369T1 - T2 0.014 0.010 22.222 1.450 0.333

cg24180369T1 - T3 -0.024 0.010 22.222 -2.429 0.059

cg24180369T2 - T3 -0.039 0.010 22.222 -3.879 0.002

cg02129737T1 - T2 0.008 0.013 22.222 0.598 0.822

cg02129737T1 - T3 -0.034 0.013 22.222 -2.642 0.038

cg02129737T2 - T3 -0.042 0.013 22.222 -3.240 0.010

cg10259701T1 - T2 -0.009 0.016 22.222 -0.599 0.822

cg10259701T1 - T3 0.024 0.016 22.222 1.531 0.296

cg10259701T2 - T3 0.033 0.016 22.222 2.130 0.107

cg24720361T1 - T2 -0.013 0.014 20.620 -0.924 0.632

cg24720361T1 - T3 -0.036 0.013 21.370 -2.744 0.031

cg24720361T2 - T3 -0.024 0.013 21.370 -1.798 0.194

cg18119211T1 - T2 0.009 0.017 22.222 0.514 0.865

cg18119211T1 - T3 -0.040 0.017 22.222 -2.313 0.075

cg18119211T2 - T3 -0.049 0.017 22.222 -2.828 0.025

cg26791399T1 - T2 0.007 0.016 22.222 0.419 0.908

cg26791399T1 - T3 0.040 0.016 22.222 2.496 0.051

cg26791399T2 - T3 0.033 0.016 22.222 2.078 0.118

cg15342346T1 - T2 -0.008 0.011 22.222 -0.704 0.764

cg15342346T1 - T3 0.031 0.011 22.222 2.808 0.026

cg15342346T2 - T3 0.038 0.011 22.222 3.512 0.005

cg08466983T1 - T2 -0.006 0.013 22.222 -0.468 0.887

cg08466983T1 - T3 0.039 0.013 22.222 3.113 0.013

cg08466983T2 - T3 0.045 0.013 22.222 3.581 0.005

cg05184570T1 - T2 -0.006 0.018 22.222 -0.339 0.939

cg05184570T1 - T3 -0.064 0.018 22.222 -3.650 0.004

cg05184570T2 - T3 -0.058 0.018 22.222 -3.311 0.008

cg11367593T1 - T2 -0.030 0.013 22.222 -2.412 0.061

cg11367593T1 - T3 0.015 0.013 22.222 1.214 0.458

cg11367593T2 - T3 0.046 0.013 22.222 3.625 0.004

cg26792288T1 - T2 0.014 0.020 22.222 0.694 0.769

cg26792288T1 - T3 -0.038 0.020 22.222 -1.889 0.165

cg26792288T2 - T3 -0.053 0.020 22.222 -2.584 0.043

cg18761499T1 - T2 0.012 0.013 22.222 0.956 0.611

cg18761499T1 - T3 -0.038 0.013 22.222 -2.920 0.021

cg18761499T2 - T3 -0.050 0.013 22.222 -3.877 0.002

cg25439420T1 - T2 0.004 0.017 22.222 0.229 0.971

cg25439420T1 - T3 0.051 0.017 22.222 2.975 0.018

cg25439420T2 - T3 0.047 0.017 22.222 2.746 0.030

cg15097361T1 - T2 -0.017 0.013 22.222 -1.320 0.399

cg15097361T1 - T3 0.025 0.013 22.222 1.930 0.154

cg15097361T2 - T3 0.042 0.013 22.222 3.250 0.010

cg07474379T1 - T2 -0.024 0.016 22.054 -1.510 0.306

cg07474379T1 - T3 0.008 0.016 22.054 0.522 0.862

cg07474379T2 - T3 0.033 0.016 21.280 2.096 0.115

cg03408433T1 - T2 -0.019 0.012 22.222 -1.531 0.296

cg03408433T1 - T3 0.017 0.012 22.222 1.396 0.360

cg03408433T2 - T3 0.036 0.012 22.222 2.926 0.020

cg03710955T1 - T2 0.001 0.009 22.222 0.141 0.989

cg03710955T1 - T3 -0.033 0.009 22.222 -3.732 0.003

cg03710955T2 - T3 -0.034 0.009 22.222 -3.873 0.002

cg10955576T1 - T2 0.016 0.010 22.222 1.652 0.246

cg10955576T1 - T3 -0.024 0.010 22.222 -2.496 0.052

cg10955576T2 - T3 -0.039 0.010 22.222 -4.147 0.001

cg08884591T1 - T2 0.021 0.030 22.222 0.681 0.777

cg08884591T1 - T3 -0.080 0.030 22.222 -2.649 0.037

cg08884591T2 - T3 -0.100 0.030 22.222 -3.330 0.008

cg11713658T1 - T2 -0.030 0.021 22.222 -1.421 0.347

cg11713658T1 - T3 0.050 0.021 22.222 2.382 0.065

cg11713658T2 - T3 0.080 0.021 22.222 3.803 0.003

cg20752671T1 - T2 0.008 0.016 20.693 0.479 0.882

cg20752671T1 - T3 -0.037 0.017 21.245 -2.200 0.094

cg20752671T2 - T3 -0.045 0.016 20.693 -2.762 0.030

cg02305723T1 - T2 0.040 0.015 22.222 2.717 0.032

cg02305723T1 - T3 -0.009 0.015 22.222 -0.635 0.803

cg02305723T2 - T3 -0.050 0.015 22.222 -3.352 0.008

cg15822108T1 - T2 -0.020 0.013 22.222 -1.490 0.315

cg15822108T1 - T3 0.013 0.013 22.222 0.968 0.604

cg15822108T2 - T3 0.032 0.013 22.222 2.458 0.056

cg16831305T1 - T2 0.003 0.012 22.222 0.277 0.959

cg16831305T1 - T3 -0.033 0.012 22.222 -2.828 0.025

cg16831305T2 - T3 -0.036 0.012 22.222 -3.105 0.014

cg08572213T1 - T2 0.012 0.012 22.222 1.027 0.568

cg08572213T1 - T3 -0.022 0.012 22.222 -1.858 0.174

cg08572213T2 - T3 -0.034 0.012 22.222 -2.885 0.022

cg16591226T1 - T2 0.014 0.014 22.222 0.997 0.586

cg16591226T1 - T3 -0.030 0.014 22.222 -2.081 0.117

cg16591226T2 - T3 -0.044 0.014 22.222 -3.078 0.014

cg18572150T1 - T2 0.025 0.021 22.222 1.171 0.482

cg18572150T1 - T3 -0.030 0.021 22.222 -1.424 0.346

cg18572150T2 - T3 -0.054 0.021 22.222 -2.595 0.042

cg25764700T1 - T2 -0.012 0.012 22.222 -0.989 0.591

cg25764700T1 - T3 0.024 0.012 22.222 2.015 0.132

cg25764700T2 - T3 0.035 0.012 22.222 3.005 0.017

cg18622506T1 - T2 -0.026 0.020 22.222 -1.272 0.425

cg18622506T1 - T3 0.013 0.020 22.222 0.637 0.802

cg18622506T2 - T3 0.039 0.020 22.222 1.909 0.160

cg03734391T1 - T2 -0.012 0.011 22.222 -1.066 0.545

cg03734391T1 - T3 0.024 0.011 22.222 2.212 0.091

cg03734391T2 - T3 0.036 0.011 22.222 3.278 0.009

cg03942086T1 - T2 0.009 0.012 22.222 0.786 0.715

cg03942086T1 - T3 -0.036 0.012 22.222 -3.016 0.017

cg03942086T2 - T3 -0.045 0.012 22.222 -3.803 0.003

cg24006654T1 - T2 -0.017 0.019 22.222 -0.873 0.663

cg24006654T1 - T3 0.027 0.019 22.222 1.410 0.353

cg24006654T2 - T3 0.043 0.019 22.222 2.283 0.079

cg21854271T1 - T2 0.012 0.032 22.222 0.360 0.931

cg21854271T1 - T3 -0.086 0.032 22.222 -2.660 0.036

cg21854271T2 - T3 -0.098 0.032 22.222 -3.019 0.017

cg04487659T1 - T2 0.013 0.010 22.222 1.216 0.457

cg04487659T1 - T3 0.037 0.010 22.222 3.494 0.006

cg04487659T2 - T3 0.024 0.010 22.222 2.278 0.080

cg08906233T1 - T2 -0.032 0.018 22.222 -1.712 0.223

cg08906233T1 - T3 0.015 0.018 22.222 0.818 0.696

cg08906233T2 - T3 0.047 0.018 22.222 2.530 0.048

cg10533255T1 - T2 0.016 0.009 22.222 1.710 0.224

cg10533255T1 - T3 -0.019 0.009 22.222 -2.034 0.127

cg10533255T2 - T3 -0.035 0.009 22.222 -3.744 0.003

cg26593521T1 - T2 0.006 0.010 22.222 0.567 0.839

cg26593521T1 - T3 -0.027 0.010 22.222 -2.730 0.031

cg26593521T2 - T3 -0.033 0.010 22.222 -3.297 0.009

cg02008651T1 - T2 -0.015 0.016 20.661 -0.903 0.644

cg02008651T1 - T3 0.045 0.016 20.661 2.760 0.030

cg02008651T2 - T3 0.060 0.017 21.195 3.530 0.005

cg02314586T1 - T2 -0.020 0.017 22.222 -1.154 0.492

cg02314586T1 - T3 0.015 0.017 22.222 0.902 0.645

cg02314586T2 - T3 0.035 0.017 22.222 2.057 0.122

cg12663665T1 - T2 -0.012 0.010 22.222 -1.221 0.454

cg12663665T1 - T3 0.023 0.010 22.222 2.245 0.085

cg12663665T2 - T3 0.035 0.010 22.222 3.466 0.006

cg17790171T1 - T2 0.023 0.013 22.222 1.738 0.214

cg17790171T1 - T3 -0.021 0.013 22.222 -1.624 0.257

cg17790171T2 - T3 -0.044 0.013 22.222 -3.362 0.008

cg20294962T1 - T2 0.013 0.010 22.222 1.320 0.399

cg20294962T1 - T3 -0.025 0.010 22.222 -2.496 0.051

cg20294962T2 - T3 -0.038 0.010 22.222 -3.816 0.003

cg19470987T1 - T2 0.009 0.016 22.222 0.531 0.857

cg19470987T1 - T3 -0.042 0.016 22.222 -2.596 0.042

cg19470987T2 - T3 -0.051 0.016 22.222 -3.127 0.013

cg03486475T1 - T2 0.029 0.024 22.222 1.214 0.458

cg03486475T1 - T3 -0.054 0.024 22.222 -2.252 0.084

cg03486475T2 - T3 -0.083 0.024 22.222 -3.466 0.006

cg01653686T1 - T2 0.023 0.011 22.222 2.066 0.120

cg01653686T1 - T3 -0.011 0.011 22.222 -0.998 0.586

cg01653686T2 - T3 -0.035 0.011 22.222 -3.064 0.015

cg02433509T1 - T2 0.009 0.019 22.222 0.469 0.886

cg02433509T1 - T3 -0.033 0.019 22.222 -1.806 0.191

cg02433509T2 - T3 -0.042 0.019 22.222 -2.275 0.081

cg05952897T1 - T2 0.026 0.019 22.222 1.325 0.396

cg05952897T1 - T3 -0.033 0.019 22.222 -1.726 0.218

cg05952897T2 - T3 -0.059 0.019 22.222 -3.051 0.015

cg04749882T1 - T2 0.014 0.011 22.222 1.301 0.409

cg04749882T1 - T3 -0.022 0.011 22.222 -2.004 0.135

cg04749882T2 - T3 -0.036 0.011 22.222 -3.305 0.009

cg13588388T1 - T2 -0.011 0.015 22.222 -0.684 0.775

cg13588388T1 - T3 0.041 0.015 22.222 2.622 0.040

cg13588388T2 - T3 0.051 0.015 22.222 3.306 0.009

cg21566370T1 - T2 -0.044 0.031 22.222 -1.399 0.359

cg21566370T1 - T3 0.037 0.031 22.222 1.185 0.474

cg21566370T2 - T3 0.081 0.031 22.222 2.584 0.043

cg07333573T1 - T2 0.012 0.008 22.222 1.448 0.334

cg07333573T1 - T3 -0.027 0.008 22.222 -3.290 0.009

cg07333573T2 - T3 -0.039 0.008 22.222 -4.738 0.000

cg18048620T1 - T2 -0.019 0.014 22.222 -1.343 0.387

cg18048620T1 - T3 0.029 0.014 22.222 2.081 0.117

cg18048620T2 - T3 0.047 0.014 22.222 3.423 0.007

cg25758195T1 - T2 0.015 0.011 22.222 1.419 0.349

cg25758195T1 - T3 -0.016 0.011 22.222 -1.521 0.301

cg25758195T2 - T3 -0.032 0.011 22.222 -2.939 0.020

cg01288904T1 - T2 0.004 0.016 22.222 0.249 0.966

cg01288904T1 - T3 -0.048 0.016 22.222 -3.090 0.014

cg01288904T2 - T3 -0.052 0.016 22.222 -3.340 0.008

cg13047386T1 - T2 0.026 0.018 22.222 1.452 0.332

cg13047386T1 - T3 -0.021 0.018 22.222 -1.152 0.493

cg13047386T2 - T3 -0.047 0.018 22.222 -2.604 0.041

cg19961720T1 - T2 0.019 0.011 22.222 1.738 0.214

cg19961720T1 - T3 -0.018 0.011 22.222 -1.647 0.247

cg19961720T2 - T3 -0.038 0.011 22.222 -3.386 0.007

cg10122477T1 - T2 -0.016 0.024 22.222 -0.644 0.798

cg10122477T1 - T3 0.040 0.024 22.222 1.651 0.246

cg10122477T2 - T3 0.056 0.024 22.222 2.295 0.077

cg20170989T1 - T2 -0.023 0.015 22.222 -1.573 0.278

cg20170989T1 - T3 0.018 0.015 22.222 1.231 0.448

cg20170989T2 - T3 0.042 0.015 22.222 2.804 0.027

cg01942675T1 - T2 -0.014 0.013 22.222 -1.065 0.545

cg01942675T1 - T3 0.034 0.013 22.222 2.652 0.037

cg01942675T2 - T3 0.048 0.013 22.222 3.717 0.003

cg22810704T1 - T2 0.021 0.010 22.222 2.105 0.112

cg22810704T1 - T3 -0.018 0.010 22.222 -1.870 0.171

cg22810704T2 - T3 -0.039 0.010 22.222 -3.975 0.002

cg03424127T1 - T2 0.001 0.012 22.222 0.093 0.995

cg03424127T1 - T3 0.045 0.012 22.222 3.857 0.002

cg03424127T2 - T3 0.044 0.012 22.222 3.765 0.003

cg07208691T1 - T2 -0.002 0.012 22.222 -0.154 0.987

cg07208691T1 - T3 -0.040 0.012 22.222 -3.437 0.006

cg07208691T2 - T3 -0.038 0.012 22.222 -3.284 0.009

cg13535489T1 - T2 0.025 0.009 22.222 2.647 0.037

cg13535489T1 - T3 -0.013 0.009 22.222 -1.378 0.369

cg13535489T2 - T3 -0.038 0.009 22.222 -4.025 0.002

cg21475781T1 - T2 -0.012 0.011 22.222 -1.115 0.515

cg21475781T1 - T3 -0.038 0.011 22.222 -3.453 0.006

cg21475781T2 - T3 -0.025 0.011 22.222 -2.337 0.071

cg17498451T1 - T2 0.008 0.010 22.222 0.764 0.729

cg17498451T1 - T3 -0.028 0.010 22.222 -2.650 0.037

cg17498451T2 - T3 -0.036 0.010 22.222 -3.414 0.007

cg00057134T1 - T2 -0.031 0.021 22.222 -1.453 0.332

cg00057134T1 - T3 0.027 0.021 22.222 1.270 0.426

cg00057134T2 - T3 0.058 0.021 22.222 2.722 0.032

cg17102034T1 - T2 -0.017 0.011 22.222 -1.467 0.325

cg17102034T1 - T3 0.020 0.011 22.222 1.712 0.223

cg17102034T2 - T3 0.036 0.011 22.222 3.179 0.012

cg18972885T1 - T2 -0.031 0.016 22.222 -1.886 0.166

cg18972885T1 - T3 0.017 0.016 22.222 1.054 0.551

cg18972885T2 - T3 0.048 0.016 22.222 2.940 0.020

cg25863967T1 - T2 -0.020 0.026 22.222 -0.756 0.733

cg25863967T1 - T3 0.075 0.026 22.222 2.891 0.022

cg25863967T2 - T3 0.095 0.026 22.222 3.647 0.004

cg07230182T1 - T2 0.013 0.013 22.222 0.977 0.599

cg07230182T1 - T3 -0.027 0.013 22.222 -2.034 0.127

cg07230182T2 - T3 -0.040 0.013 22.222 -3.011 0.017

cg26101468T1 - T2 0.000 0.014 22.222 0.035 0.999

cg26101468T1 - T3 0.037 0.014 22.222 2.635 0.038

cg26101468T2 - T3 0.037 0.014 22.222 2.600 0.041

cg09597672T1 - T2 -0.017 0.009 22.222 -1.899 0.162

cg09597672T1 - T3 -0.040 0.009 22.222 -4.399 0.001

cg09597672T2 - T3 -0.023 0.009 22.222 -2.499 0.051

cg01698998T1 - T2 0.009 0.014 22.222 0.679 0.778

cg01698998T1 - T3 -0.027 0.014 22.222 -1.953 0.147

cg01698998T2 - T3 -0.037 0.014 22.222 -2.632 0.039

cg11919785T1 - T2 0.018 0.009 22.222 1.954 0.147

cg11919785T1 - T3 -0.017 0.009 22.222 -1.775 0.201

cg11919785T2 - T3 -0.035 0.009 22.222 -3.729 0.003

cg17373310T1 - T2 -0.010 0.026 22.222 -0.387 0.921

cg17373310T1 - T3 -0.079 0.026 22.222 -3.102 0.014

cg17373310T2 - T3 -0.069 0.026 22.222 -2.714 0.032

cg17890044T1 - T2 0.011 0.010 22.222 1.121 0.512

cg17890044T1 - T3 -0.018 0.010 22.222 -1.896 0.163

cg17890044T2 - T3 -0.029 0.010 22.222 -3.017 0.017

cg00112320T1 - T2 0.024 0.012 22.222 2.037 0.127

cg00112320T1 - T3 -0.022 0.012 22.222 -1.868 0.171

cg00112320T2 - T3 -0.045 0.012 22.222 -3.906 0.002

cg08831917T1 - T2 -0.007 0.012 22.222 -0.564 0.840

cg08831917T1 - T3 -0.041 0.012 22.222 -3.419 0.007

cg08831917T2 - T3 -0.034 0.012 22.222 -2.855 0.024

cg12052114T1 - T2 -0.001 0.016 22.222 -0.036 0.999

cg12052114T1 - T3 -0.034 0.016 22.222 -2.060 0.122

cg12052114T2 - T3 -0.033 0.016 22.222 -2.024 0.130

cg26019533T1 - T2 0.001 0.010 22.222 0.075 0.997

cg26019533T1 - T3 0.034 0.010 22.222 3.281 0.009

cg26019533T2 - T3 0.033 0.010 22.222 3.206 0.011

cg16461248T1 - T2 0.011 0.012 22.222 0.963 0.607

cg16461248T1 - T3 -0.021 0.012 22.222 -1.818 0.187

cg16461248T2 - T3 -0.033 0.012 22.222 -2.781 0.028

cg04799232T1 - T2 0.015 0.010 22.222 1.499 0.310

cg04799232T1 - T3 -0.018 0.010 22.222 -1.843 0.179

cg04799232T2 - T3 -0.033 0.010 22.222 -3.342 0.008

cg13816552T1 - T2 0.022 0.020 22.222 1.143 0.499

cg13816552T1 - T3 -0.023 0.020 22.222 -1.165 0.486

cg13816552T2 - T3 -0.045 0.020 22.222 -2.308 0.075

cg16409883T1 - T2 -0.012 0.015 22.222 -0.759 0.731

cg16409883T1 - T3 0.042 0.015 22.222 2.734 0.031

cg16409883T2 - T3 0.053 0.015 22.222 3.493 0.006

cg16493540T1 - T2 0.001 0.011 22.222 0.082 0.996

cg16493540T1 - T3 0.031 0.011 22.222 2.732 0.031

cg16493540T2 - T3 0.030 0.011 22.222 2.650 0.037

cg20261167T1 - T2 -0.008 0.012 22.222 -0.624 0.809

cg20261167T1 - T3 0.025 0.012 22.222 2.023 0.130

cg20261167T2 - T3 0.033 0.012 22.222 2.647 0.038

cg06636222T1 - T2 0.006 0.013 22.222 0.471 0.885

cg06636222T1 - T3 -0.035 0.013 22.222 -2.778 0.028

cg06636222T2 - T3 -0.041 0.013 22.222 -3.249 0.010

cg07699648T1 - T2 -0.002 0.014 22.222 -0.113 0.993

cg07699648T1 - T3 -0.047 0.014 22.222 -3.272 0.009

cg07699648T2 - T3 -0.045 0.014 22.222 -3.159 0.012

cg06338120T1 - T2 0.021 0.012 22.222 1.760 0.206

cg06338120T1 - T3 -0.021 0.012 22.222 -1.769 0.203

cg06338120T2 - T3 -0.043 0.012 22.222 -3.529 0.005

cg15999165T1 - T2 0.007 0.011 22.222 0.608 0.817

cg15999165T1 - T3 0.033 0.011 22.222 3.019 0.017

cg15999165T2 - T3 0.026 0.011 22.222 2.410 0.061

cg02395581T1 - T2 0.003 0.033 22.222 0.101 0.994

cg02395581T1 - T3 -0.070 0.033 22.222 -2.153 0.102

cg02395581T2 - T3 -0.073 0.033 22.222 -2.254 0.084

cg12249572T1 - T2 -0.011 0.021 22.222 -0.500 0.872

cg12249572T1 - T3 0.029 0.021 22.222 1.387 0.365

cg12249572T2 - T3 0.040 0.021 22.222 1.887 0.166

cg16730327T1 - T2 -0.036 0.017 22.222 -2.143 0.104

cg16730327T1 - T3 -0.003 0.017 22.222 -0.166 0.985

cg16730327T2 - T3 0.033 0.017 22.222 1.976 0.141

cg27182548T1 - T2 -0.032 0.016 22.222 -1.945 0.150

cg27182548T1 - T3 0.029 0.016 22.222 1.788 0.197

cg27182548T2 - T3 0.062 0.016 22.222 3.733 0.003

cg02747341T1 - T2 0.006 0.013 22.222 0.450 0.895

cg02747341T1 - T3 -0.024 0.013 22.222 -1.857 0.175

cg02747341T2 - T3 -0.030 0.013 22.222 -2.307 0.076

cg05973623T1 - T2 -0.051 0.015 21.304 -3.360 0.008

cg05973623T1 - T3 0.031 0.015 21.304 2.053 0.124

cg05973623T2 - T3 0.083 0.015 21.230 5.632 0.000

cg25034117T1 - T2 0.007 0.011 22.222 0.613 0.814

cg25034117T1 - T3 -0.026 0.011 22.222 -2.296 0.077

cg25034117T2 - T3 -0.032 0.011 22.222 -2.910 0.021

cg14198860T1 - T2 0.009 0.011 22.222 0.810 0.701

cg14198860T1 - T3 -0.027 0.011 22.222 -2.364 0.067

cg14198860T2 - T3 -0.036 0.011 22.222 -3.174 0.012

cg07452217T1 - T2 0.006 0.028 22.222 0.197 0.979

cg07452217T1 - T3 0.079 0.028 22.222 2.808 0.026

cg07452217T2 - T3 0.073 0.028 22.222 2.611 0.040

cg02606279T1 - T2 -0.004 0.012 22.222 -0.373 0.927

cg02606279T1 - T3 0.040 0.012 22.222 3.364 0.008

cg02606279T2 - T3 0.044 0.012 22.222 3.736 0.003

cg00500027T1 - T2 0.013 0.012 22.222 1.089 0.530

cg00500027T1 - T3 -0.024 0.012 22.222 -1.934 0.153

cg00500027T2 - T3 -0.037 0.012 22.222 -3.024 0.016

cg15270813T1 - T2 -0.017 0.019 22.222 -0.889 0.652

cg15270813T1 - T3 0.036 0.019 22.222 1.897 0.163

cg15270813T2 - T3 0.053 0.019 22.222 2.786 0.028

cg14850601T1 - T2 0.022 0.011 22.222 2.007 0.134

cg14850601T1 - T3 -0.023 0.011 22.222 -2.152 0.102

cg14850601T2 - T3 -0.045 0.011 22.222 -4.159 0.001

cg05313644T1 - T2 -0.005 0.016 22.222 -0.314 0.947

cg05313644T1 - T3 0.038 0.016 22.222 2.360 0.068

cg05313644T2 - T3 0.043 0.016 22.222 2.674 0.035

cg02525930T1 - T2 0.006 0.023 22.222 0.275 0.959

cg02525930T1 - T3 -0.048 0.023 22.222 -2.106 0.111

cg02525930T2 - T3 -0.055 0.023 22.222 -2.381 0.065

cg16652347T1 - T2 0.000 0.015 22.222 -0.029 1.000

cg16652347T1 - T3 -0.032 0.015 22.222 -2.106 0.111

cg16652347T2 - T3 -0.032 0.015 22.222 -2.077 0.118

cg25964019T1 - T2 0.034 0.021 22.222 1.661 0.242

cg25964019T1 - T3 -0.039 0.021 22.222 -1.912 0.159

cg25964019T2 - T3 -0.073 0.021 22.222 -3.573 0.005

cg06343732T1 - T2 0.009 0.012 22.222 0.778 0.720

cg06343732T1 - T3 -0.027 0.012 22.222 -2.295 0.077

cg06343732T2 - T3 -0.036 0.012 22.222 -3.072 0.015

cg01927901T1 - T2 0.004 0.009 22.222 0.447 0.896

cg01927901T1 - T3 -0.033 0.009 22.222 -3.650 0.004

cg01927901T2 - T3 -0.037 0.009 22.222 -4.097 0.001

cg17869231T1 - T2 0.005 0.013 22.222 0.392 0.919

cg17869231T1 - T3 -0.046 0.013 22.222 -3.475 0.006

cg17869231T2 - T3 -0.051 0.013 22.222 -3.867 0.002

cg20253689T1 - T2 0.003 0.017 22.222 0.184 0.982

cg20253689T1 - T3 -0.046 0.017 22.222 -2.681 0.035

cg20253689T2 - T3 -0.049 0.017 22.222 -2.865 0.023

cg16771104T1 - T2 -0.018 0.018 22.222 -0.992 0.589

cg16771104T1 - T3 0.037 0.018 22.222 1.998 0.136

cg16771104T2 - T3 0.055 0.018 22.222 2.991 0.018

cg07369031T1 - T2 0.020 0.012 22.222 1.656 0.244

cg07369031T1 - T3 -0.023 0.012 22.222 -1.895 0.164

cg07369031T2 - T3 -0.043 0.012 22.222 -3.551 0.005

cg05352635T1 - T2 -0.019 0.020 22.222 -0.916 0.636

cg05352635T1 - T3 0.057 0.020 22.222 2.805 0.027

cg05352635T2 - T3 0.075 0.020 22.222 3.721 0.003

cg06538549T1 - T2 0.008 0.015 22.222 0.524 0.860

cg06538549T1 - T3 -0.042 0.015 22.222 -2.813 0.026

cg06538549T2 - T3 -0.050 0.015 22.222 -3.337 0.008

cg03275107T1 - T2 -0.014 0.009 22.222 -1.549 0.288

cg03275107T1 - T3 -0.033 0.009 22.222 -3.564 0.005

cg03275107T2 - T3 -0.019 0.009 22.222 -2.015 0.132

cg09719231T1 - T2 -0.005 0.010 22.222 -0.544 0.851

cg09719231T1 - T3 -0.042 0.010 22.222 -4.228 0.001

cg09719231T2 - T3 -0.036 0.010 22.222 -3.685 0.004

cg03322633T1 - T2 -0.008 0.011 22.222 -0.751 0.736

cg03322633T1 - T3 -0.037 0.011 22.222 -3.463 0.006

cg03322633T2 - T3 -0.029 0.011 22.222 -2.712 0.033

cg10535845T1 - T2 0.017 0.012 22.222 1.420 0.348

cg10535845T1 - T3 -0.032 0.012 22.222 -2.718 0.032

cg10535845T2 - T3 -0.048 0.012 22.222 -4.138 0.001

cg05257483T1 - T2 -0.003 0.014 22.222 -0.243 0.968

cg05257483T1 - T3 0.043 0.014 22.222 3.022 0.016

cg05257483T2 - T3 0.046 0.014 22.222 3.265 0.009

cg02412705T1 - T2 -0.008 0.009 22.222 -0.903 0.644

cg02412705T1 - T3 -0.038 0.009 22.222 -4.493 0.001

cg02412705T2 - T3 -0.031 0.009 22.222 -3.590 0.004

cg13906792T1 - T2 0.010 0.041 22.222 0.238 0.969

cg13906792T1 - T3 -0.083 0.041 22.222 -2.038 0.127

cg13906792T2 - T3 -0.093 0.041 22.222 -2.276 0.080

cg08516993T1 - T2 -0.043 0.024 22.222 -1.816 0.187

cg08516993T1 - T3 0.035 0.024 22.222 1.478 0.320

cg08516993T2 - T3 0.079 0.024 22.222 3.294 0.009

cg22372070T1 - T2 0.020 0.016 22.222 1.223 0.452

cg22372070T1 - T3 -0.022 0.016 22.222 -1.341 0.388

cg22372070T2 - T3 -0.042 0.016 22.222 -2.564 0.045

cg13550230T1 - T2 -0.006 0.009 22.222 -0.678 0.779

cg13550230T1 - T3 -0.036 0.009 22.222 -3.853 0.002

cg13550230T2 - T3 -0.029 0.009 22.222 -3.176 0.012

cg15004284T1 - T2 0.001 0.012 22.222 0.094 0.995

cg15004284T1 - T3 -0.038 0.012 22.222 -3.170 0.012

cg15004284T2 - T3 -0.040 0.012 22.222 -3.264 0.009

cg02766482T1 - T2 0.004 0.013 22.222 0.280 0.958

cg02766482T1 - T3 0.038 0.013 22.222 2.914 0.021

cg02766482T2 - T3 0.034 0.013 22.222 2.633 0.039

cg16790291T1 - T2 -0.004 0.016 22.222 -0.257 0.964

cg16790291T1 - T3 0.035 0.016 22.222 2.244 0.086

cg16790291T2 - T3 0.039 0.016 22.222 2.501 0.051

cg27374572T1 - T2 0.013 0.017 22.222 0.786 0.715

cg27374572T1 - T3 -0.043 0.017 22.222 -2.555 0.046

cg27374572T2 - T3 -0.056 0.017 22.222 -3.341 0.008

cg16087015T1 - T2 -0.015 0.019 22.222 -0.831 0.688

cg16087015T1 - T3 0.018 0.019 22.222 0.948 0.616

cg16087015T2 - T3 0.033 0.019 22.222 1.779 0.200

cg06416026T1 - T2 -0.001 0.009 22.222 -0.075 0.997

cg06416026T1 - T3 -0.032 0.009 22.222 -3.381 0.007

cg06416026T2 - T3 -0.031 0.009 22.222 -3.306 0.009

cg09254768T1 - T2 0.013 0.015 22.222 0.871 0.664

cg09254768T1 - T3 -0.028 0.015 22.222 -1.934 0.153

cg09254768T2 - T3 -0.041 0.015 22.222 -2.805 0.027

cg23626122T1 - T2 -0.019 0.014 22.222 -1.373 0.371

cg23626122T1 - T3 0.016 0.014 22.222 1.170 0.483

cg23626122T2 - T3 0.036 0.014 22.222 2.543 0.047

cg08462358T1 - T2 0.020 0.011 22.222 1.805 0.191

cg08462358T1 - T3 -0.028 0.011 22.222 -2.518 0.049

cg08462358T2 - T3 -0.048 0.011 22.222 -4.323 0.001

cg10620457T1 - T2 -0.002 0.009 22.222 -0.261 0.963

cg10620457T1 - T3 -0.037 0.009 22.222 -3.896 0.002

cg10620457T2 - T3 -0.034 0.009 22.222 -3.634 0.004

cg18135938T1 - T2 -0.003 0.020 22.222 -0.143 0.989

cg18135938T1 - T3 -0.060 0.020 22.222 -2.980 0.018

cg18135938T2 - T3 -0.057 0.020 22.222 -2.838 0.025

cg16225011T1 - T2 0.006 0.009 22.222 0.679 0.778

cg16225011T1 - T3 -0.026 0.009 22.222 -2.770 0.029

cg16225011T2 - T3 -0.032 0.009 22.222 -3.449 0.006

cg18804921T1 - T2 0.000 0.013 22.222 -0.006 1.000

cg18804921T1 - T3 -0.032 0.013 22.222 -2.383 0.065

cg18804921T2 - T3 -0.032 0.013 22.222 -2.377 0.066

cg18394275T1 - T2 0.003 0.010 22.222 0.300 0.952

cg18394275T1 - T3 -0.039 0.010 22.222 -3.891 0.002

cg18394275T2 - T3 -0.042 0.010 22.222 -4.191 0.001

cg23087577T1 - T2 -0.010 0.008 22.222 -1.219 0.455

cg23087577T1 - T3 -0.035 0.008 22.222 -4.176 0.001

cg23087577T2 - T3 -0.025 0.008 22.222 -2.956 0.019

cg09381022T1 - T2 0.021 0.012 22.222 1.819 0.186

cg09381022T1 - T3 -0.020 0.012 22.222 -1.694 0.230

cg09381022T2 - T3 -0.040 0.012 22.222 -3.513 0.005

cg11269166T1 - T2 0.006 0.015 22.222 0.423 0.907

cg11269166T1 - T3 0.040 0.015 22.222 2.595 0.042

cg11269166T2 - T3 0.033 0.015 22.222 2.172 0.098

cg01729652T1 - T2 0.000 0.015 22.222 -0.008 1.000

cg01729652T1 - T3 -0.046 0.015 22.222 -3.032 0.016

cg01729652T2 - T3 -0.046 0.015 22.222 -3.025 0.016

cg02794319T1 - T2 -0.025 0.020 22.222 -1.211 0.459

cg02794319T1 - T3 0.038 0.020 22.222 1.871 0.171

cg02794319T2 - T3 0.063 0.020 22.222 3.081 0.014

cg18776016T1 - T2 0.006 0.031 22.222 0.182 0.982

cg18776016T1 - T3 -0.065 0.031 22.222 -2.121 0.108

cg18776016T2 - T3 -0.070 0.031 22.222 -2.303 0.076

cg14645943T1 - T2 -0.028 0.028 22.222 -0.989 0.591

cg14645943T1 - T3 0.056 0.028 22.222 2.001 0.135

cg14645943T2 - T3 0.084 0.028 22.222 2.990 0.018

cg25116398T1 - T2 -0.046 0.013 22.222 -3.556 0.005

cg25116398T1 - T3 0.023 0.013 22.222 1.770 0.203

cg25116398T2 - T3 0.068 0.013 22.222 5.325 0.000

cg22859585T1 - T2 0.013 0.014 22.222 0.865 0.667

cg22859585T1 - T3 -0.029 0.014 22.222 -1.985 0.139

cg22859585T2 - T3 -0.041 0.014 22.222 -2.850 0.024

cg06622151T1 - T2 -0.025 0.009 22.222 -2.695 0.034

cg06622151T1 - T3 0.008 0.009 22.222 0.894 0.650

cg06622151T2 - T3 0.033 0.009 22.222 3.589 0.004

cg00071739T1 - T2 0.021 0.013 22.222 1.705 0.226

cg00071739T1 - T3 -0.026 0.013 22.222 -2.074 0.118

cg00071739T2 - T3 -0.048 0.013 22.222 -3.779 0.003

cg20226764T1 - T2 -0.013 0.010 22.222 -1.283 0.419

cg20226764T1 - T3 0.027 0.010 22.222 2.612 0.040

cg20226764T2 - T3 0.040 0.010 22.222 3.895 0.002

cg05684714T1 - T2 -0.037 0.027 22.222 -1.369 0.374

cg05684714T1 - T3 0.077 0.027 22.222 2.820 0.026

cg05684714T2 - T3 0.114 0.027 22.222 4.189 0.001

cg12361295T1 - T2 0.005 0.011 22.222 0.462 0.890

cg12361295T1 - T3 -0.029 0.011 22.222 -2.696 0.034

cg12361295T2 - T3 -0.034 0.011 22.222 -3.158 0.012

cg01283343T1 - T2 -0.005 0.014 22.222 -0.346 0.936

cg01283343T1 - T3 -0.056 0.014 22.222 -3.933 0.002

cg01283343T2 - T3 -0.051 0.014 22.222 -3.587 0.004

cg19388032T1 - T2 0.014 0.011 22.222 1.181 0.476

cg19388032T1 - T3 -0.023 0.011 22.222 -2.012 0.133

cg19388032T2 - T3 -0.037 0.011 22.222 -3.193 0.011

cg15123849T1 - T2 -0.025 0.015 22.222 -1.699 0.228

cg15123849T1 - T3 0.025 0.015 22.222 1.643 0.249

cg15123849T2 - T3 0.050 0.015 22.222 3.342 0.008

cg18493242T1 - T2 -0.028 0.019 21.748 -1.459 0.330

cg18493242T1 - T3 0.030 0.019 21.748 1.572 0.279

cg18493242T2 - T3 0.058 0.018 21.233 3.143 0.013

cg27285720T1 - T2 0.005 0.023 22.222 0.211 0.976

cg27285720T1 - T3 0.043 0.023 22.222 1.912 0.159

cg27285720T2 - T3 0.038 0.023 22.222 1.702 0.227

cg19896705T1 - T2 -0.010 0.015 22.222 -0.680 0.777

cg19896705T1 - T3 0.014 0.015 22.222 0.931 0.627

cg19896705T2 - T3 0.024 0.015 22.222 1.611 0.262

cg06921368T1 - T2 0.008 0.013 22.222 0.631 0.805

cg06921368T1 - T3 -0.031 0.013 22.222 -2.271 0.081

cg06921368T2 - T3 -0.039 0.013 22.222 -2.902 0.022

cg21439672T1 - T2 0.002 0.014 22.222 0.161 0.986

cg21439672T1 - T3 -0.037 0.014 22.222 -2.644 0.038

cg21439672T2 - T3 -0.039 0.014 22.222 -2.805 0.027

cg24842760T1 - T2 -0.004 0.018 22.222 -0.234 0.970

cg24842760T1 - T3 0.037 0.018 22.222 2.096 0.114

cg24842760T2 - T3 0.041 0.018 22.222 2.330 0.072

cg04212092T1 - T2 -0.006 0.009 22.222 -0.658 0.790

cg04212092T1 - T3 0.024 0.009 22.222 2.712 0.033

cg04212092T2 - T3 0.029 0.009 22.222 3.370 0.007

cg23642392T1 - T2 0.000 0.018 22.222 -0.019 1.000

cg23642392T1 - T3 -0.051 0.018 22.222 -2.894 0.022

cg23642392T2 - T3 -0.050 0.018 22.222 -2.875 0.023

cg08535978T1 - T2 -0.019 0.018 22.222 -1.039 0.561

cg08535978T1 - T3 0.038 0.018 22.222 2.109 0.111

cg08535978T2 - T3 0.057 0.018 22.222 3.148 0.012

cg02468649T1 - T2 0.000 0.013 22.222 -0.033 0.999

cg02468649T1 - T3 -0.035 0.013 22.222 -2.669 0.036

cg02468649T2 - T3 -0.035 0.013 22.222 -2.636 0.038

cg05839294T1 - T2 0.026 0.011 22.222 2.316 0.074

cg05839294T1 - T3 -0.019 0.011 22.222 -1.749 0.210

cg05839294T2 - T3 -0.045 0.011 22.222 -4.065 0.001

cg15988345T1 - T2 0.008 0.013 22.222 0.642 0.799

cg15988345T1 - T3 -0.027 0.013 22.222 -2.058 0.122

cg15988345T2 - T3 -0.035 0.013 22.222 -2.700 0.034

cg07364520T1 - T2 0.027 0.037 22.222 0.727 0.750

cg07364520T1 - T3 -0.101 0.037 22.222 -2.700 0.033

cg07364520T2 - T3 -0.128 0.037 22.222 -3.427 0.006

cg12673213T1 - T2 -0.005 0.014 22.222 -0.347 0.936

cg12673213T1 - T3 -0.064 0.014 22.222 -4.734 0.000

cg12673213T2 - T3 -0.060 0.014 22.222 -4.387 0.001

cg00823110T1 - T2 0.034 0.012 22.222 2.805 0.027

cg00823110T1 - T3 -0.004 0.012 22.222 -0.315 0.947

cg00823110T2 - T3 -0.037 0.012 22.222 -3.120 0.013

cg13099417T1 - T2 -0.031 0.012 22.222 -2.531 0.048

cg13099417T1 - T3 0.016 0.012 22.222 1.318 0.400

cg13099417T2 - T3 0.047 0.012 22.222 3.849 0.002

cg09363752T1 - T2 -0.030 0.026 22.222 -1.166 0.485

cg09363752T1 - T3 0.028 0.026 22.222 1.109 0.518

cg09363752T2 - T3 0.058 0.026 22.222 2.275 0.080

cg01884781T1 - T2 -0.021 0.015 22.222 -1.395 0.360

cg01884781T1 - T3 -0.074 0.015 22.222 -4.933 0.000

cg01884781T2 - T3 -0.053 0.015 22.222 -3.538 0.005

cg20558112T1 - T2 -0.012 0.015 22.222 -0.808 0.702

cg20558112T1 - T3 0.028 0.015 22.222 1.898 0.163

cg20558112T2 - T3 0.040 0.015 22.222 2.706 0.033

cg12724086T1 - T2 -0.023 0.017 22.222 -1.375 0.371

cg12724086T1 - T3 0.015 0.017 22.222 0.908 0.641

cg12724086T2 - T3 0.038 0.017 22.222 2.283 0.079

cg08523068T1 - T2 0.020 0.017 22.222 1.218 0.455

cg08523068T1 - T3 -0.019 0.017 22.222 -1.128 0.507

cg08523068T2 - T3 -0.039 0.017 22.222 -2.346 0.070

cg10406180T1 - T2 -0.014 0.011 22.222 -1.243 0.441

cg10406180T1 - T3 -0.043 0.011 22.222 -3.749 0.003

cg10406180T2 - T3 -0.029 0.011 22.222 -2.506 0.050

cg16010788T1 - T2 0.027 0.019 22.222 1.404 0.356

cg16010788T1 - T3 -0.026 0.019 22.222 -1.353 0.382

cg16010788T2 - T3 -0.053 0.019 22.222 -2.757 0.030

cg00817377T1 - T2 0.014 0.019 22.222 0.738 0.744

cg00817377T1 - T3 -0.047 0.019 22.222 -2.507 0.050

cg00817377T2 - T3 -0.061 0.019 22.222 -3.245 0.010

cg21896766T1 - T2 -0.032 0.024 22.222 -1.311 0.404

cg21896766T1 - T3 0.036 0.024 22.222 1.475 0.322

cg21896766T2 - T3 0.068 0.024 22.222 2.786 0.028

cg25922934T1 - T2 -0.001 0.015 22.222 -0.044 0.999

cg25922934T1 - T3 -0.035 0.015 22.222 -2.397 0.063

cg25922934T2 - T3 -0.034 0.015 22.222 -2.353 0.069

cg11765615T1 - T2 -0.014 0.010 22.222 -1.469 0.324

cg11765615T1 - T3 0.017 0.010 22.222 1.678 0.235

cg11765615T2 - T3 0.031 0.010 22.222 3.147 0.012

cg10021614T1 - T2 0.022 0.014 22.222 1.597 0.268

cg10021614T1 - T3 -0.013 0.014 22.222 -0.899 0.647

cg10021614T2 - T3 -0.035 0.014 22.222 -2.495 0.052

cg20181513T1 - T2 -0.028 0.024 22.222 -1.169 0.483

cg20181513T1 - T3 0.038 0.024 22.222 1.560 0.283

cg20181513T2 - T3 0.066 0.024 22.222 2.729 0.031

cg03146706T1 - T2 0.057 0.018 22.222 3.151 0.012

cg03146706T1 - T3 -0.020 0.018 22.222 -1.098 0.525

cg03146706T2 - T3 -0.077 0.018 22.222 -4.249 0.001

cg04534110T1 - T2 -0.006 0.014 22.222 -0.445 0.897

cg04534110T1 - T3 -0.034 0.014 22.222 -2.393 0.064

cg04534110T2 - T3 -0.028 0.014 22.222 -1.948 0.149

cg05461153T1 - T2 0.010 0.014 22.222 0.764 0.728

cg05461153T1 - T3 -0.036 0.014 22.222 -2.604 0.041

cg05461153T2 - T3 -0.046 0.014 22.222 -3.368 0.007

cg09292030T1 - T2 0.022 0.014 22.222 1.638 0.251

cg09292030T1 - T3 -0.025 0.014 22.222 -1.790 0.196

cg09292030T2 - T3 -0.047 0.014 22.222 -3.428 0.006

cg05206385T1 - T2 0.015 0.013 22.222 1.162 0.487

cg05206385T1 - T3 -0.026 0.013 22.222 -2.081 0.117

cg05206385T2 - T3 -0.041 0.013 22.222 -3.243 0.010

cg04016841T1 - T2 -0.019 0.014 22.222 -1.282 0.420

cg04016841T1 - T3 0.022 0.014 22.222 1.540 0.292

cg04016841T2 - T3 0.041 0.014 22.222 2.822 0.026

cg09707903T1 - T2 0.006 0.013 22.222 0.487 0.878

cg09707903T1 - T3 -0.042 0.013 22.222 -3.206 0.011

cg09707903T2 - T3 -0.048 0.013 22.222 -3.693 0.003

cg22721851T1 - T2 0.023 0.010 22.222 2.244 0.086

cg22721851T1 - T3 -0.009 0.010 22.222 -0.906 0.642

cg22721851T2 - T3 -0.032 0.010 22.222 -3.150 0.012

cg03206460T1 - T2 0.030 0.012 22.222 2.418 0.060

cg03206460T1 - T3 -0.016 0.012 22.222 -1.291 0.415

cg03206460T2 - T3 -0.045 0.012 22.222 -3.709 0.003

cg00772587T1 - T2 0.010 0.010 22.222 0.985 0.594

cg00772587T1 - T3 -0.037 0.010 22.222 -3.640 0.004

cg00772587T2 - T3 -0.046 0.010 22.222 -4.625 0.000

cg13557594T1 - T2 -0.012 0.012 22.222 -1.000 0.584

cg13557594T1 - T3 -0.038 0.012 22.222 -3.253 0.010

cg13557594T2 - T3 -0.026 0.012 22.222 -2.253 0.084

cg21306573T1 - T2 0.014 0.012 22.222 1.134 0.504

cg21306573T1 - T3 -0.019 0.012 22.222 -1.557 0.285

cg21306573T2 - T3 -0.034 0.012 22.222 -2.692 0.034

cg07320630T1 - T2 0.013 0.020 22.222 0.638 0.801

cg07320630T1 - T3 -0.048 0.020 22.222 -2.338 0.071

cg07320630T2 - T3 -0.061 0.020 22.222 -2.975 0.018

cg26599871T1 - T2 0.008 0.011 22.222 0.728 0.750

cg26599871T1 - T3 -0.026 0.011 22.222 -2.324 0.073

cg26599871T2 - T3 -0.034 0.011 22.222 -3.052 0.015

cg06638704T1 - T2 0.015 0.010 22.222 1.439 0.339

cg06638704T1 - T3 -0.021 0.010 22.222 -2.013 0.132

cg06638704T2 - T3 -0.036 0.010 22.222 -3.452 0.006

cg13194897T1 - T2 0.014 0.013 22.222 1.073 0.540

cg13194897T1 - T3 -0.028 0.013 22.222 -2.134 0.106

cg13194897T2 - T3 -0.042 0.013 22.222 -3.207 0.011

cg22677650T1 - T2 0.004 0.010 22.222 0.356 0.933

cg22677650T1 - T3 -0.034 0.010 22.222 -3.292 0.009

cg22677650T2 - T3 -0.037 0.010 22.222 -3.648 0.004

cg21472737T1 - T2 0.009 0.017 22.222 0.548 0.849

cg21472737T1 - T3 -0.035 0.017 22.222 -2.110 0.111

cg21472737T2 - T3 -0.045 0.017 22.222 -2.657 0.037

cg07943346T1 - T2 0.015 0.015 22.222 0.958 0.610

cg07943346T1 - T3 -0.048 0.015 22.222 -3.111 0.013

cg07943346T2 - T3 -0.062 0.015 22.222 -4.069 0.001

cg06952660T1 - T2 -0.011 0.020 22.222 -0.544 0.850

cg06952660T1 - T3 0.037 0.020 22.222 1.887 0.166

cg06952660T2 - T3 0.048 0.020 22.222 2.432 0.059

cg25450248T1 - T2 0.023 0.016 22.222 1.459 0.329

cg25450248T1 - T3 -0.033 0.016 22.222 -2.122 0.108

cg25450248T2 - T3 -0.056 0.016 22.222 -3.581 0.005

cg07261996T1 - T2 -0.018 0.009 22.222 -2.042 0.126

cg07261996T1 - T3 0.013 0.009 22.222 1.456 0.331

cg07261996T2 - T3 0.030 0.009 22.222 3.498 0.005

cg10105884T1 - T2 -0.008 0.017 22.222 -0.491 0.876

cg10105884T1 - T3 -0.032 0.017 22.222 -1.903 0.161

cg10105884T2 - T3 -0.024 0.017 22.222 -1.412 0.352

cg00708809T1 - T2 0.017 0.011 22.222 1.461 0.328

cg00708809T1 - T3 -0.022 0.011 22.222 -1.878 0.168

cg00708809T2 - T3 -0.038 0.011 22.222 -3.339 0.008

cg02086046T1 - T2 -0.014 0.012 22.222 -1.154 0.492

cg02086046T1 - T3 -0.051 0.012 22.222 -4.334 0.001

cg02086046T2 - T3 -0.037 0.012 22.222 -3.179 0.012

cg13823627T1 - T2 -0.018 0.025 22.222 -0.718 0.756

cg13823627T1 - T3 0.061 0.025 22.222 2.458 0.056

cg13823627T2 - T3 0.079 0.025 22.222 3.175 0.012

cg15946357T1 - T2 0.019 0.008 22.222 2.531 0.048

cg15946357T1 - T3 -0.017 0.008 22.222 -2.211 0.091

cg15946357T2 - T3 -0.036 0.008 22.222 -4.742 0.000

cg12871652T1 - T2 0.027 0.034 21.479 0.794 0.711

cg12871652T1 - T3 -0.067 0.034 21.479 -2.002 0.136

cg12871652T2 - T3 -0.094 0.032 21.221 -2.906 0.022

cg09583374T1 - T2 -0.019 0.014 22.222 -1.354 0.381

cg09583374T1 - T3 0.021 0.014 22.222 1.554 0.286

cg09583374T2 - T3 0.040 0.014 22.222 2.909 0.021

cg04923875T1 - T2 0.015 0.011 22.222 1.310 0.404

cg04923875T1 - T3 -0.031 0.011 22.222 -2.731 0.031

cg04923875T2 - T3 -0.045 0.011 22.222 -4.041 0.001

cg11262246T1 - T2 -0.001 0.021 22.222 -0.066 0.998

cg11262246T1 - T3 -0.059 0.021 22.222 -2.882 0.022

cg11262246T2 - T3 -0.058 0.021 22.222 -2.816 0.026

cg09727653T1 - T2 0.007 0.011 22.222 0.599 0.822

cg09727653T1 - T3 -0.040 0.011 22.222 -3.634 0.004

cg09727653T2 - T3 -0.046 0.011 22.222 -4.233 0.001

cg08008105T1 - T2 -0.003 0.014 22.222 -0.209 0.976

cg08008105T1 - T3 -0.045 0.014 22.222 -3.159 0.012

cg08008105T2 - T3 -0.042 0.014 22.222 -2.949 0.019

cg15173319T1 - T2 0.021 0.014 22.222 1.495 0.312

cg15173319T1 - T3 -0.030 0.014 22.222 -2.145 0.104

cg15173319T2 - T3 -0.051 0.014 22.222 -3.640 0.004

cg02406092T1 - T2 0.006 0.013 22.222 0.475 0.884

cg02406092T1 - T3 -0.033 0.013 22.222 -2.523 0.049

cg02406092T2 - T3 -0.040 0.013 22.222 -2.997 0.017

cg18839315T1 - T2 0.010 0.016 22.222 0.605 0.819

cg18839315T1 - T3 -0.031 0.016 22.222 -1.860 0.174

cg18839315T2 - T3 -0.041 0.016 22.222 -2.465 0.055

cg14353539T1 - T2 0.009 0.006 22.222 1.443 0.337

cg14353539T1 - T3 -0.025 0.006 22.222 -3.843 0.002

cg14353539T2 - T3 -0.034 0.006 22.222 -5.286 0.000

cg10363202T1 - T2 0.013 0.012 22.222 1.141 0.499

cg10363202T1 - T3 -0.025 0.012 22.222 -2.129 0.107

cg10363202T2 - T3 -0.038 0.012 22.222 -3.271 0.009

cg13672501T1 - T2 -0.012 0.009 22.222 -1.310 0.405

cg13672501T1 - T3 -0.038 0.009 22.222 -4.034 0.002

cg13672501T2 - T3 -0.026 0.009 22.222 -2.724 0.032

cg07509145T1 - T2 0.017 0.011 22.222 1.584 0.273

cg07509145T1 - T3 -0.027 0.011 22.222 -2.497 0.051

cg07509145T2 - T3 -0.044 0.011 22.222 -4.081 0.001

cg06776534T1 - T2 -0.013 0.019 20.421 -0.675 0.780

cg06776534T1 - T3 -0.087 0.020 20.659 -4.325 0.001

cg06776534T2 - T3 -0.074 0.019 20.421 -3.825 0.003

cg11698322T1 - T2 0.020 0.013 22.222 1.508 0.306

cg11698322T1 - T3 -0.026 0.013 22.222 -1.912 0.159

cg11698322T2 - T3 -0.046 0.013 22.222 -3.420 0.007

cg08146340T1 - T2 -0.018 0.017 22.222 -1.091 0.530

cg08146340T1 - T3 0.024 0.017 22.222 1.405 0.355

cg08146340T2 - T3 0.042 0.017 22.222 2.496 0.052

cg05300241T1 - T2 -0.018 0.014 22.222 -1.221 0.453

cg05300241T1 - T3 0.017 0.014 22.222 1.153 0.493

cg05300241T2 - T3 0.034 0.014 22.222 2.375 0.066

cg20799679T1 - T2 0.021 0.018 22.222 1.192 0.470

cg20799679T1 - T3 -0.031 0.018 22.222 -1.760 0.206

cg20799679T2 - T3 -0.052 0.018 22.222 -2.952 0.019

cg24531534T1 - T2 -0.003 0.018 22.222 -0.161 0.986

cg24531534T1 - T3 0.048 0.018 22.222 2.658 0.037

cg24531534T2 - T3 0.051 0.018 22.222 2.819 0.026

cg20643070T1 - T2 -0.016 0.015 22.222 -1.119 0.513

cg20643070T1 - T3 0.027 0.015 22.222 1.855 0.175

cg20643070T2 - T3 0.043 0.015 22.222 2.973 0.018

cg17691428T1 - T2 -0.019 0.012 22.222 -1.597 0.268

cg17691428T1 - T3 0.014 0.012 22.222 1.180 0.477

cg17691428T2 - T3 0.033 0.012 22.222 2.777 0.028

cg05046068T1 - T2 -0.014 0.014 22.222 -1.051 0.553

cg05046068T1 - T3 0.021 0.014 22.222 1.582 0.274

cg05046068T2 - T3 0.036 0.014 22.222 2.633 0.039

cg23751557T1 - T2 0.026 0.020 22.222 1.355 0.381

cg23751557T1 - T3 -0.030 0.020 22.222 -1.547 0.289

cg23751557T2 - T3 -0.057 0.020 22.222 -2.902 0.022

cg25670076T1 - T2 0.015 0.017 22.222 0.873 0.662

cg25670076T1 - T3 -0.024 0.017 22.222 -1.445 0.336

cg25670076T2 - T3 -0.039 0.017 22.222 -2.318 0.074

cg24631102T1 - T2 -0.019 0.030 22.222 -0.616 0.813

cg24631102T1 - T3 -0.086 0.030 22.222 -2.834 0.025

cg24631102T2 - T3 -0.067 0.030 22.222 -2.219 0.090

cg15421831T1 - T2 0.036 0.016 22.222 2.246 0.085

cg15421831T1 - T3 -0.015 0.016 22.222 -0.917 0.636

cg15421831T2 - T3 -0.051 0.016 22.222 -3.163 0.012

cg09750643T1 - T2 -0.003 0.015 22.222 -0.231 0.971

cg09750643T1 - T3 0.043 0.015 22.222 2.954 0.019

cg09750643T2 - T3 0.047 0.015 22.222 3.185 0.011

cg20470083T1 - T2 0.003 0.016 22.222 0.173 0.984

cg20470083T1 - T3 -0.035 0.016 22.222 -2.234 0.087

cg20470083T2 - T3 -0.038 0.016 22.222 -2.407 0.062

cg19199261T1 - T2 -0.009 0.014 22.222 -0.693 0.770

cg19199261T1 - T3 0.021 0.014 22.222 1.541 0.292

cg19199261T2 - T3 0.030 0.014 22.222 2.234 0.087

cg21322417T1 - T2 -0.006 0.023 22.222 -0.260 0.963

cg21322417T1 - T3 -0.072 0.023 22.222 -3.155 0.012

cg21322417T2 - T3 -0.066 0.023 22.222 -2.895 0.022

cg05317477T1 - T2 -0.031 0.024 22.222 -1.280 0.421

cg05317477T1 - T3 0.019 0.024 22.222 0.778 0.720

cg05317477T2 - T3 0.049 0.024 22.222 2.057 0.122

cg08712702T1 - T2 0.033 0.015 22.222 2.230 0.088

cg08712702T1 - T3 -0.035 0.015 22.222 -2.324 0.073

cg08712702T2 - T3 -0.068 0.015 22.222 -4.554 0.000

cg14292522T1 - T2 -0.010 0.016 22.222 -0.625 0.808

cg14292522T1 - T3 -0.054 0.016 22.222 -3.402 0.007

cg14292522T2 - T3 -0.044 0.016 22.222 -2.778 0.028

cg02955377T1 - T2 -0.020 0.017 22.222 -1.163 0.487

cg02955377T1 - T3 0.034 0.017 22.222 1.991 0.138

cg02955377T2 - T3 0.054 0.017 22.222 3.154 0.012

cg08028842T1 - T2 0.013 0.013 22.222 0.982 0.595

cg08028842T1 - T3 -0.022 0.013 22.222 -1.683 0.234

cg08028842T2 - T3 -0.035 0.013 22.222 -2.665 0.036

cg15159017T1 - T2 -0.001 0.012 22.222 -0.109 0.993

cg15159017T1 - T3 -0.031 0.012 22.222 -2.621 0.040

cg15159017T2 - T3 -0.030 0.012 22.222 -2.512 0.050

cg18323236T1 - T2 0.009 0.019 22.222 0.484 0.880

cg18323236T1 - T3 -0.056 0.019 22.222 -2.964 0.019

cg18323236T2 - T3 -0.065 0.019 22.222 -3.448 0.006

cg00081238T1 - T2 0.013 0.024 22.222 0.554 0.845

cg00081238T1 - T3 -0.054 0.024 22.222 -2.224 0.089

cg00081238T2 - T3 -0.067 0.024 22.222 -2.778 0.028

cg00993205T1 - T2 0.001 0.016 22.222 0.054 0.998

cg00993205T1 - T3 -0.036 0.016 22.222 -2.332 0.072

cg00993205T2 - T3 -0.037 0.016 22.222 -2.386 0.064

cg05273780T1 - T2 0.001 0.015 22.222 0.080 0.996

cg05273780T1 - T3 -0.046 0.015 22.222 -3.060 0.015

cg05273780T2 - T3 -0.047 0.015 22.222 -3.139 0.013

cg15070041T1 - T2 0.018 0.009 22.222 2.138 0.105

cg15070041T1 - T3 -0.016 0.009 22.222 -1.852 0.176

cg15070041T2 - T3 -0.034 0.009 22.222 -3.990 0.002

cg09901532T1 - T2 0.041 0.021 22.222 1.985 0.139

cg09901532T1 - T3 -0.017 0.021 22.222 -0.820 0.695

cg09901532T2 - T3 -0.058 0.021 22.222 -2.805 0.027

cg05475858T1 - T2 -0.028 0.023 22.222 -1.229 0.449

cg05475858T1 - T3 0.033 0.023 22.222 1.429 0.344

cg05475858T2 - T3 0.061 0.023 22.222 2.658 0.037

cg03315662T1 - T2 0.012 0.014 22.222 0.829 0.689

cg03315662T1 - T3 -0.037 0.014 22.222 -2.571 0.044

cg03315662T2 - T3 -0.049 0.014 22.222 -3.401 0.007

cg18117149T1 - T2 0.015 0.009 22.222 1.664 0.241

cg18117149T1 - T3 -0.024 0.009 22.222 -2.654 0.037

cg18117149T2 - T3 -0.039 0.009 22.222 -4.318 0.001

cg02556345T1 - T2 -0.011 0.019 22.222 -0.589 0.827

cg02556345T1 - T3 0.046 0.019 22.222 2.354 0.069

cg02556345T2 - T3 0.057 0.019 22.222 2.943 0.020

cg25401138T1 - T2 0.017 0.011 22.222 1.467 0.326

cg25401138T1 - T3 -0.022 0.011 22.222 -1.899 0.162

cg25401138T2 - T3 -0.038 0.011 22.222 -3.366 0.007

cg03640732T1 - T2 -0.018 0.024 22.222 -0.756 0.733

cg03640732T1 - T3 0.033 0.024 22.222 1.373 0.372

cg03640732T2 - T3 0.051 0.024 22.222 2.129 0.107

cg11134774T1 - T2 0.016 0.018 22.222 0.894 0.650

cg11134774T1 - T3 -0.042 0.018 22.222 -2.336 0.071

cg11134774T2 - T3 -0.057 0.018 22.222 -3.229 0.010

cg16397644T1 - T2 0.019 0.010 22.222 1.953 0.148

cg16397644T1 - T3 -0.022 0.010 22.222 -2.244 0.086

cg16397644T2 - T3 -0.040 0.010 22.222 -4.197 0.001

cg26503653T1 - T2 -0.013 0.011 22.222 -1.138 0.501

cg26503653T1 - T3 0.029 0.011 22.222 2.657 0.037

cg26503653T2 - T3 0.042 0.011 22.222 3.796 0.003

cg02206767T1 - T2 0.042 0.023 22.222 1.819 0.186

cg02206767T1 - T3 -0.030 0.023 22.222 -1.287 0.417

cg02206767T2 - T3 -0.071 0.023 22.222 -3.106 0.014

cg13044118T1 - T2 0.004 0.022 22.222 0.172 0.984

cg13044118T1 - T3 -0.046 0.022 22.222 -2.054 0.123

cg13044118T2 - T3 -0.050 0.022 22.222 -2.226 0.089

cg23082657T1 - T2 0.025 0.014 22.222 1.842 0.179

cg23082657T1 - T3 -0.032 0.014 22.222 -2.336 0.071

cg23082657T2 - T3 -0.057 0.014 22.222 -4.178 0.001

cg23206630T1 - T2 -0.021 0.021 22.222 -0.977 0.599

cg23206630T1 - T3 0.034 0.021 22.222 1.604 0.265

cg23206630T2 - T3 0.055 0.021 22.222 2.580 0.043

cg13561503T1 - T2 -0.001 0.016 22.222 -0.089 0.996

cg13561503T1 - T3 -0.049 0.016 22.222 -3.023 0.016

cg13561503T2 - T3 -0.047 0.016 22.222 -2.934 0.020

cg21812861T1 - T2 0.011 0.016 22.222 0.700 0.766

cg21812861T1 - T3 -0.025 0.016 22.222 -1.579 0.275

cg21812861T2 - T3 -0.036 0.016 22.222 -2.279 0.080

cg00783170T1 - T2 0.017 0.008 22.222 2.063 0.121

cg00783170T1 - T3 -0.021 0.008 22.222 -2.487 0.052

cg00783170T2 - T3 -0.038 0.008 22.222 -4.551 0.000

cg21441575T1 - T2 0.014 0.013 22.222 1.070 0.542

cg21441575T1 - T3 -0.024 0.013 22.222 -1.857 0.175

cg21441575T2 - T3 -0.038 0.013 22.222 -2.926 0.020

cg04537185T1 - T2 0.018 0.012 22.222 1.472 0.323

cg04537185T1 - T3 -0.026 0.012 22.222 -2.201 0.093

cg04537185T2 - T3 -0.044 0.012 22.222 -3.672 0.004

cg04904099T1 - T2 0.003 0.013 22.222 0.265 0.962

cg04904099T1 - T3 0.037 0.013 22.222 2.913 0.021

cg04904099T2 - T3 0.034 0.013 22.222 2.647 0.037

cg03398843T1 - T2 0.010 0.008 22.222 1.388 0.364

cg03398843T1 - T3 -0.027 0.008 22.222 -3.584 0.004

cg03398843T2 - T3 -0.037 0.008 22.222 -4.972 0.000

cg24101459T1 - T2 0.015 0.013 22.222 1.143 0.498

cg24101459T1 - T3 -0.024 0.013 22.222 -1.833 0.182

cg24101459T2 - T3 -0.039 0.013 22.222 -2.976 0.018

cg19639788T1 - T2 -0.005 0.018 22.222 -0.287 0.956

cg19639788T1 - T3 0.043 0.018 22.222 2.392 0.064

cg19639788T2 - T3 0.048 0.018 22.222 2.679 0.035

cg02872426T1 - T2 0.012 0.019 22.222 0.640 0.800

cg02872426T1 - T3 -0.048 0.019 22.222 -2.556 0.045

cg02872426T2 - T3 -0.060 0.019 22.222 -3.196 0.011

cg13922269T1 - T2 0.013 0.010 22.222 1.203 0.464

cg13922269T1 - T3 0.035 0.010 22.222 3.381 0.007

cg13922269T2 - T3 0.023 0.010 22.222 2.177 0.097

cg00443797T1 - T2 -0.008 0.020 22.222 -0.390 0.920

cg00443797T1 - T3 -0.070 0.020 22.222 -3.472 0.006

cg00443797T2 - T3 -0.062 0.020 22.222 -3.082 0.014

cg15004902T1 - T2 0.020 0.014 22.222 1.488 0.316

cg15004902T1 - T3 -0.028 0.014 22.222 -2.037 0.127

cg15004902T2 - T3 -0.048 0.014 22.222 -3.525 0.005

cg17820871T1 - T2 0.008 0.017 22.222 0.477 0.883

cg17820871T1 - T3 -0.044 0.017 22.222 -2.556 0.045

cg17820871T2 - T3 -0.052 0.017 22.222 -3.033 0.016

cg22047282T1 - T2 -0.022 0.014 22.222 -1.616 0.260

cg22047282T1 - T3 0.010 0.014 22.222 0.742 0.741

cg22047282T2 - T3 0.032 0.014 22.222 2.358 0.068

cg08933542T1 - T2 0.001 0.015 22.222 0.084 0.996

cg08933542T1 - T3 0.038 0.015 22.222 2.625 0.039

cg08933542T2 - T3 0.037 0.015 22.222 2.542 0.047

cg05252487T1 - T2 0.010 0.012 22.222 0.807 0.703

cg05252487T1 - T3 -0.028 0.012 22.222 -2.412 0.061

cg05252487T2 - T3 -0.038 0.012 22.222 -3.219 0.011

cg13732643T1 - T2 0.016 0.025 22.222 0.628 0.806

cg13732643T1 - T3 -0.050 0.025 22.222 -1.996 0.137

cg13732643T2 - T3 -0.066 0.025 22.222 -2.624 0.039

cg18696790T1 - T2 0.025 0.015 22.222 1.696 0.229

cg18696790T1 - T3 -0.023 0.015 22.222 -1.549 0.288

cg18696790T2 - T3 -0.048 0.015 22.222 -3.245 0.010

cg02795305T1 - T2 -0.003 0.015 22.222 -0.180 0.982

cg02795305T1 - T3 -0.042 0.015 22.222 -2.794 0.027

cg02795305T2 - T3 -0.039 0.015 22.222 -2.614 0.040

cg14344464T1 - T2 0.004 0.019 22.222 0.215 0.975

cg14344464T1 - T3 0.049 0.019 22.222 2.569 0.044

cg14344464T2 - T3 0.045 0.019 22.222 2.354 0.069

cg10273872T1 - T2 -0.027 0.011 22.222 -2.331 0.072

cg10273872T1 - T3 0.015 0.011 22.222 1.344 0.387

cg10273872T2 - T3 0.042 0.011 22.222 3.675 0.004

cg15913725T1 - T2 0.012 0.019 22.222 0.628 0.807

cg15913725T1 - T3 -0.038 0.019 22.222 -2.002 0.135

cg15913725T2 - T3 -0.050 0.019 22.222 -2.630 0.039

cg05357342T1 - T2 0.019 0.018 22.222 1.015 0.575

cg05357342T1 - T3 -0.032 0.018 22.222 -1.758 0.207

cg05357342T2 - T3 -0.051 0.018 22.222 -2.773 0.029

cg12705326T1 - T2 -0.011 0.010 22.222 -1.069 0.542

cg12705326T1 - T3 -0.039 0.010 22.222 -3.877 0.002

cg12705326T2 - T3 -0.028 0.010 22.222 -2.808 0.027

cg05418680T1 - T2 0.017 0.012 22.222 1.429 0.343

cg05418680T1 - T3 -0.016 0.012 22.222 -1.316 0.401

cg05418680T2 - T3 -0.033 0.012 22.222 -2.745 0.030

cg27155460T1 - T2 -0.030 0.025 22.222 -1.194 0.469

cg27155460T1 - T3 0.017 0.025 22.222 0.697 0.768

cg27155460T2 - T3 0.047 0.025 22.222 1.891 0.165

cg09236283T1 - T2 0.026 0.020 22.222 1.315 0.402

cg09236283T1 - T3 -0.045 0.020 22.222 -2.242 0.086

cg09236283T2 - T3 -0.071 0.020 22.222 -3.557 0.005

cg17482360T1 - T2 0.009 0.018 22.222 0.493 0.875

cg17482360T1 - T3 -0.045 0.018 22.222 -2.445 0.057

cg17482360T2 - T3 -0.054 0.018 22.222 -2.938 0.020

cg15545636T1 - T2 0.007 0.010 22.222 0.650 0.794

cg15545636T1 - T3 -0.025 0.010 22.222 -2.458 0.056

cg15545636T2 - T3 -0.032 0.010 22.222 -3.108 0.014

cg02502358T1 - T2 -0.021 0.025 22.222 -0.839 0.683

cg02502358T1 - T3 0.057 0.025 22.222 2.254 0.084

cg02502358T2 - T3 0.078 0.025 22.222 3.093 0.014

cg04326741T1 - T2 0.020 0.017 22.222 1.201 0.465

cg04326741T1 - T3 -0.026 0.017 22.222 -1.526 0.298

cg04326741T2 - T3 -0.046 0.017 22.222 -2.727 0.032

cg19766489T1 - T2 -0.018 0.023 22.222 -0.795 0.710

cg19766489T1 - T3 0.050 0.023 22.222 2.187 0.096

cg19766489T2 - T3 0.069 0.023 22.222 2.981 0.018

cg09073701T1 - T2 -0.004 0.016 22.222 -0.256 0.965

cg09073701T1 - T3 0.039 0.016 22.222 2.417 0.061

cg09073701T2 - T3 0.044 0.016 22.222 2.673 0.035

cg15933979T1 - T2 -0.002 0.016 22.222 -0.126 0.991

cg15933979T1 - T3 0.041 0.016 22.222 2.601 0.041

cg15933979T2 - T3 0.043 0.016 22.222 2.728 0.032

cg22828473T1 - T2 0.013 0.015 22.222 0.848 0.678

cg22828473T1 - T3 -0.020 0.015 22.222 -1.292 0.414

cg22828473T2 - T3 -0.032 0.015 22.222 -2.140 0.104

cg18096774T1 - T2 -0.041 0.017 22.222 -2.349 0.069

cg18096774T1 - T3 0.019 0.017 22.222 1.075 0.539

cg18096774T2 - T3 0.060 0.017 22.222 3.425 0.007

cg23717556T1 - T2 0.037 0.024 22.222 1.536 0.294

cg23717556T1 - T3 -0.049 0.024 22.222 -2.036 0.127

cg23717556T2 - T3 -0.085 0.024 22.222 -3.572 0.005

cg05752892T1 - T2 0.006 0.017 22.222 0.363 0.930

cg05752892T1 - T3 -0.041 0.017 22.222 -2.366 0.067

cg05752892T2 - T3 -0.048 0.017 22.222 -2.730 0.031

cg16066776T1 - T2 -0.017 0.015 22.222 -1.122 0.511

cg16066776T1 - T3 0.016 0.015 22.222 1.080 0.536

cg16066776T2 - T3 0.034 0.015 22.222 2.202 0.093

cg10256477T1 - T2 0.011 0.009 22.222 1.232 0.447

cg10256477T1 - T3 -0.022 0.009 22.222 -2.407 0.062

cg10256477T2 - T3 -0.033 0.009 22.222 -3.640 0.004

cg08315265T1 - T2 0.018 0.016 22.222 1.073 0.540

cg08315265T1 - T3 -0.035 0.016 22.222 -2.099 0.113

cg08315265T2 - T3 -0.052 0.016 22.222 -3.172 0.012

cg01495082T1 - T2 0.014 0.016 22.222 0.852 0.676

cg01495082T1 - T3 -0.042 0.016 22.222 -2.562 0.045

cg01495082T2 - T3 -0.056 0.016 22.222 -3.413 0.007

cg16438246T1 - T2 -0.011 0.012 22.222 -0.877 0.660

cg16438246T1 - T3 -0.036 0.012 22.222 -2.994 0.018

cg16438246T2 - T3 -0.025 0.012 22.222 -2.117 0.109

cg08062858T1 - T2 0.019 0.013 22.222 1.424 0.346

cg08062858T1 - T3 -0.031 0.013 22.222 -2.339 0.071

cg08062858T2 - T3 -0.050 0.013 22.222 -3.763 0.003

cg07856138T1 - T2 -0.014 0.014 22.222 -1.028 0.568

cg07856138T1 - T3 0.032 0.014 22.222 2.330 0.072

cg07856138T2 - T3 0.046 0.014 22.222 3.357 0.008

cg10808013T1 - T2 0.015 0.015 22.222 1.012 0.577

cg10808013T1 - T3 -0.030 0.015 22.222 -2.056 0.122

cg10808013T2 - T3 -0.044 0.015 22.222 -3.068 0.015

cg13660249T1 - T2 0.008 0.019 22.222 0.429 0.904

cg13660249T1 - T3 -0.045 0.019 22.222 -2.436 0.058

cg13660249T2 - T3 -0.053 0.019 22.222 -2.865 0.023

cg09117114T1 - T2 0.012 0.013 22.222 0.876 0.661

cg09117114T1 - T3 -0.036 0.013 22.222 -2.704 0.033

cg09117114T2 - T3 -0.048 0.013 22.222 -3.580 0.005

cg17130450T1 - T2 -0.007 0.014 22.222 -0.516 0.864

cg17130450T1 - T3 -0.039 0.014 22.222 -2.792 0.027

cg17130450T2 - T3 -0.032 0.014 22.222 -2.276 0.080

cg27451732T1 - T2 0.011 0.011 22.222 1.028 0.567

cg27451732T1 - T3 -0.040 0.011 22.222 -3.649 0.004

cg27451732T2 - T3 -0.051 0.011 22.222 -4.677 0.000

cg05042247T1 - T2 -0.002 0.015 22.222 -0.163 0.986

cg05042247T1 - T3 -0.049 0.015 22.222 -3.314 0.008

cg05042247T2 - T3 -0.047 0.015 22.222 -3.151 0.012

cg16030471T1 - T2 0.014 0.015 22.222 0.932 0.626

cg16030471T1 - T3 -0.016 0.015 22.222 -1.028 0.567

cg16030471T2 - T3 -0.030 0.015 22.222 -1.960 0.146

cg23820828T1 - T2 -0.013 0.023 22.222 -0.587 0.828

cg23820828T1 - T3 0.049 0.023 22.222 2.139 0.105

cg23820828T2 - T3 0.062 0.023 22.222 2.727 0.032

cg12408359T1 - T2 0.013 0.009 22.222 1.493 0.313

cg12408359T1 - T3 -0.024 0.009 22.222 -2.745 0.030

cg12408359T2 - T3 -0.037 0.009 22.222 -4.238 0.001

cg21038461T1 - T2 0.017 0.010 22.222 1.684 0.233

cg21038461T1 - T3 -0.024 0.010 22.222 -2.435 0.058

cg21038461T2 - T3 -0.041 0.010 22.222 -4.119 0.001

cg04909225T1 - T2 -0.034 0.023 22.222 -1.501 0.310

cg04909225T1 - T3 0.027 0.023 22.222 1.186 0.474

cg04909225T2 - T3 0.062 0.023 22.222 2.687 0.034

cg04583448T1 - T2 -0.011 0.013 22.222 -0.786 0.715

cg04583448T1 - T3 0.023 0.013 22.222 1.742 0.212

cg04583448T2 - T3 0.034 0.013 22.222 2.528 0.048

cg11961813T1 - T2 0.005 0.011 22.222 0.473 0.885

cg11961813T1 - T3 -0.031 0.011 22.222 -2.768 0.029

cg11961813T2 - T3 -0.037 0.011 22.222 -3.241 0.010

cg24524485T1 - T2 0.018 0.013 22.222 1.399 0.358

cg24524485T1 - T3 -0.014 0.013 22.222 -1.060 0.548

cg24524485T2 - T3 -0.031 0.013 22.222 -2.459 0.056

cg00230282T1 - T2 0.009 0.017 22.222 0.551 0.847

cg00230282T1 - T3 -0.035 0.017 22.222 -2.133 0.106

cg00230282T2 - T3 -0.045 0.017 22.222 -2.684 0.035

cg25495024T1 - T2 -0.007 0.021 22.222 -0.325 0.944

cg25495024T1 - T3 -0.062 0.021 22.222 -2.996 0.017

cg25495024T2 - T3 -0.055 0.021 22.222 -2.671 0.036

cg22863148T1 - T2 0.005 0.010 22.222 0.458 0.891

cg22863148T1 - T3 -0.035 0.010 22.222 -3.478 0.006

cg22863148T2 - T3 -0.039 0.010 22.222 -3.936 0.002

cg00284731T1 - T2 0.014 0.018 22.222 0.783 0.717

cg00284731T1 - T3 -0.034 0.018 22.222 -1.920 0.157

cg00284731T2 - T3 -0.048 0.018 22.222 -2.703 0.033

cg19811994T1 - T2 0.015 0.016 22.222 0.910 0.640

cg19811994T1 - T3 -0.036 0.016 22.222 -2.266 0.082

cg19811994T2 - T3 -0.051 0.016 22.222 -3.176 0.012

cg14157525T1 - T2 0.012 0.013 22.222 0.952 0.614

cg14157525T1 - T3 -0.025 0.013 22.222 -1.990 0.138

cg14157525T2 - T3 -0.037 0.013 22.222 -2.941 0.020

cg10208821T1 - T2 -0.013 0.014 22.222 -0.918 0.635

cg10208821T1 - T3 0.029 0.014 22.222 2.024 0.130

cg10208821T2 - T3 0.042 0.014 22.222 2.942 0.020

cg06070696T1 - T2 -0.009 0.023 22.222 -0.369 0.928

cg06070696T1 - T3 0.037 0.023 22.222 1.581 0.275

cg06070696T2 - T3 0.045 0.023 22.222 1.949 0.149

cg08234270T1 - T2 0.005 0.015 22.222 0.309 0.949

cg08234270T1 - T3 -0.031 0.015 22.222 -1.999 0.136

cg08234270T2 - T3 -0.035 0.015 22.222 -2.308 0.075

cg03097745T1 - T2 0.026 0.011 22.222 2.424 0.060

cg03097745T1 - T3 -0.002 0.011 22.222 -0.164 0.985

cg03097745T2 - T3 -0.027 0.011 22.222 -2.588 0.043

cg07051721T1 - T2 -0.035 0.023 21.249 -1.515 0.304

cg07051721T1 - T3 0.024 0.023 20.422 1.016 0.575

cg07051721T2 - T3 0.059 0.023 21.249 2.547 0.047

cg26413256T1 - T2 0.012 0.020 22.222 0.595 0.824

cg26413256T1 - T3 -0.037 0.020 22.222 -1.893 0.164

cg26413256T2 - T3 -0.049 0.020 22.222 -2.488 0.052

cg07597011T1 - T2 0.009 0.015 22.222 0.621 0.811

cg07597011T1 - T3 -0.034 0.015 22.222 -2.320 0.074

cg07597011T2 - T3 -0.043 0.015 22.222 -2.940 0.020

cg04222410T1 - T2 -0.004 0.011 22.222 -0.401 0.916

cg04222410T1 - T3 -0.033 0.011 22.222 -3.022 0.016

cg04222410T2 - T3 -0.029 0.011 22.222 -2.621 0.040

cg00691874T1 - T2 0.022 0.017 22.222 1.303 0.408

cg00691874T1 - T3 -0.035 0.017 22.222 -2.087 0.115

cg00691874T2 - T3 -0.057 0.017 22.222 -3.390 0.007

cg00485515T1 - T2 0.000 0.012 22.222 -0.018 1.000

cg00485515T1 - T3 -0.033 0.012 22.222 -2.878 0.023

cg00485515T2 - T3 -0.033 0.012 22.222 -2.859 0.024

cg03418847T1 - T2 0.007 0.023 22.222 0.298 0.952

cg03418847T1 - T3 -0.026 0.023 22.222 -1.117 0.514

cg03418847T2 - T3 -0.033 0.023 22.222 -1.414 0.351

cg00643381T1 - T2 0.021 0.016 22.222 1.307 0.406

cg00643381T1 - T3 -0.029 0.016 22.222 -1.794 0.194

cg00643381T2 - T3 -0.050 0.016 22.222 -3.101 0.014

cg27222667T1 - T2 0.005 0.023 22.222 0.217 0.974

cg27222667T1 - T3 -0.044 0.023 22.222 -1.935 0.153

cg27222667T2 - T3 -0.049 0.023 22.222 -2.152 0.102

cg14812743T1 - T2 -0.009 0.012 22.222 -0.790 0.713

cg14812743T1 - T3 0.026 0.012 22.222 2.263 0.082

cg14812743T2 - T3 0.035 0.012 22.222 3.052 0.015

cg26774211T1 - T2 0.043 0.029 22.222 1.492 0.314

cg26774211T1 - T3 -0.052 0.029 22.222 -1.800 0.193

cg26774211T2 - T3 -0.095 0.029 22.222 -3.292 0.009

cg08514594T1 - T2 -0.008 0.009 22.222 -0.931 0.627

cg08514594T1 - T3 -0.039 0.009 22.222 -4.469 0.001

cg08514594T2 - T3 -0.031 0.009 22.222 -3.537 0.005

cg07383420T1 - T2 0.003 0.020 22.222 0.149 0.988

cg07383420T1 - T3 -0.051 0.020 22.222 -2.610 0.041

cg07383420T2 - T3 -0.054 0.020 22.222 -2.759 0.029

cg26105283T1 - T2 -0.003 0.010 22.222 -0.344 0.937

cg26105283T1 - T3 -0.039 0.010 22.222 -3.935 0.002

cg26105283T2 - T3 -0.036 0.010 22.222 -3.590 0.004

cg23683746T1 - T2 0.008 0.013 22.222 0.613 0.815

cg23683746T1 - T3 -0.028 0.013 22.222 -2.153 0.102

cg23683746T2 - T3 -0.036 0.013 22.222 -2.765 0.029

cg15316289T1 - T2 0.017 0.015 22.222 1.112 0.517

cg15316289T1 - T3 -0.018 0.015 22.222 -1.196 0.468

cg15316289T2 - T3 -0.035 0.015 22.222 -2.308 0.075

cg15905979T1 - T2 0.001 0.018 22.222 0.048 0.999

cg15905979T1 - T3 -0.034 0.018 22.222 -1.824 0.185

cg15905979T2 - T3 -0.034 0.018 22.222 -1.873 0.170

cg23086982T1 - T2 -0.017 0.014 22.222 -1.239 0.443

cg23086982T1 - T3 -0.056 0.014 22.222 -4.111 0.001

cg23086982T2 - T3 -0.039 0.014 22.222 -2.872 0.023

cg15438299T1 - T2 0.015 0.015 22.222 1.000 0.585

cg15438299T1 - T3 -0.033 0.015 22.222 -2.152 0.102

cg15438299T2 - T3 -0.048 0.015 22.222 -3.151 0.012

cg04682832T1 - T2 0.010 0.023 22.222 0.438 0.900

cg04682832T1 - T3 0.071 0.023 22.222 3.149 0.012

cg04682832T2 - T3 0.061 0.023 22.222 2.710 0.033

cg18106582T1 - T2 -0.001 0.016 22.222 -0.070 0.997

cg18106582T1 - T3 -0.042 0.016 22.222 -2.566 0.045

cg18106582T2 - T3 -0.041 0.016 22.222 -2.496 0.051

cg16235962T1 - T2 0.018 0.012 22.222 1.518 0.302

cg16235962T1 - T3 -0.027 0.012 22.222 -2.258 0.083

cg16235962T2 - T3 -0.045 0.012 22.222 -3.776 0.003

cg14232793T1 - T2 0.018 0.009 22.222 2.155 0.102

cg14232793T1 - T3 -0.010 0.009 22.222 -1.160 0.488

cg14232793T2 - T3 -0.028 0.009 22.222 -3.316 0.008

cg07593523T1 - T2 -0.016 0.014 22.222 -1.138 0.502

cg07593523T1 - T3 0.023 0.014 22.222 1.650 0.246

cg07593523T2 - T3 0.039 0.014 22.222 2.788 0.028

cg03040736T1 - T2 0.001 0.021 22.222 0.050 0.999

cg03040736T1 - T3 -0.064 0.021 22.222 -3.074 0.015

cg03040736T2 - T3 -0.065 0.021 22.222 -3.124 0.013

cg23598132T1 - T2 -0.017 0.011 22.222 -1.486 0.316

cg23598132T1 - T3 0.020 0.011 22.222 1.823 0.185

cg23598132T2 - T3 0.037 0.011 22.222 3.309 0.009

cg18025409T1 - T2 -0.012 0.019 22.222 -0.622 0.809

cg18025409T1 - T3 -0.059 0.019 22.222 -3.172 0.012

cg18025409T2 - T3 -0.048 0.019 22.222 -2.550 0.046

cg05226443T1 - T2 0.014 0.012 22.222 1.177 0.479

cg05226443T1 - T3 -0.018 0.012 22.222 -1.514 0.304

cg05226443T2 - T3 -0.032 0.012 22.222 -2.691 0.034

cg23250700T1 - T2 0.028 0.018 22.222 1.518 0.302

cg23250700T1 - T3 -0.044 0.018 22.222 -2.379 0.065

cg23250700T2 - T3 -0.072 0.018 22.222 -3.897 0.002

cg05718389T1 - T2 0.001 0.082 22.222 0.011 1.000

cg05718389T1 - T3 -0.199 0.082 22.222 -2.410 0.061

cg05718389T2 - T3 -0.200 0.082 22.222 -2.422 0.060

cg11298010T1 - T2 -0.018 0.017 22.222 -1.105 0.521

cg11298010T1 - T3 0.031 0.017 22.222 1.843 0.179

cg11298010T2 - T3 0.049 0.017 22.222 2.948 0.019

cg05586172T1 - T2 0.016 0.014 22.222 1.183 0.475

cg05586172T1 - T3 -0.029 0.014 22.222 -2.146 0.103

cg05586172T2 - T3 -0.046 0.014 22.222 -3.329 0.008

cg17890684T1 - T2 -0.005 0.011 22.222 -0.432 0.903

cg17890684T1 - T3 -0.036 0.011 22.222 -3.356 0.008

cg17890684T2 - T3 -0.032 0.011 22.222 -2.924 0.020

cg13176235T1 - T2 0.010 0.018 22.222 0.548 0.848

cg13176235T1 - T3 0.039 0.018 22.222 2.212 0.091

cg13176235T2 - T3 0.029 0.018 22.222 1.664 0.241

cg12032655T1 - T2 -0.011 0.022 22.222 -0.518 0.863

cg12032655T1 - T3 0.030 0.022 22.222 1.365 0.376

cg12032655T2 - T3 0.042 0.022 22.222 1.883 0.167

cg27589676T1 - T2 0.020 0.015 22.222 1.344 0.387

cg27589676T1 - T3 0.056 0.015 22.222 3.756 0.003

cg27589676T2 - T3 0.036 0.015 22.222 2.412 0.061

cg27149441T1 - T2 0.001 0.013 22.222 0.073 0.997

cg27149441T1 - T3 -0.041 0.013 22.222 -3.255 0.010

cg27149441T2 - T3 -0.042 0.013 22.222 -3.328 0.008

cg13804534T1 - T2 -0.009 0.026 22.222 -0.347 0.936

cg13804534T1 - T3 -0.090 0.026 22.222 -3.388 0.007

cg13804534T2 - T3 -0.080 0.026 22.222 -3.041 0.016

cg17459219T1 - T2 0.019 0.023 22.222 0.836 0.685

cg17459219T1 - T3 -0.021 0.023 22.222 -0.933 0.626

cg17459219T2 - T3 -0.040 0.023 22.222 -1.769 0.203

cg22887461T1 - T2 0.009 0.013 22.222 0.652 0.793

cg22887461T1 - T3 -0.029 0.013 22.222 -2.150 0.103

cg22887461T2 - T3 -0.037 0.013 22.222 -2.802 0.027

cg04780393T1 - T2 0.006 0.012 22.222 0.472 0.885

cg04780393T1 - T3 -0.031 0.012 22.222 -2.512 0.050

cg04780393T2 - T3 -0.037 0.012 22.222 -2.984 0.018

cg01875106T1 - T2 0.006 0.017 22.222 0.362 0.931

cg01875106T1 - T3 -0.045 0.017 22.222 -2.588 0.043

cg01875106T2 - T3 -0.051 0.017 22.222 -2.949 0.019

cg22085190T1 - T2 0.004 0.010 22.222 0.405 0.914

cg22085190T1 - T3 -0.029 0.010 22.222 -2.930 0.020

cg22085190T2 - T3 -0.033 0.010 22.222 -3.336 0.008

cg23648661T1 - T2 0.048 0.022 21.222 2.210 0.092

cg23648661T1 - T3 -0.016 0.022 21.494 -0.704 0.764

cg23648661T2 - T3 -0.063 0.022 21.494 -2.830 0.026

cg24931825T1 - T2 0.012 0.022 22.222 0.531 0.857

cg24931825T1 - T3 -0.026 0.022 22.222 -1.187 0.473

cg24931825T2 - T3 -0.037 0.022 22.222 -1.717 0.221

cg20703579T1 - T2 0.025 0.011 22.222 2.239 0.086

cg20703579T1 - T3 -0.022 0.011 22.222 -1.956 0.147

cg20703579T2 - T3 -0.047 0.011 22.222 -4.195 0.001

cg23089508T1 - T2 0.033 0.025 22.222 1.332 0.393

cg23089508T1 - T3 -0.023 0.025 22.222 -0.939 0.622

cg23089508T2 - T3 -0.056 0.025 22.222 -2.271 0.081

cg13250324T1 - T2 0.009 0.017 22.222 0.525 0.860

cg13250324T1 - T3 -0.027 0.017 22.222 -1.589 0.271

cg13250324T2 - T3 -0.036 0.017 22.222 -2.114 0.110

cg13971484T1 - T2 0.015 0.014 22.222 1.096 0.526

cg13971484T1 - T3 -0.023 0.014 22.222 -1.706 0.225

cg13971484T2 - T3 -0.038 0.014 22.222 -2.802 0.027

cg14956201T1 - T2 -0.017 0.016 22.222 -1.084 0.533

cg14956201T1 - T3 0.040 0.016 22.222 2.492 0.052

cg14956201T2 - T3 0.057 0.016 22.222 3.577 0.005

cg04981737T1 - T2 -0.003 0.011 22.222 -0.282 0.957

cg04981737T1 - T3 0.044 0.011 22.222 3.903 0.002

cg04981737T2 - T3 0.048 0.011 22.222 4.185 0.001

cg25135333T1 - T2 0.011 0.015 22.222 0.727 0.750

cg25135333T1 - T3 -0.033 0.015 22.222 -2.158 0.101

cg25135333T2 - T3 -0.043 0.015 22.222 -2.885 0.022

cg06885616T1 - T2 0.013 0.016 22.222 0.813 0.699

cg06885616T1 - T3 -0.045 0.016 22.222 -2.885 0.022

cg06885616T2 - T3 -0.058 0.016 22.222 -3.698 0.003

cg25577598T1 - T2 0.006 0.114 22.222 0.051 0.999

cg25577598T1 - T3 0.329 0.114 22.222 2.888 0.022

cg25577598T2 - T3 0.324 0.114 22.222 2.837 0.025

cg02683627T1 - T2 -0.002 0.019 22.222 -0.084 0.996

cg02683627T1 - T3 -0.053 0.019 22.222 -2.730 0.031

cg02683627T2 - T3 -0.051 0.019 22.222 -2.647 0.038

cg26667946T1 - T2 -0.007 0.016 22.222 -0.427 0.905

cg26667946T1 - T3 -0.038 0.016 22.222 -2.322 0.073

cg26667946T2 - T3 -0.031 0.016 22.222 -1.895 0.164

cg11314536T1 - T2 -0.007 0.013 22.222 -0.555 0.845

cg11314536T1 - T3 0.032 0.013 22.222 2.407 0.062

cg11314536T2 - T3 0.039 0.013 22.222 2.963 0.019

cg08102026T1 - T2 0.029 0.023 22.222 1.242 0.442

cg08102026T1 - T3 -0.042 0.023 22.222 -1.815 0.188

cg08102026T2 - T3 -0.071 0.023 22.222 -3.057 0.015

cg08678044T1 - T2 -0.009 0.014 22.222 -0.623 0.809

cg08678044T1 - T3 0.032 0.014 22.222 2.209 0.092

cg08678044T2 - T3 0.041 0.014 22.222 2.832 0.025

cg24740850T1 - T2 0.015 0.013 22.222 1.181 0.477

cg24740850T1 - T3 -0.026 0.013 22.222 -2.006 0.134

cg24740850T2 - T3 -0.042 0.013 22.222 -3.187 0.011

cg04532638T1 - T2 0.001 0.033 22.222 0.034 0.999

cg04532638T1 - T3 -0.090 0.033 22.222 -2.745 0.030

cg04532638T2 - T3 -0.091 0.033 22.222 -2.779 0.028

cg14797136T1 - T2 -0.013 0.014 22.222 -0.953 0.614

cg14797136T1 - T3 0.017 0.014 22.222 1.228 0.450

cg14797136T2 - T3 0.031 0.014 22.222 2.181 0.097

cg01308409T1 - T2 -0.009 0.016 22.222 -0.588 0.828

cg01308409T1 - T3 0.034 0.016 22.222 2.141 0.104

cg01308409T2 - T3 0.043 0.016 22.222 2.729 0.031

cg21995472T1 - T2 -0.007 0.015 22.222 -0.463 0.889

cg21995472T1 - T3 0.036 0.015 22.222 2.307 0.076

cg21995472T2 - T3 0.043 0.015 22.222 2.770 0.029

cg25073700T1 - T2 0.029 0.012 22.222 2.429 0.059

cg25073700T1 - T3 -0.017 0.012 22.222 -1.433 0.341

cg25073700T2 - T3 -0.047 0.012 22.222 -3.862 0.002

cg24106132T1 - T2 -0.045 0.020 22.222 -2.205 0.092

cg24106132T1 - T3 -0.018 0.020 22.222 -0.880 0.658

cg24106132T2 - T3 0.027 0.020 22.222 1.325 0.397

cg16715082T1 - T2 0.017 0.041 22.222 0.400 0.916

cg16715082T1 - T3 -0.118 0.041 22.222 -2.848 0.024

cg16715082T2 - T3 -0.134 0.041 22.222 -3.247 0.010

cg23124182T1 - T2 -0.012 0.016 22.222 -0.774 0.723

cg23124182T1 - T3 0.025 0.016 22.222 1.576 0.277

cg23124182T2 - T3 0.037 0.016 22.222 2.350 0.069

cg03418876T1 - T2 0.018 0.020 22.222 0.932 0.626

cg03418876T1 - T3 -0.043 0.020 22.222 -2.174 0.098

cg03418876T2 - T3 -0.061 0.020 22.222 -3.107 0.014

cg12528240T1 - T2 0.002 0.019 22.222 0.131 0.991

cg12528240T1 - T3 -0.043 0.019 22.222 -2.315 0.074

cg12528240T2 - T3 -0.046 0.019 22.222 -2.446 0.057

cg12358432T1 - T2 0.034 0.010 22.222 3.358 0.008

cg12358432T1 - T3 -0.008 0.010 22.222 -0.842 0.681

cg12358432T2 - T3 -0.042 0.010 22.222 -4.200 0.001

cg11735008T1 - T2 0.031 0.016 22.222 1.906 0.160

cg11735008T1 - T3 -0.010 0.016 22.222 -0.582 0.831

cg11735008T2 - T3 -0.041 0.016 22.222 -2.488 0.052

cg07834691T1 - T2 -0.005 0.016 22.222 -0.329 0.942

cg07834691T1 - T3 -0.068 0.016 22.222 -4.191 0.001

cg07834691T2 - T3 -0.063 0.016 22.222 -3.862 0.002

cg15964611T1 - T2 0.005 0.013 22.222 0.382 0.923

cg15964611T1 - T3 0.036 0.013 22.222 2.775 0.028

cg15964611T2 - T3 0.031 0.013 22.222 2.394 0.063

cg12824286T1 - T2 0.025 0.015 22.222 1.659 0.243

cg12824286T1 - T3 -0.031 0.015 22.222 -2.053 0.123

cg12824286T2 - T3 -0.056 0.015 22.222 -3.712 0.003

cg12913956T1 - T2 0.000 0.013 22.222 0.030 1.000

cg12913956T1 - T3 -0.035 0.013 22.222 -2.714 0.033

cg12913956T2 - T3 -0.036 0.013 22.222 -2.744 0.030

cg09714724T1 - T2 0.011 0.012 22.222 0.878 0.659

cg09714724T1 - T3 -0.030 0.012 22.222 -2.400 0.063

cg09714724T2 - T3 -0.041 0.012 22.222 -3.279 0.009

cg22774937T1 - T2 -0.009 0.020 22.222 -0.423 0.906

cg22774937T1 - T3 0.044 0.020 22.222 2.170 0.099

cg22774937T2 - T3 0.053 0.020 22.222 2.593 0.042

cg00776381T1 - T2 -0.032 0.027 22.222 -1.158 0.490

cg00776381T1 - T3 0.012 0.027 22.222 0.446 0.897

cg00776381T2 - T3 0.044 0.027 22.222 1.604 0.265

cg18072282T1 - T2 -0.016 0.024 22.222 -0.690 0.772

cg18072282T1 - T3 0.037 0.024 22.222 1.559 0.284

cg18072282T2 - T3 0.054 0.024 22.222 2.249 0.085

cg05125809T1 - T2 -0.005 0.017 22.222 -0.290 0.955

cg05125809T1 - T3 0.037 0.017 22.222 2.246 0.085

cg05125809T2 - T3 0.042 0.017 22.222 2.536 0.047

cg19725972T1 - T2 -0.008 0.011 22.222 -0.695 0.769

cg19725972T1 - T3 0.025 0.011 22.222 2.258 0.083

cg19725972T2 - T3 0.033 0.011 22.222 2.953 0.019

cg27370618T1 - T2 -0.008 0.016 22.222 -0.501 0.871

cg27370618T1 - T3 0.033 0.016 22.222 2.148 0.103

cg27370618T2 - T3 0.041 0.016 22.222 2.649 0.037

cg15922024T1 - T2 0.010 0.011 22.222 0.899 0.647

cg15922024T1 - T3 -0.028 0.011 22.222 -2.635 0.038

cg15922024T2 - T3 -0.038 0.011 22.222 -3.534 0.005

cg07710391T1 - T2 0.040 0.037 20.452 1.083 0.535

cg07710391T1 - T3 -0.072 0.039 20.734 -1.857 0.176

cg07710391T2 - T3 -0.113 0.037 20.452 -3.015 0.018

cg27536425T1 - T2 -0.007 0.018 22.222 -0.392 0.919

cg27536425T1 - T3 -0.051 0.018 22.222 -2.803 0.027

cg27536425T2 - T3 -0.044 0.018 22.222 -2.411 0.061

cg02857557T1 - T2 -0.003 0.014 22.222 -0.204 0.977

cg02857557T1 - T3 -0.039 0.014 22.222 -2.785 0.028

cg02857557T2 - T3 -0.036 0.014 22.222 -2.582 0.043

cg08260766T1 - T2 0.005 0.024 22.222 0.192 0.980

cg08260766T1 - T3 -0.051 0.024 22.222 -2.098 0.113

cg08260766T2 - T3 -0.056 0.024 22.222 -2.290 0.078

cg18816605T1 - T2 0.018 0.016 22.222 1.132 0.505

cg18816605T1 - T3 -0.028 0.016 22.222 -1.761 0.206

cg18816605T2 - T3 -0.046 0.016 22.222 -2.893 0.022

cg23442853T1 - T2 -0.009 0.020 22.222 -0.433 0.902

cg23442853T1 - T3 0.045 0.020 22.222 2.212 0.091

cg23442853T2 - T3 0.054 0.020 22.222 2.645 0.038

cg22632576T1 - T2 0.005 0.014 22.222 0.366 0.929

cg22632576T1 - T3 -0.045 0.014 22.222 -3.251 0.010

cg22632576T2 - T3 -0.050 0.014 22.222 -3.617 0.004

cg17408816T1 - T2 -0.003 0.021 22.222 -0.152 0.987

cg17408816T1 - T3 0.048 0.021 22.222 2.312 0.075

cg17408816T2 - T3 0.051 0.021 22.222 2.464 0.055

cg04055963T1 - T2 0.045 0.027 22.222 1.641 0.250

cg04055963T1 - T3 -0.040 0.027 22.222 -1.457 0.330

cg04055963T2 - T3 -0.085 0.027 22.222 -3.099 0.014

cg20298448T1 - T2 0.003 0.019 22.222 0.142 0.989

cg20298448T1 - T3 -0.055 0.019 22.222 -2.866 0.023

cg20298448T2 - T3 -0.057 0.019 22.222 -3.008 0.017

cg17537449T1 - T2 0.001 0.011 22.222 0.094 0.995

cg17537449T1 - T3 0.036 0.011 22.222 3.233 0.010

cg17537449T2 - T3 0.035 0.011 22.222 3.139 0.013

cg21030867T1 - T2 0.023 0.017 22.222 1.363 0.377

cg21030867T1 - T3 -0.047 0.017 22.222 -2.825 0.025

cg21030867T2 - T3 -0.070 0.017 22.222 -4.189 0.001

cg00490852T1 - T2 0.002 0.012 22.222 0.132 0.990

cg00490852T1 - T3 -0.035 0.012 22.222 -2.891 0.022

cg00490852T2 - T3 -0.037 0.012 22.222 -3.024 0.016

cg13074532T1 - T2 0.011 0.014 22.222 0.806 0.703

cg13074532T1 - T3 -0.022 0.014 22.222 -1.637 0.251

cg13074532T2 - T3 -0.033 0.014 22.222 -2.443 0.057

cg25686080T1 - T2 0.007 0.008 22.222 0.826 0.691

cg25686080T1 - T3 -0.025 0.008 22.222 -3.093 0.014

cg25686080T2 - T3 -0.032 0.008 22.222 -3.920 0.002

cg09443388T1 - T2 -0.006 0.020 22.222 -0.308 0.949

cg09443388T1 - T3 -0.047 0.020 22.222 -2.270 0.081

cg09443388T2 - T3 -0.040 0.020 22.222 -1.962 0.145

cg03583625T1 - T2 -0.005 0.012 22.222 -0.391 0.919

cg03583625T1 - T3 0.036 0.012 22.222 2.931 0.020

cg03583625T2 - T3 0.041 0.012 22.222 3.322 0.008

cg08134805T1 - T2 0.018 0.015 22.222 1.190 0.471

cg08134805T1 - T3 -0.019 0.015 22.222 -1.295 0.413

cg08134805T2 - T3 -0.037 0.015 22.222 -2.485 0.053

cg02942158T1 - T2 -0.022 0.025 22.222 -0.869 0.665

cg02942158T1 - T3 0.050 0.025 22.222 1.985 0.139

cg02942158T2 - T3 0.072 0.025 22.222 2.854 0.024

cg16535018T1 - T2 0.005 0.011 22.222 0.455 0.893

cg16535018T1 - T3 0.037 0.011 22.222 3.416 0.007

cg16535018T2 - T3 0.032 0.011 22.222 2.961 0.019

cg07618581T1 - T2 0.006 0.013 22.222 0.431 0.903

cg07618581T1 - T3 -0.027 0.013 22.222 -2.081 0.117

cg07618581T2 - T3 -0.032 0.013 22.222 -2.512 0.050

cg05506480T1 - T2 -0.021 0.019 22.222 -1.102 0.523

cg05506480T1 - T3 0.033 0.019 22.222 1.768 0.203

cg05506480T2 - T3 0.054 0.019 22.222 2.870 0.023

cg19542346T1 - T2 0.003 0.015 22.222 0.236 0.970

cg19542346T1 - T3 -0.034 0.015 22.222 -2.311 0.075

cg19542346T2 - T3 -0.037 0.015 22.222 -2.547 0.046

cg07753073T1 - T2 -0.019 0.022 22.222 -0.892 0.651

cg07753073T1 - T3 0.036 0.022 22.222 1.648 0.247

cg07753073T2 - T3 0.055 0.022 22.222 2.539 0.047

cg23792133T1 - T2 0.018 0.015 22.222 1.195 0.468

cg23792133T1 - T3 -0.018 0.015 22.222 -1.197 0.467

cg23792133T2 - T3 -0.037 0.015 22.222 -2.392 0.064

cg23013402T1 - T2 0.017 0.012 22.222 1.418 0.349

cg23013402T1 - T3 -0.015 0.012 22.222 -1.236 0.445

cg23013402T2 - T3 -0.032 0.012 22.222 -2.654 0.037

cg07824172T1 - T2 -0.002 0.011 22.222 -0.151 0.987

cg07824172T1 - T3 -0.048 0.011 22.222 -4.301 0.001

cg07824172T2 - T3 -0.047 0.011 22.222 -4.149 0.001

cg10871345T1 - T2 0.012 0.016 22.222 0.750 0.737

cg10871345T1 - T3 -0.032 0.016 22.222 -1.945 0.150

cg10871345T2 - T3 -0.044 0.016 22.222 -2.695 0.034

cg08239694T1 - T2 0.006 0.017 22.222 0.330 0.942

cg08239694T1 - T3 -0.050 0.017 22.222 -2.881 0.023

cg08239694T2 - T3 -0.055 0.017 22.222 -3.211 0.011

cg04826488T1 - T2 0.009 0.022 22.222 0.396 0.918

cg04826488T1 - T3 -0.073 0.022 22.222 -3.326 0.008

cg04826488T2 - T3 -0.082 0.022 22.222 -3.722 0.003

cg24010993T1 - T2 0.024 0.012 22.222 1.958 0.146

cg24010993T1 - T3 -0.021 0.012 22.222 -1.718 0.221

cg24010993T2 - T3 -0.044 0.012 22.222 -3.676 0.004

cg27420135T1 - T2 -0.005 0.012 22.222 -0.412 0.911

cg27420135T1 - T3 0.029 0.012 22.222 2.362 0.068

cg27420135T2 - T3 0.035 0.012 22.222 2.774 0.029

cg02939577T1 - T2 0.009 0.016 22.222 0.596 0.824

cg02939577T1 - T3 -0.032 0.016 22.222 -2.010 0.133

cg02939577T2 - T3 -0.041 0.016 22.222 -2.606 0.041

cg20306355T1 - T2 0.004 0.014 22.222 0.243 0.968

cg20306355T1 - T3 0.037 0.014 22.222 2.541 0.047

cg20306355T2 - T3 0.033 0.014 22.222 2.297 0.077

cg21580007T1 - T2 -0.008 0.017 22.222 -0.478 0.882

cg21580007T1 - T3 -0.058 0.017 22.222 -3.344 0.008

cg21580007T2 - T3 -0.050 0.017 22.222 -2.866 0.023

cg18374914T1 - T2 0.015 0.013 22.222 1.153 0.493

cg18374914T1 - T3 -0.016 0.013 22.222 -1.308 0.406

cg18374914T2 - T3 -0.031 0.013 22.222 -2.460 0.055

cg10938393T1 - T2 0.032 0.018 22.222 1.764 0.205

cg10938393T1 - T3 -0.023 0.018 22.222 -1.286 0.418

cg10938393T2 - T3 -0.055 0.018 22.222 -3.050 0.015

cg12419669T1 - T2 -0.007 0.010 22.222 -0.685 0.775

cg12419669T1 - T3 -0.041 0.010 22.222 -3.912 0.002

cg12419669T2 - T3 -0.033 0.010 22.222 -3.228 0.010

cg02370644T1 - T2 0.008 0.011 22.222 0.736 0.745

cg02370644T1 - T3 -0.023 0.011 22.222 -2.040 0.126

cg02370644T2 - T3 -0.031 0.011 22.222 -2.775 0.028

cg13201279T1 - T2 -0.007 0.015 22.222 -0.492 0.876

cg13201279T1 - T3 0.029 0.015 22.222 1.904 0.161

cg13201279T2 - T3 0.036 0.015 22.222 2.396 0.063

cg22711720T1 - T2 0.003 0.011 22.222 0.305 0.950

cg22711720T1 - T3 -0.033 0.011 22.222 -2.893 0.022

cg22711720T2 - T3 -0.036 0.011 22.222 -3.198 0.011

cg17115737T1 - T2 -0.004 0.015 22.222 -0.258 0.964

cg17115737T1 - T3 0.053 0.015 22.222 3.654 0.004

cg17115737T2 - T3 0.057 0.015 22.222 3.912 0.002
