# Supplementary material for: Longitudinal peripheral DNA methylation remodeling following ketamine-assisted psychotherapy in major depressive disorder
Source: Epigenetics. 2026 Oct 15;21(1):2731659. doi: 10.1080/15592294.2026.2731659 (PMC13679325; doi:10.1080/15592294.2026.2731659)
Supplement: Supplementary file 2 [file KEPI_A_2731659_SM0052.docx]

**Supplementary Full Methods**

**Participants**: This study was approved by the University of Ottawa Research Ethics Board UOREB#: H-03-23-8998. Prior to participation, individuals were provided with a detailed overview of the study's objectives, procedures, potential risks, and benefits, and informed consent was obtained. Data were collected from 10 adults diagnosed with Major Depressive Disorder (MDD) seeking outpatient therapy and 12 healthy controls. Participants with MDD were recruited from the Inside Out Integrative Psychological and Holistic Wellness Clinic located in Ottawa. Control participants were community volunteers with no current psychiatric disorders. All participants were fluent in English and able to complete self-report measures independently.

**Control Participants**. Twelve healthy adults with no current psychiatric disorders were recruited as a comparison group. Controls provided two biological samples collected at two time points separated by a two-week interval. Their methylation data were processed together with the MDD group to ensure uniform normalization and probe filtering and were used only for quality-control and annotation purposes. No group-level statistical comparisons between controls and MDD participants were performed for this study.

**Probe Filtering and Statistical Output:**

Probes with more than 10% missing β values were excluded from analysis. Following preprocessing and quality control using the SeSAMe QCDPB workflow, 802,595 CpG loci were included in the downstream analysis. Loci with a nominal p-value below .05 for the MDD T3-versus-T2 comparison were retained, yielding an initial set of 20,770 CpG loci. This nominal threshold was used as an exploratory screening criterion for subsequent magnitude and baseline-stability filtering. The T1 assessment was not included in the initial significance screen but was used in later calculations of pre-treatment fluctuation and maximum methylation change

**Primary Probe-Level Screening**

For the primary probe-level analysis, methylation β-values were matched within participants between the immediate pre-treatment assessment (T2; Pool_ID “b”) and the post-treatment assessment (T3; Pool_ID “c”). At each CpG locus, a paired t-test was used to compare T2 and T3 methylation levels across the 10 participants with complete data. CpG loci with a nominal p-value below .05 were retained as an exploratory candidate pool for subsequent magnitude and baseline-stability filtering. The nominal p-values were not interpreted as evidence of epigenome-wide statistical significance.

**Statistical Analysis:**

Downstream analyses were restricted to the 10 MDD participants. The probe-level dataset contained mean methylation β-values at T1, T2, and T3 together with the nominal p-value for the MDD T3-versus-T2 comparison. For each CpG locus, pre-treatment fluctuation was calculated as T2 − T1, treatment-associated change as T3 − T2, and change from the first baseline as T3 − T1. These values were used to calculate the maximum absolute methylation change and the stability ratio applied in the subsequent filtering procedure.

**Linear Mixed-Effects Analysis**

As a supplementary sensitivity analysis, longitudinal methylation changes were evaluated across the 603 gene-annotated loci entering stability classification. Sample-level metadata were matched to methylation arrays using Sentrix ID and position, and replicate samples were removed. All 10 MDD participants contributed complete methylation measurements at T1, T2, and T3. For each CpG locus, β-values were reshaped into long format and analyzed using the lmer function in the lme4 package, with timepoint included as a fixed effect and participant included as a random intercept to account for repeated measurements. Estimated marginal means and pairwise contrasts for T1–T2, T1–T3, and T2–T3 were calculated using the emmeans package. The T3–T2 contrast was used to assess treatment-associated methylation change. Because these loci had been predefined through the primary filtering analysis, the mixed-effects models were treated as a supplementary sensitivity analysis.

**Quantitative Filtering of CpG Loci:**

To identify biologically meaningful loci, a magnitude filter was applied to the 20,770 CpG loci meeting the nominal T3-versus-T2 screening criterion. For each locus, the maximum absolute methylation change was calculated as Δmax = max (|T3 − T1|, |T3 − T2|). CpG loci were retained when Δmax ≥ .035, reducing the set to 1,180 loci. Gene annotation was not used as a quantitative selection criterion. Loci without available gene annotations remained in the complete probe-level analysis but were excluded from subsequent gene-based functional interpretation.

Following magnitude filtering, the 1,180 loci were evaluated quantitatively before gene-based interpretation. Gene annotation and enrichment analyses were conducted only after completion of the stability-filtering procedure and did not influence locus selection, retention, or prioritization.

Following magnitude filtering, treatment-associated change was evaluated relative to baseline fluctuation using a stability ratio, defined as R = |T3 − T2| / |T2 − T1|. Loci with treatment-associated movement at least three times greater than baseline fluctuation were classified as Baseline Stable Treatment-Responsive loci (BSTR; R ≥ 3; n = 692). Loci with treatment-associated movement at least twice, but less than three times, the magnitude of baseline fluctuation were retained as Dynamic Movers (2 ≤ R < 3; n = 270). Loci with R < 2 were excluded (n = 218). This procedure yielded 962 retained CpG loci. Among the 1,180 magnitude-filtered loci, 603 had available gene annotations, including 359 BSTR loci, 158 Dynamic Movers, and 86 excluded loci. The final gene-annotated treatment-associated signature therefore comprised 517 retained CpG loci.

**Functional Enrichment Analysis**

Exploratory functional enrichment analysis was performed using g:Profiler on the unique gene symbols mapped from all 603 gene-annotated magnitude-filtered loci. The full set was analyzed rather than a manually selected subset to minimize pathway-selection bias. Because 86 of these loci did not meet the final stability criterion, the enrichment analysis was interpreted as descriptive of the broader magnitude-filtered methylation set rather than the final retained 517-locus signature. Enrichment results were not used for locus selection, retention, or prioritization.

**Screening and Diagnostic Assessment:** All participants completed a comprehensive clinical interview conducted by a licensed clinician. For the MDD group, diagnoses were established using the Mini International Neuropsychiatric Interview (MINI 7.0.2; Sheehan et al., 2016) according to DSM-5 criteria. Participants were excluded if they reported a history of psychosis, schizophrenia, bipolar disorder, neurological or organic brain disease, or any condition that would interfere with the safe administration of ketamine or interpretation of psychological measures.

**Survey Administration:** All participants completed the psychological measures online using a secure, encrypted platform, REDCap database. The survey battery included standardized questionnaires assessing depressive symptoms, personality traits, and childhood maltreatment. Participants were asked to complete the demographics form and three questionnaires independently in a quiet, distraction-free environment.

**Demographics Form** (Canada) is a 25 item self-report questionnaire that was used to collect participants’ demographics information including, age, gender, ethnicity, educational background, socioeconomic status and other relevant personal characteristics.

**Depression Measure:** Depressive symptoms were assessed using the Beck Depression Inventory–II (BDI-II; Beck et al., 1996), a 21-item self-report questionnaire widely used to evaluate the severity of depressive symptoms across cognitive, emotional, and somatic domains. Scores were calculated according to standardized guidelines, with higher scores reflecting more severe depressive symptoms. The BDI-II was administered at all three timepoints (T_1_, T_2_, and T_3_) to evaluate symptom stability at baseline and change following ketamine-assisted psychotherapy.

**Childhood Trauma Questionnaire** (CTQ; Bernstein et al., 2003) was employed to evaluate experiences of early-life stress. This self-report instrument includes 28 items, each rated on a five-point scale ranging from 1 (Never True) to 5 (Very Often True), and assesses the frequency of different types of childhood maltreatment, specifically physical, emotional, and sexual abuse, as well as physical and emotional neglect.

**Personality Measure:** Personality traits were measured using the NEO Five-Factor Inventory–3 (NEO-FFI-3; McCrae & Costa, 2010), consists of 60 self-descriptive statements rated on a five-point Likert scale which assesses the five major domains of personality: Neuroticism, Extraversion, Openness, Agreeableness, and Conscientiousness. The NEO-FFI-3 was administered at each timepoint to determine whether ketamine treatment was associated with changes in stable dispositional traits.

**Ketamine-Assisted Psychotherapy Procedure:** Participants in the MDD treatment cohort received intranasal racemic ketamine under physician supervision as part of a standardized ketamine-assisted psychotherapy (KAP) protocol delivered at Field Trip Health. The full KAP intervention consisted of three ketamine dosing sessions per participant, delivered over a total treatment period of approximately seven weeks, with sessions scheduled approximately **every two weeks**. Each dosing session was embedded within a structured psychotherapy framework that included preparatory psychotherapy, guided ketamine administration, and post-session integration therapy.

Ten participants (7 female, 3 male) completed the full KAP protocol. All participants received three intranasal ketamine dosing sessions. The initial dosing session (Session 1) followed a standardized starting dose of 180 mg intranasal ketamine (four sprays). Doses were subsequently titrated in Sessions 2 and 3 to a range of 225–270 mg, based on individual clinical response and tolerability, in accordance with the clinic’s established dosing guidelines. No additional ketamine dosing sessions occurred beyond these three protocolized administrations.

Adverse effects were generally mild and transient, most commonly including nausea, dizziness, or headache. For participants with a known history of nausea during psychedelic-assisted treatments, prophylactic ondansetron (8 mg) was administered at the clinician’s discretion. No serious adverse events were reported. All ketamine-assisted psychotherapy sessions were completed prior to the final psychological and biological assessment (T_3_). Healthy control participants did not receive ketamine or psychotherapy interventions.

**Biological Sampling**: Buccal swab samples were collected from participants with MDD using sterile DNA Genotek OCR-100 kits (DNA Genotek, Ottawa, Canada), which are designed to yield sufficient DNA (≥500 ng) for downstream bisulfite conversion and genome-wide DNA methylation analysis. Biological samples were collected at three predefined timepoints: T_1_ (baseline, two weeks prior to the first ketamine dosing session), T_2_ (baseline pre-treatment, collected immediately prior to the initial ketamine dosing session), and T_3_ (post-treatment, collected following completion of all three ketamine dosing sessions).

The total interval between the first buccal swab (T_1_) and the final buccal swab (T_3_) corresponded to the full ketamine-assisted psychotherapy treatment period and wa**s** approximately seven weeks**,** reflecting the two-week pre-treatment baseline interval followed by three dosing sessions delivered approximately every two weeks. All biological sampling was completed prior to data analysis. Samples were processed and stored according to the manufacturer’s instructions and shipped to the University of British Columbia (UBC) for genome-wide methylation analysis.

**DNA Extraction**: Genomic DNA was extracted from swab samples using standard column-based purification protocols. All extractions were performed at the University of British Columbia (UBC) following established procedures to ensure high-quality DNA suitable for downstream DNA methylation analysis.

**Bisulfite Conversion:** Bisulfite conversion of genomic DNA was conducted using the Zymo EZ DNA Methylation Kit, which converts unmethylated cytosines to uracil while leaving methylated cytosines unchanged. This step enables quantitative measurement of methylation at CpG sites using array-based methods.

**DNA Methylation Quantification**: Genome-wide DNA methylation was measured using the Illumina MethylationEPIC v2.0 BeadChip, which interrogates over 935,000 CpG sites across the genome. Samples were processed and scanned according to the manufacturer’s instructions at UBC’s epigenetics laboratory. The EPIC v2.0 platform provides quantitative estimates of methylation levels (β values) at each CpG site (Kaur et al., 2023; Illumina, 2022).

**Quality Control and Preprocessing**: Raw IDAT files were processed in R using the SeSAMe package and the QCDPB workflow (Zhou et al., 2018). This preprocessing pipeline included initial sample quality checks, dye-bias correction, background adjustment, masking of low-quality probes using detection p-values, removal of cross-reactive probes and probes overlapping SNPs, and exclusion of probes located on sex chromosomes. Following preprocessing, methylation levels were expressed as β values.

Effect sizes were derived from the group-level mean β-values for the MDD participants. Pre-treatment fluctuation was calculated as Δβbaseline = T2 − T1, treatment-associated change as Δβtreatment = T3 − T2, and change from the first baseline as ΔβT3–T1 = T3 − T1. The maximum absolute methylation change was calculated as Δmax = max (|T3 − T1|, |T3 − T2|), and baseline stability was evaluated using the ratio R = |T3 − T2| / |T2 − T1|. All methylation analyses reported here were restricted to the 10 MDD participants who completed KAP. Healthy controls were included in the broader study but were not used in the present epigenetic clustering analyses.

**Gene Annotation:** The 1,180 magnitude-filtered CpG loci were mapped to available gene annotations using the Illumina MethylationEPIC v2.0 manifest. Annotation included promoter, intragenic, and intergenic locations. Loci without an available gene annotation were retained in the complete probe-level analysis but excluded from gene-based functional interpretation.

**Software:** Raw methylation data were preprocessed in R using the SeSAMe QCDPB workflow. Downstream probe-level filtering, calculation of methylation changes, stability classification, data summaries, and visualization were performed in R version 4.6.1 using the data.table, ggplot2, ggrastr, openxlsx, lme4, and emmeans packages. The nominal p-values used in the initial T3-versus-T2 screen were generated from participant-matched paired t-tests of methylation β-values. These p-values were subsequently incorporated into the probe-level dataset and were not recalculated during downstream magnitude and stability filtering (Zhou et al., 2018).

The complete R script used for downstream CpG filtering, stability classification, gene-list generation, and visualization is provided as Supplementary Code 1.

All methylation values and statistical comparisons used in the clustering analyses were derived exclusively from the 10 MDD participants across three biological timepoints (T_1_, T_2_, T_3_), with T_3_ representing the single post-treatment sample.

**Code Availability:** The custom R script used for downstream probe-level filtering, calculation of methylation changes, stability classification, gene-list generation, and visualization is provided as Supplementary Code 1.

**Supplementary Table 1**

**Supplementary Table 2**
